# Supplementary material for: Performance of triage systems in emergency care: a systematic review and meta-analysis
Source: BMJ Open. 2019 May 28;9(5):e026471. doi: 10.1136/bmjopen-2018-026471 (PMC6549628; doi:10.1136/bmjopen-2018-026471)
Supplement: Supplementary data [file bmjopen-2018-026471supp001.pdf]

Appendix 1. Search strategy

Embase
(triage* OR ((acuity OR severit* OR priorit* OR critical*) NEAR/3 (scale* OR level* OR index* OR score* OR measure* OR assessment*))):ab,ti AND ('emergency ward'/de OR 'emergency medicine'/de OR 'emergency care'/exp OR (((emergen* OR acute) NEAR/3 (department* OR ward* OR unit* OR room* OR care))):ab,ti) AND (reliability/exp OR reproducibility/de OR 'validation process'/de OR 'validation study'/de OR accuracy/de OR 'sensitivity and specificity'/exp OR 'diagnostic accuracy'/de OR evaluation/de OR validity/exp OR (reliab* OR reproducib* OR validation OR validaty OR consisten* OR variabilit* OR accura* OR 'intra observer' OR intraobserver OR sensitivity OR specificity):ab,ti OR evaluat*:ti) AND [english]/lim

Medline Ovid
(Triage/ OR (triage* OR ((acuity OR severit* OR priorit* OR critical*) ADJ3 (scale* OR level* OR index* OR score* OR measure* OR assessment*))).ab,ti.) AND (exp "Emergency Service, Hospital"/ OR "emergency medicine"/ OR "emergency care"/ OR (((emergen* OR acute) ADJ3 (department* OR ward* OR unit* OR room* OR care))).ab,ti.) AND (Reproducibility of Results/ OR Validation Studies.pt. OR exp "sensitivity and specificity"/ OR Evaluation Studies.pt. OR (reliab* OR reproducib* OR validation OR validaty OR consisten* OR variabilit* OR accura* OR "intra observer" OR intraobserver OR sensitivity OR specificity).ab,ti. OR evaluat*.ti.) AND english.la.

Cochrane central
(triage* OR ((acuity OR severit* OR priorit* OR critical*) NEAR/3 (scale* OR level* OR index* OR score* OR measure* OR assessment*))):ab,ti AND ((((emergen* OR acute) NEAR/3 (department* OR ward* OR unit* OR room* OR care))):ab,ti) AND ((reliab* OR reproducib* OR validation OR validaty OR consisten* OR variabilit* OR accura* OR 'intra observer' OR intraobserver OR sensitivity OR specificity):ab,ti OR evaluat*:ti)

Web-of-science
TS=(triage* OR ((acuity OR severit* OR priorit* OR critical*) NEAR/3 (scale* OR level* OR index* OR score* OR measure* OR assessment*))) AND TS=((((emergen* OR acute) NEAR/3 (department* OR ward* OR unit* OR room* OR care)))) AND (TS=(reliab* OR reproducib* OR validation OR validaty OR consisten* OR variabilit* OR accura* OR "intra observer" OR intraobserver OR sensitivity OR specificity) OR TI=(evaluat*)) AND LA=(English)

CINAHL
(MH Triage+ OR (triage* OR ((acuity OR severit* OR priorit* OR critical*) N3 (scale* OR level* OR index* OR score* OR measure* OR assessment*)))) AND (MH "Emergency Service"+ OR MH "emergency medicine"+ OR (((emergen* OR acute) N3 (department* OR ward* OR unit* OR room* OR care)))) AND (MH Reproducibility of Results+ OR MH Validity+ OR MH "sensitivity and specificity"+ OR MH Evaluation+ OR (reliab* OR reproducib* OR validation OR validaty OR consisten* OR variabilit* OR accura* OR "intra observer" OR intraobserver OR sensitivity OR specificity) OR TI (evaluat*))

Google Scholar
triage emergency|emergencies
reliability|reproducibility|validation|validity|consistency|accuracy|sensitivity|specificity|interobserver |intraobserver

Appendix 2. Selected studies

Table 2.1 Characteristics of included studies

Study	Setting	Annual census	Hospitalization rate	Study population	Triage System	Reference standard (data available)
Aeimchanbanjong, 2017[1]	Single academic hospital, Thailand	10,000	12%	1041 children	ATS, CTS, ESI, MTS, Ramathibodi Triage System	- Hospital admission
Al-Hindi, 2014[2]	Single tertiary maternity and children's hospital, Saudi Arabia	155,000	4%	3,014 children	Ped-CTAS	- Hospital admission<br>- Resource use, other<br>- Waiting time to physician
Allon, 2017[3]	Single tertiary children's hospital, Israel	-	24%	83,609 children	Ped-CTAS	- Hospital admission<br>- ICU admission<br>- Length of stay at the ED<br>- Left without being seen
Baumann, 2005[4]	Single academic hospital, USA	53,000	14%	510 children	ESI v.3	- Hospital admission<br>- Resource use based on ESI<br>- Length of stay at the ED<br>- Mortality at the ED<br>- Mortality after 1 year<br>- Left without being seen
Baumann, 2006[5]	Single academic hospital, USA	53,000	43%	929 elderly	ESI v.3	- Hospital admission<br>- ICU admission<br>- Resource use based on ESI<br>- Length of stay at the ED<br>- Mortality at the ED<br>- Mortality after 1 year<br>- Left without being seen
Brillman, 1996[6]	Single academic hospital,	65,000	6%	5,106 patients	Automated Military	- Hospital admission<br>- Expert opinion

	USA				Outpatient System	
Bruijns, 2008[7]	Single community hospital,<br>South Africa	54,000	40%	798 adults	Cape Triage Score	- Hospital admission<br>- Mortality at the ED
Chang, 2012[8]	Single academic hospital,<br>Taiwan	-	23%	42,346 children	CTAS and Ped-TTS	- Hospital admission<br>- Costs
Chi, 2006[9]	Single academic hospital,<br>Taiwan	58,000	34%	3,172 adults	ESI v.2 and TTS	- Hospital admission<br>- Length of stay at the ED
Dong, 2007[10]	Single tertiary hospital,<br>Canada	67,000	19%	29,524 adults	CTAS	- Hospital admission<br>- Resource use, other<br>- Length of stay at the ED<br>- Mortality at the ED<br>- Costs
Dugas, 2016[11]	Sample of ED visits from 356 hospitals,<br>USA	-	14%	25,198 adults	Combination of triage systems and Electronic emergency Triage System	- Hospital admission<br>- ICU admission<br>- Resource use, other<br>- Resource use based on ESI<br>- Composite outcome: in-hospital mortality, intensive care unit admission or transfer to operating room or catheterization suite
Eitel, 2003[12]	7 community hospitals,<br>USA	28,000-90,000	34% (mean)	1,042 patients	ESI v.2	- Hospital admission<br>- Resource use based on ESI<br>- Length of stay at the ED<br>- Mortality: 60-day mortality
Engan, 2018[13]	Single academic hospital, Norway	4,750	44%	782 children	modified version of the pediatric SATS	- Hospital admission<br>- Resource use, other
Erimsah, 2015[14]	Single academic hospital,	40,000	16%	618 patients	Ministry of Health of	- Hospital admission<br>- Resource use, other

	Turkey				Turkey's mandatory emergency triage instrument	- Length of stay at the ED<br>- Expert opinion<br>- Immediate lifesaving interventions<br>- Mortality: in-hospital mortality
Fan, 2013[15]	Single tertiary hospital,<br>China (Hong Kong)	128,000	-	100 children and adults	Hong Kong Accident and Emergency Triage Guidelines 4th version	- Expert opinion
Fong, 2018[16]	Single community hospital, Singapore	-	25%	300 adults	ESI and Patient Acuity Category Scale	- Hospital admission<br>- Resource use based on ESI
Funakoshi, 2016[17]	Single community hospital,<br>Japan	31,793	-	17,121 adults	Japanese Triage and Acuity Scale	- Hospital admission<br>- Length of stay at the ED<br>- Mortality at the ED
Ganapathy, 2018[18]	Single community hospital, Singapore	-	17%	172,933 children	Singapore Paediatric Triage Scale	- Hospital admission<br>- ICU admission<br>- Length of stay at the ED<br>- Length of stay in hospital
Van Gerven, 2001[19]	Single academic hospital,<br>Belgium	-	45%	3,650 children and adults	Australian National Triage Scale	- Hospital admission<br>- Sentinel diagnosis
Ghafarypour-Jahrom, 2018[20]	Single academic hospital, Iran	45,000	-	1,300 children	ATS and ESI	- Hospital admission
Gouin, 2005[21]	Single academic children's hospital,<br>Canada	65,000	9%	1,281 children	CTAS and local triage tool	- Hospital admission<br>- Resource use, other<br>- PRISA score
Gräff, 2014[22]	Single academic hospital,<br>Germany	23,000	33%	45,469 children and adults	MTS	- Hospital admission<br>- ICU admission<br>- Resource use, other

						- Length of stay at the ED<br>- Mortality: in-hospital mortality<br>- Length of stay in hospital<br>- Level of prehospital care
Gravel, 2009[23]	Single academic hospital,<br>Canada	60,000	9%	5,829 children	Ped-CTAS	- Hospital admission<br>- ICU admission<br>- Length of stay at the ED<br>- Mortality at the ED
Gravel, 2012[24]	9 academic hospitals,<br>Canada	43,962	8%	1,464 children	CTAS	- Hospital admission<br>- Resource use, other<br>- Length of stay at the ED
Gravel, 2013[25]	12 academic children's hospitals,<br>Canada	25,000-75000	9%	550,940 children	CTAS	- Hospital admission<br>- ICU admission<br>- Length of stay at the ED<br>- Left without being seen
Green, 2012[26]	Single academic children's hospital,<br>USA	45,000	10%	780 children	ESI v.4	- Hospital admission<br>- Resource use based on ESI<br>- Length of stay at the ED
Hansen, 2017[27]	Single hospital,<br>Denmark	-	-	550 children	Danish Regions Pediatric Triage model	- Composite outcome "van Veen"; based on abnormal vital parameters, life-threatening conditions, diagnostics, interventions and follow-up<br>- Composite outcome based on life threatening condition, transfer to higher hospital, ICU, or hospital admission
Hay, 2001[28]	Single hospital,<br>Israel	-	-	2,886 patients	Local triage system	- Expert opinion<br>- Waiting time to physician
Van Ierland, 2011[29]	Single academic hospital,<br>the Netherlands	20,000	30% (adults) and 15% (children)	3,207 adults and 701 children	Netherlands Triage System	- Hospital admission<br>- ICU admission<br>- Resource use, other<br>- Follow-up at outpatient clinic or GP

Jobe, 2013[30]	Single academic hospital,<br>Belgium	42,000	36%	544 children and adults	Echelle Liégeoise d'Index de Sévérité à l'Admission	- Hospital admission<br>- ICU admission<br>- Resource use, other<br>- Resource use based on ESI<br>- Mortality at the ED
Lee, 2003[31]	4 hospitals,<br>China (Hong Kong)	-	-	2,410 patients	5-level triage scale	- Expert opinion
Lee, 2011[32]	Single academic hospital,<br>South Korea	60,000	47%	1,903 elderly	CTAS, revised edition	- Hospital admission<br>- ICU admission<br>- Resource use, other<br>- Mortality at the ED<br>- Costs<br>- Immediate lifesaving interventions<br>- Mortality: in-hospital mortality<br>- Length of stay in hospital
Lin, 2016[33]	Single academic hospital,<br>China	217,000	-	275,378 children	Chinese pediatric emergency triage system	- Expert opinion<br>- Waiting time for care<br>- Patients' parents' satisfaction
Ma, 2008[34]	Single academic children's hospital,<br>Canada	50,000	-	1,618 children	Ped-CTAS	- Costs
Maleki, 2015[35]	Single teaching hospital,<br>Iran	74,000	-	387 patients	ESI v.4 and Spot check triage system	- Waiting time to nurse<br>- Waiting time to physician<br>- Waiting time to first lab results
Maningas, 2006[36]	Single community hospital,<br>USA	57,000	6%	7,077 children	Soterion Rapid Triage System	- Hospital admission<br>- Length of stay at the ED<br>- Costs<br>- CPT codes
Maningas, 2006[37]	Single community hospital,<br>USA	57,000	10%	33,850 adults	Soterion Rapid Triage System	- Hospital admission<br>- Length of stay at the ED<br>- Costs<br>- CPT codes

Martins, 2009[38]	Single community hospital, Portugal	128,000	10%	316,622 adults	MTS	- Hospital admission<br>- Mortality at the ED
Meyer, 2018[39]	Single community hospital, South Africa	-	22%	4,002 patients	SATS	- Hospital admission
Ng, 2010[40]	3 hospitals, Taiwan	-	34%	1,851 adults	CTAS and Taiwan Triage System	- Hospital admission<br>- Length of stay at the ED<br>- Costs
Ng, 2011[41]	11 academic hospitals, 18 regional hospitals and 4 district hospitals, Taiwan	95,912 (average, university hospitals); 47,056 (average regional hospitals); 23,789 (average district hospitals	30%	10,533 adults	Taiwan Triage and Acuity Scale and Taiwan Triage System	- Hospital admission<br>- Resource use based on ESI<br>- Length of stay at the ED
Platts-Mills, 2010[42]	Single academic hospital, USA	64,000	58%	773 elderly	ESI v.4	- Immediate lifesaving interventions
Roukema, 2006[43]	Single teaching hospital, the Netherlands	30,000	-	1,065 children	MTS	- Hospital admission<br>- Resource use based on ESI<br>- Composite outcome “van Veen”; based on abnormal vital parameters, life-threatening conditions, diagnostics, interventions and follow-up
Santos, 2013[44]	Single hospital, Portugal	-	5%	24,721 adults	MTS v.2	- Hospital admission<br>- Resource use, other<br>- Mortality: in-hospital mortality

Seiger, 2014[45]	2 academic and 2 teaching hospitals, the Netherlands, UK and Portugal	9000-60,000	11% (average)	60,735 children	MTS	- Hospital admission
Steiner, 2016[46]	Single tertiary hospital, Switzerland	-	-	2,407 adults	MTS	- ICU admission<br>- Expert opinion<br>- Mortality: 30 day mortality<br>- Length of stay in hospital
Storm-Versloot, 2011[47]	Single academic hospital, the Netherlands	31,000	18%	876 patients	ESI, MTS and informally structured triage system	- Hospital admission<br>- Resource use based on ESI<br>- Length of stay at the ED<br>- Expert opinion
Storm- Versloot, 2014[48]	Single academic hospital, the Netherlands	31,000	21%	906 patients	MTS and informally structured triage system	- Length of stay at the ED<br>- Waiting time from arrival to treatment room<br>- Waiting time from treatment room to discharge from ED<br>- Patient satisfaction
Taboulet, 2009[49]	Single teaching hospital, France	30,000	18%	941 adults	FRench Emergency Nurses Classification in Hospital Scale v.2	- Hospital admission<br>- Resource use, other<br>- Waiting time to physician
Tanabe, 2004[50]	Single academic hospital, USA	70,000	-	403 children and adults	ESI v.3	- Resource use, other<br>- Length of stay at the ED<br>- Length of stay in hospital
Tanabe, 2004[51]	Single academic hospital, USA	70,000	-	403 children and adults	ESI v.3	- Hospital admission<br>- Resource use based on ESI<br>- Length of stay at the ED
Travers, 2002[52]	Single academic hospital, USA	-	-	305 patients	ESI and 3-level system	- Expert opinion
Travers, 2009[53]	1 academic hospital	13,838-	-	1,173	ESI v.4	- Hospital admission

	and 4 teaching/community hospitals, USA	44,987		children		- Resource use based on ESI<br>- Length of stay at the ED
Twomey, 2013[54]	1 private and 5 public community hospitals, South Africa	-	26%	2,014 children	revised paediatric SATS	- Hospital admission
Veen, van, 2008[55]	1 academic children's and 1 teaching hospital, the Netherlands	9,000-30,000	-	13,554 children	MTS v.1	- Composite outcome "van Veen"; based on abnormal vital parameters, life-threatening conditions, diagnostics, interventions and follow-up
Veen, van, 2012[56]	1 academic children's and 1 teaching hospital, the Netherlands	9,000-15,000	-	11,260 children	MTS incl. modifications for children with fever	- Composite outcome "van Veen"; based on abnormal vital parameters, life-threatening conditions, diagnostics, interventions and follow-up
Waldrop, 1997[57]	Single teaching hospital, USA	65,000	5% (adults), 8% (children)	13,294 adults and 1528 children	Local triage system	- Hospital admission<br>- Mortality at the ED<br>- Length of stay at the ED<br>- Left without being seen<br>- Waiting time to examination
Worster, 2007[58]	2 academic hospitals, Canada	36,000-395,000	-	481 patients	CTAS and ESI	- Resource use, other<br>- Mortality: in-hospital mortality
Wuerz, 2000[59]	2 academic hospitals, USA	60,000 (average)	32%	493 adults	ESI	- Hospital admission<br>- Resource use based on ESI<br>- Length of stay at the ED<br>- Costs
Wuerz, 2001[60]	Single academic hospital, USA	50,000	-	180 adults	ESI	- Mortality: 6 month mortality
Wuerz, 2001[61]	2 academic hospitals, USA	50,000-60,000	28%	8,251 adults	ESI	- Hospital admission<br>- Length of stay at the ED

Wulp, van der 2009[62]	4 teaching hospitals, the Netherlands	30,000-47,000	21%	72,232 children and adults	ESI and MTS	- Hospital admission<br>- Mortality at the ED
Wulp, van der, 2011[63]	Single academic hospital, the Netherlands	21,000	-	544 adults	ESI	- Worthing Phsyiological Scoring System<br>- Pain score
Yuksen, 2016[64]	Single academic hospital, Thailand	-	-	520 patients	ESI and 4-level nurse triage	- Hospital admission<br>- Resource use based on ESI<br>- Length of stay at the ED<br>- Mortality at the ED<br>- Immediate lifesaving interventions
Zachariasse, 2016[65]	Single academic children's hospital, the Netherlands	7,000	-	50,062 children	MTS	- ICU admission
Zachariasse, 2017[66]	1 academic children's hospital, 1 teaching hospital and 1 community hospital, the Netherlands and Portugal	7,000 – 190,000	-	222,603 adults and 66,060 children	MTS	- ICU admission<br>- Composite outcome based on physiologic parameters, patient disposition and resource use

ATS, Australasian Triage Scale; *CTAS*, Canadian Triage and Acuity Scale; *ESI*, Emergency Severity Index; *MTS*, Manchester Triage System; *Ped*, Pediatric; *SATS,* South African Triage Scale; *TTS*, Taiwan Triage System;

References

1. Aeimchanbanjong K, Pandee U. Validation of different pediatric triage systems in the emergency department. *World J Emerg Med.* 2017;8:223-227.
2. Al-Hindi AA, Al-Akhfash AA, Fareed AM, Alhusainan KS, Algasomy SF, Althowainy IR. Efficacy of implementation of a 5 scale pediatric triage and acuity scale in pediatric emergency, Saudi Arabia. *Saudi Medical Journal.* 2014;35:999-1004.
3. Allon R, Feldman O, Karminsky A, Steinberg C, Leiba R, Shavit I. Validity of the Pediatric Canadian Triage Acuity Scale in a tertiary children's hospital in Israel. *Eur J Emerg Med.* 2017.
4. Baumann MR, Strout TD. Evaluation of the Emergency Severity Index (version 3) triage algorithm in pediatric patients. *Acad Emerg Med.* 2005;12:219-224.
5. Baumann MR, Strout TD. Triage of Geriatric Patients in the Emergency Department: Validity and Survival With the Emergency Severity Index. *Ann Emerg Med.* 2007;49:234-240.
6. Brillman JC, Doezema D, Tandberg D, et al. Triage: Limitations in predicting need for emergent care and hospital admission. *ANN EMERG MED.* 1996;27:493-500.
7. Bruijns SR, Wallis LA, Burch VC. A prospective evaluation of the Cape triage score in the emergency department of an urban public hospital in South Africa. *Emerg Med J.* 2008;25:398-402.
8. Chang YC, Ng CJ, Wu CT, Chen LC, Chen JC, Hsu KH. Effectiveness of a five-level Paediatric Triage System: An analysis of resource utilisation in the emergency department in Taiwan. *Emerg Med J.* 2012.
9. Chi CH, Huang CM. Comparison of the Emergency Severity Index (ESI) and the Taiwan triage system in predicting resource utilization. *J Formos Med Assoc.* 2006;105:617-625.
10. Dong SL, Bullard MJ, Meurer DP, et al. Predictive Validity of a Computerized Emergency Triage Tool. *Acad Emerg Med.* 2007;14:16-21.
11. Dugas AF, Kirsch TD, Toerper M, et al. An Electronic Emergency Triage System to Improve Patient Distribution by Critical Outcomes. *J Emerg Med.* 2016;50:910-918.
12. Eitel DR, Travers DA, Rosenau AM, Gilboy N, Wuerz RC. The Emergency Severity Index triage algorithm version 2 is reliable and valid. *Acad Emerg Med.* 2003;10:1070-1080.
13. Engan M, Hirth A, Tronnes H. Validation of a Modified Triage Scale in a Norwegian Pediatric Emergency Department. *Int J Pediatr.* 2018;2018:4676758.
14. Erimşah ME, Yaka E, Yilmaz S, Kama A, Pekdemir M. Inter-rater reliability and validity of the Ministry of Health of Turkey's mandatory emergency triage instrument. *EMA Emerg Med Australas.* 2015;27:210-215.
15. Fan MMW, Leung LP. Validation of the Hong Kong accident and Emergency Triage Guidelines. *Hong Kong Med J.* 2013;19:198-202.
16. Fong RY, Glen WSS, Mohamed Jamil AK, Tam WWS, Kowitlawakul Y. Comparison of the Emergency Severity Index versus the Patient Acuity Category Scale in an emergency setting. *Int Emerg Nurs.* 2018;41:13-18.
17. Funakoshi H, Shiga T, Homma Y, et al. Validation of the modified Japanese Triage and Acuity Scale-based triage system emphasizing the physiologic variables or mechanism of injuries. *Int J Emer Med.* 2016;9:1-6.

18. Ganapathy S, Yeo JG, Thia XHM, Hei GMA, Tham LP. The Singapore Paediatric Triage Scale Validation Study. *Singapore Med J.* 2018;59:205-209.
19. Van Gerven R, Delooz H, Sermeus W. Systematic triage in the emergency department using the Australian National Triage Scale: a pilot project. *Eur J Emerg Med.* 2001;8:3-7.
20. Ghafarypour-Jahrom M, Taghizadeh M, Heidari K, Derakhshanfar H. Validity and Reliability of the Emergency Severity Index and Australasian Triage System in Pediatric Emergency Care of Mofid Children's Hospital in Iran. *Bull Emerg Trauma.* 2018;6:329-333.
21. Gouin S, Gravel J, Amre DK, Bergeron S. Evaluation of the Paediatric Canadian Triage and Acuity Scale in a pediatric ED. *Am J Emerg Med.* 2005;23:243-247.
22. Gräff I, Goldschmidt B, Glien P, et al. The German version of the Manchester triage system and its quality criteria - First assessment of validity and reliability. *PLoS ONE.* 2014;9.
23. Gravel J, Manzano S, Arsenault M. Validity of the Canadian Paediatric Triage and Acuity Scale in a tertiary care hospital. *Can J Emerg Med.* 2009;11:23-28.
24. Gravel J, Gouin S, Goldman RD, et al. The Canadian Triage and Acuity Scale for children: A prospective multicenter evaluation. *Ann Emerg Med.* 2012;60:71-77.
25. Gravel J, Fitzpatrick E, Gouin S, et al. Performance of the Canadian Triage and Acuity Scale for children: a multicenter database study. *Ann Emerg Med.* 2013;61:27-32 e23.
26. Green NA, Durani Y, Brecher D, Depiero A, Loiselle J, Attia M. Emergency severity index version 4: A valid and reliable tool in pediatric emergency department triage. *Pediatr Emerg Care.* 2012;28:753-757.
27. Hansen LH, Mogensen CB, Wittenhoff L, Skjot-Arkil H. The danish regions pediatric triage model has a limited ability to detect both critically ill children as well as children to be sent home without treatment - a study of diagnostic accuracy. *Scand J Trauma Resusc Emerg Med.* 2017;25:55.
28. Hay E, Bekerman L, Rosenberg G, Peled R. Quality assurance of nurse triage: Consistency of results over three years. *Am J Emerg Med.* 2001;19:113-117.
29. van Ierland Y, van Veen M, Huibers L, Giesen P, Moll HA. Validity of telephone and physical triage in emergency care: The Netherlands Triage System. *Fam Pract.* 2011;28:334-341.
30. Jobe J, Ghuysen A, Gerard P, Hartstein G, D'Orio V. Reliability and validity of a new French-language triage algorithm: The ELISA scale. *Emerg Med J.* 2013.
31. Lee A, Hazlett CB, Chow S, et al. How to minimize inappropriate utilization of Accident and Emergency Departments: Improve the validity of classifying the general practice cases amongst the A&E attendees. *Health Policy.* 2003;66:159-168.
32. Lee JY, Oh SH, Peck EH, et al. The validity of the Canadian Triage and Acuity Scale in predicting resource utilization and the need for immediate life-saving interventions in elderly emergency department patients. *Scand J Trauma Resusc Emerg Med.* 2011;19:68.
33. Lin GX, Yang YL, Kudirka D, et al. Implementation of a Pediatric Emergency Triage System in Xiamen, China. *Chin Med J (Engl).* 2016;129:2416-2421.
34. Ma W, Gafni A, Goldman RD. Correlation of the Canadian Pediatric Emergency Triage and Acuity Scale to ED resource utilization. *Am J Emerg Med.* 2008;26:893-897.

35. Maleki M, Fallah R, Riahi L, Delavari S, Rezaei S. Effectiveness of Five-Level Emergency Severity Index Triage System Compared With Three-Level Spot Check: An Iranian Experience. *Arch Trauma Res.* 2015;4:e29214.

36. Maningas PA, Hime DA, Parker DE. The use of the soterion rapid triage system in children presenting to the Emergency Department. *J Emerg Med.* 2006;31:353-359.

37. Maningas PA, Hime DA, Parker DE, McMurry TA. The Soterion Rapid Triage System: Evaluation of inter-rater reliability and validity. *J Emerg Med.* 2006;30:461-469.

38. Martins HM, Cuna LM, Freitas P. Is Manchester (MTS) more than a triage system? A study of its association with mortality and admission to a large Portuguese hospital. *Emerg Med J.* 2009;26:183-186.

39. Meyer GD, Meyer TN, Gaunt CB. Validity of the South African Triage Scale in a rural district hospital. *Afr J Emerg Med.* 2018;8:145-149.

40. Ng CJ, Hsu KH, Kuan JT, et al. Comparison between Canadian Triage and Acuity Scale and Taiwan Triage System in emergency departments. *J Formos Med Assoc.* 2010;109:828-837.

41. Ng CJ, Yen ZS, Tsai JCH, et al. Validation of the Taiwan triage and acuity scale: A new computerised five-level triage system. *Emerg Med J.* 2011;28:1026-1031.

42. Platts-Mills TF, Travers D, Biese K, et al. Accuracy of the emergency severity index triage instrument for identifying elder emergency department patients receiving an immediate life-saving intervention. *Acad Emerg Med.* 2010;17:238-243.

43. Roukema J, Steyerberg EW, van Meurs A, Ruige M, van der Lei J, Moll HA. Validity of the Manchester Triage System in paediatric emergency care. *Emerg Med J.* 2006;23:906-910.

44. Santos AP, Freitas P, Martins HMG. Manchester triage system version II and resource utilisation in emergency department. *Emerg Med J.* 2013.

45. Seiger N, van Veen M, Almeida H, et al. Improving the Manchester Triage System for pediatric emergency care: an international multicenter study. *PLoS One.* 2014;9:e83267.

46. Steiner D, Renetseder F, Kutz A, et al. Performance of the Manchester Triage System in Adult Medical Emergency Patients: A Prospective Cohort Study. *J Emerg Med.* 2016;50:678-689.

47. Storm-Versloot MN, Ubbink DT, Kappelhof J, Luitse JSK. Comparison of an informally structured triage system, the emergency severity index, and the manchester triage system to distinguish patient priority in the emergency department. *Acad Emerg Med.* 2011;18:822-829.

48. Storm-Versloot MN, Vermeulen H, van Lammeren N, Luitse JS, Goslings JC. Influence of the Manchester triage system on waiting time, treatment time, length of stay and patient satisfaction; a before and after study. *Emerg Med J.* 2014;31:13-18.

49. Taboulet P, Moreira V, Haas L, et al. Triage with the French Emergency Nurses Classification in Hospital scale: Reliability and validity. *Eur J Emerg Med.* 2009;16:61-67.

50. Tanabe P, Gimbel R, Yarnold PR, Adams JG. The Emergency Severity Index (version 3) 5-level triage system scores predict ED resource consumption. *J Emerg Nurs.* 2004;30:22-29.

51. Tanabe P, Gimbel R, Yarnold PR, Kyriacou DN, Adams JG. Reliability and Validity of Scores on the Emergency Severity Index Version 3. *Acad Emerg Med.* 2004;11:59-65.

52. Travers DA, Waller AE, Bowling JM, Flowers D, Tintinalli J. Five-level triage system more effective than three-level in tertiary emergency department. *J Emerg Nurs.* 2002;28:395-400.

53. Travers DA, Waller AE, Katznelson J, Agans R. Reliability and validity of the emergency severity index for pediatric triage. *Acad Emerg Med.* 2009;16:843-849.

54. Twomey M, Cheema B, Buys H, et al. Vital signs for children at triage: A multicentre validation of the revised South African Triage Scale (SATS) for children. *Samj South African Medical Journal.* 2013;103:304-308.

55. van Veen M, Steyerberg EW, Ruige M, et al. Manchester triage system in paediatric emergency care: prospective observational study. *BMJ.* 2008;337:a1501.

56. Van Veen M, Steyerberg EW, Van't Klooster M, et al. The Manchester triage system: Improvements for paediatric emergency care. *Emerg Med J.* 2012;29:654-659.

57. Waldrop RD, Harper DE, Mandry C. Prospective assessment of triage in an urban emergency department. *SOUTH MED J.* 1997;90:1208-1212.

58. Worster A, Fernandes CM, Eva K, Upadhye S. Predictive validity comparison of two five-level triage acuity scales. *Eur J Emerg Med.* 2007;14:188-192.

59. Wuerz RC, Milne LW, Eitel DR, Travers D, Gilboy N. Reliability and validity of a new five-level triage instrument. *Acad Emerg Med.* 2000;7:236-242.

60. Wuerz R, Eitel D, Gilboy N, et al. Emergency severity index triage category is associated with six-month survival. *Acad Emerg Med.* 2001;8:61-64.

61. Wuerz RC, Travers D, Gilboy N, Eitel DR, Rosenau A, Yazhari R. Implementation and refinement of the Emergency severity index. *Acad Emerg Med.* 2001;8:170-176.

62. van der Wulp I, Schrijvers AJP, van Stel HF. Predicting admission and mortality with the Emergency Severity Index and the Manchester Triage System: a retrospective observational study. *Emergency Medicine Journal.* 2009;26:506-509.

63. Van Der Wulp I, Rullmann HAA, Leenen LPH, Van Stel HF. Associations of the Emergency Severity Index triage categories with patients' vital signs at triage: A prospective observational study. *Emerg Med J.* 2011;28:1032-1035.

64. Yuksen C, Sawatmongkornkul S, Suttabuth S, Sawanyawisuth K, Sittichanbuncha Y. Emergency severity index compared with 4-level triage at the emergency department of Ramathibodi University Hospital. *Asian Biomed.* 2016;10:155-161.

65. Zachariasse JM, Kuiper JW, de Hoog M, Moll HA, van Veen M. Safety of the Manchester Triage System to Detect Critically Ill Children at the Emergency Department. *J Pediatr.* 2016;177:232-237.e231.

66. Zachariasse JM, Seiger N, Rood PPM, et al. Validity of the Manchester triage system in emergency care: A prospective observational study. *PLoS ONE.* 2017;12.

Appendix 3. Risk of bias assessment

Table 3.1 Risk of bias and applicability concerns summary: review authors' judgements about each domain for each included study

	Risk of Bias				Applicability Concerns		
	Patient Selection	Index Test	Reference Standard	Flow and Timing	Patient Selection	Index Test	Reference Standard
Aeimchanbanjong 2017	−	−	−	+	+	−	?
Al-Hindi 2014	+	+	−	−	+	+	?
Allon 2017	+	+	+	+	+	+	?
Baumann 2005	+	+	+	+	+	+	?
Baumann 2006	+	+	+	+	−	+	?
Brillman 1996	+	+	−	−	+	−	?
Bruijns 2008	+	+	+	−	+	+	?
Chang 2013	+	+	+	+	−	+	?
Chi 2006	−	−	−	−	+	−	?
Dong 2007	+	?	+	+	+	+	?
Dugas 2016	+	−	+	−	−	−	?
Eitel 2003	+	+	−	+	+	+	?
Engan 2018	+	−	−	−	+	+	?
Erimsah 2015	+	+	?	−	+	−	?
Fan 2012	+	+	+	+	+	+	?
Fong 2018	+	−	+	−	+	+	?
Funakoshi 2016	+	+	+	+	+	+	?
Ganapathy 2018	+	+	+	+	+	+	?
Gerven, van 2001	+	−	−	+	+	+	?
Ghafarypour-Jahrom 2018	?	−	−	+	+	+	?
Gouin 2005	+	+	−	+	+	+	?
Graff 2014	+	+	+	+	−	+	?
Gravel 2009	+	?	+	+	+	+	?
Gravel 2012	−	+	+	+	+	+	?
Gravel 2013	+	+	+	+	+	+	?
Green 2012	+	+	−	+	+	+	?
Hansen 2017	−	−	+	−	+	+	?
Hay 2001	−	+	−	−	+	−	?
Ierland van 2011	+	+	+	−	+	+	?
Jobe 2013	+	?	+	?	+	+	?
Lee 2003	−	+	+	−	?	?	?
Lee 2011	+	+	+	+	+	+	?
Lin 2016	+	+	−	+	+	+	?
Ma 2008	+	+	+	+	+	+	?
Maleki 2015	+	+	?	−	+	+	?
Maningas 2006	−	+	?	+	+	+	?
Maningas 2006b	−	+	+	+	+	+	?
Martins 2009	+	+	+	+	+	+	?
Meyer 2018	+	+	?	+	+	+	?
Ng 2010	+	−	?	+	+	+	?
Ng 2011	−	+	+	−	+	+	?
Platts-Mills 2010	+	+	−	+	+	+	?
Roukema 2006	+	+	+	+	+	+	?
Santos 2013	−	+	+	+	+	+	?
Seiger 2014	+	+	+	+	+	+	?
Steiner 2016	+	+	+	+	−	+	?
Storm-Versloot 2011	+	−	?	−	+	+	?
Storm-Versloot 2014	+	+	?	−	+	+	?
Taboulet 2009	−	+	?	?	+	+	?
Tanabe 2004	+	−	+	+	+	+	?
Tanabe 2004b	+	−	+	+	+	+	?
Travers 2002	+	+	+	+	+	+	?
Travers 2009	+	+	−	+	+	+	?
Twomey 2013	+	+	−	−	+	+	?
Veen, van 2008	+	+	+	−	+	+	?
Veen, van 2012	+	+	+	−	+	+	?
Waldrop 1997	+	+	+	?	+	+	?
Worster 2007	+	−	+	+	+	+	?
Wuerz 2000	−	−	+	−	+	+	?
Wuerz 2001	+	+	+	−	+	+	?
Wuerz 2001b	+	+	+	−	+	+	?
Wulp, van der 2009	+	+	?	+	+	+	?
Wulp, van der 2011	+	+	?	−	+	+	?
Yuksen 2016	−	+	−	−	−	−	?
Zachariasse 2016	+	+	+	−	+	+	?
Zachariasse 2017	+	+	+	−	+	+	?

− High ? Unclear + Low

Appendix 4. Reference standards used in studies evaluating triage systems in the emergency department

**Patient disposition and follow-up**	Hospital admission<br>ICU admission<br>Follow-up at outpatient clinic or GP	47 studies<br>13 studies<br>1 study
**Waiting times**	Length of stay at the ED<br>Length of stay in hospital<br>Waiting time to physician<br>Other: Waiting time to nurse, Waiting time to examination, Waiting time to first lab results, Waiting time from arrival to treatment room, Waiting time from treatment room to discharge from ED, Waiting time for care	27 studies<br>5 studies<br>4 studies<br>1 study each
**Resource use**	Resource use based on ESI criteria<br>Costs<br>Current Procedural Terminology (CPT) codes<br>Other	14 studies<br>8 studies<br>2 studies<br>15 studies
**Mortality**	Mortality at the ED<br>In-hospital mortality<br>1 year mortality<br>Other: 30-day mortality, 60-day mortality, 6 month mortality	12 studies<br>5 studies<br>2 studies<br>1 study each
**Composite outcomes**	"Immediate lifesaving interventions"<br>"MTS reference standard": Combination of abnormal physiologic parameters, life-threatening conditions, resource use, follow-up<br>"Critical or time-sensitive outcomes": in-hospital mortality, intensive care unit admission or transfer to operating room or catheterization suite<br>"Actual clinical outcomes": Combination of life threatening condition, transfer to higher hospital, ICU, or hospital admission<br>Composite outcome based on physiologic parameters, patient disposition and resource use	4 studies<br>4 studies<br>1 study<br>1 study<br>1 study
**Physiologic parameters**	Worthing Phsyiological Scoring System<br>Pain score	1 study<br>1 study
**Other**	Expert opinion<br>Left without being seen<br>Patient or parent satisfaction<br>Level of prehospital care<br>Sentinel diagnosis<br>Pediatric risk of admission (PRISA) score	9 studies<br>5 studies<br>2 studies<br>1 study<br>1 study<br>1 study

Appendix 5. Reference standard per urgency category

Figure 5.1 Proportion of ICU admissions per urgency category in the most commonly evaluated triage systems.

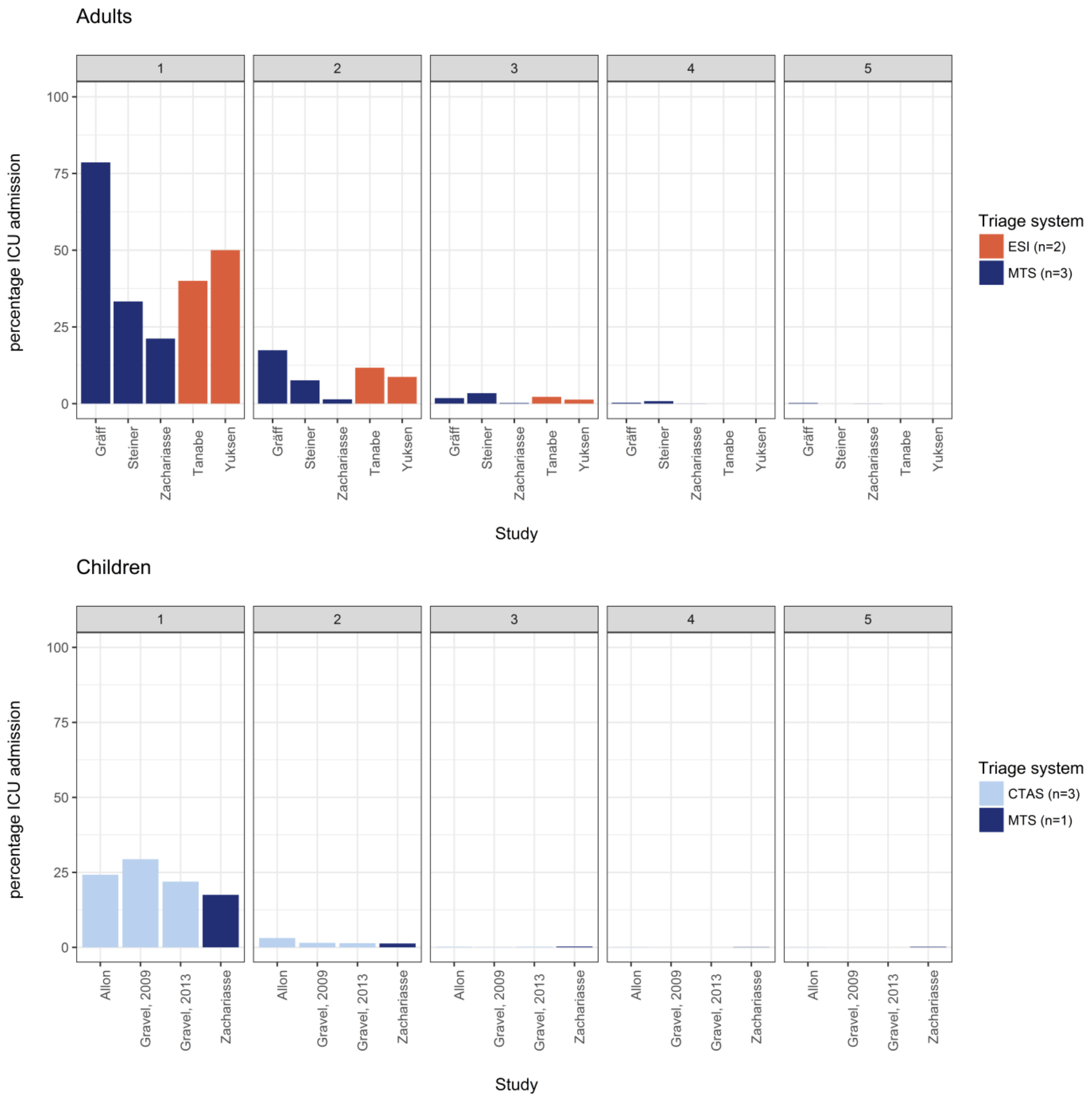

Figure 5.2 Proportion of patients discharged home per urgency category in the most commonly evaluated triage systems

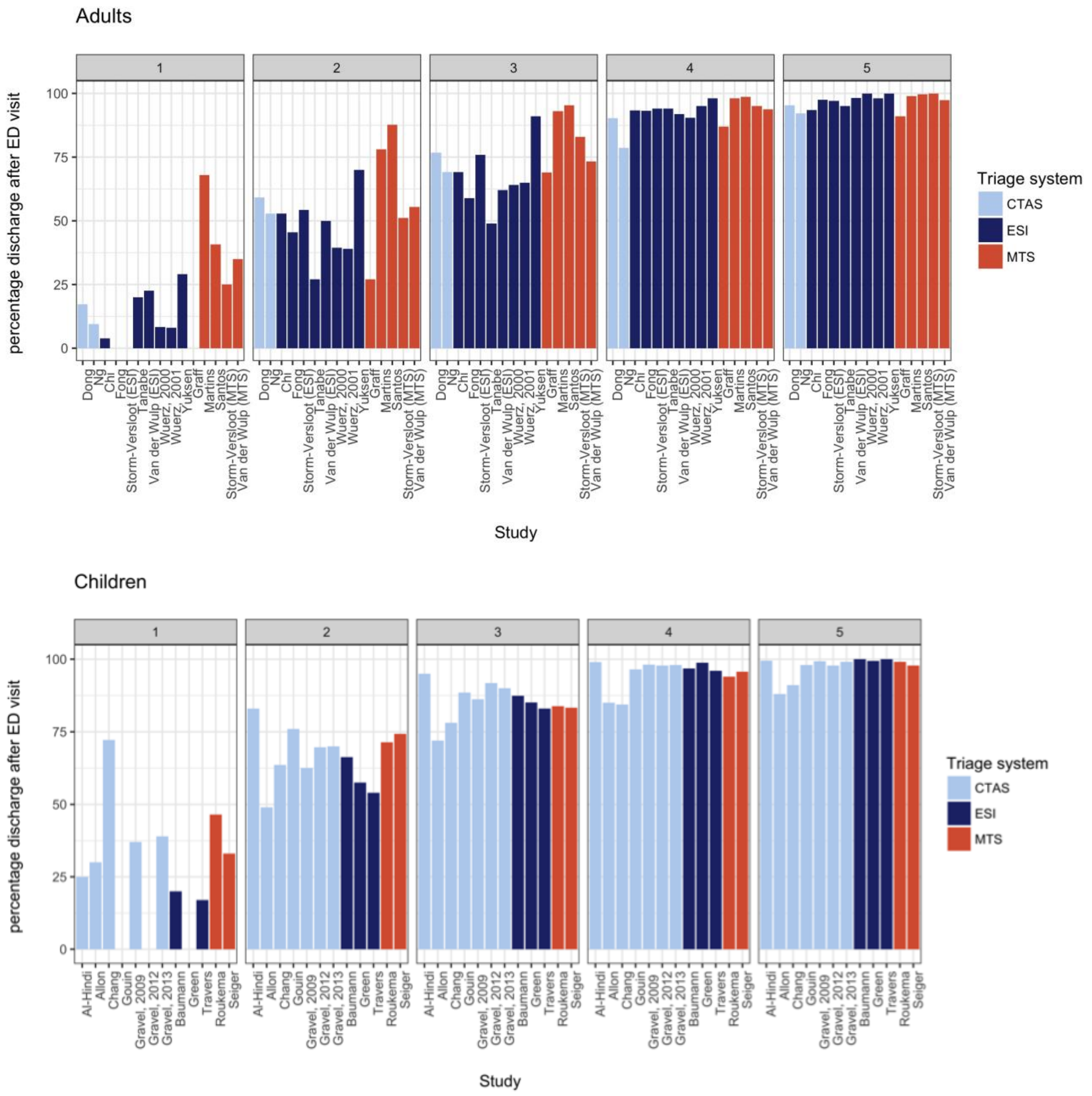

Appendix 6. Other reference standards

Other reference standards

14 studies used resource use according to ESI criteria as a reference standard of which 7 provided data to construct a 2x2 table and were conducted in one of the commonly used triage systems. One study evaluated resource use in the MTS, and all others studied the ESI.

Because the ESI states that resource prediction should only be used for the less acute patients, we used a different cut-off for dichotomization to calculate sensitivity and specificity. We dichotomized the triage categories into a high urgent group consisting of triage urgency levels 1, 2 and 3 with a low urgent group consisting of triage categories 4 and 5.

Overall, sensitivity of the triage systems to accurately classify patients with 2 or more resources in the highest urgency categories ranged from 0.75 (95%CI 0.68 to 0.81) to 0.91 (95%CI 0.87 to 0.94) in adults and from 0.76 (95%CI 0.70 to 0.82) to 0.90 (95%CI 0.87 to 0.92) in children (Fig. 7). Specificity of the triage system to accurately classify patients with 1 or 0 resources in the lowest two urgency categories ranged from 0.73 (95%CI 0.66 to 0.79) to 0.81 (95%CI 0.73 to 0.88) in adults and from 0.42 (95%CI 0.39 to 0.46) to 0.75 (95%CI 0.69 to 0.81) in children.

Fig 6.1. Forest plot evaluating sensitivity and specificity of triage systems using resource use according to ESI criteria as reference standard

ESI

Study	TP	FP	FN	TN	Age group	Sensitivity (95% CI)	Specificity (95% CI)
Baumann 2005	101	183	11	215	Children	0.90 [0.83, 0.95]	0.54 [0.49, 0.59]
Travers 2009	480	200	56	438	Children	0.90 [0.87, 0.92]	0.69 [0.65, 0.72]
Green 2012	158	206	49	367	Children	0.76 [0.70, 0.82]	0.64 [0.60, 0.68]
Wuerz 2000	241	59	36	157	Adults / Unspecified	0.87 [0.82, 0.91]	0.73 [0.66, 0.79]
Yuksen 2016	278	53	29	160	Adults / Unspecified	0.91 [0.87, 0.94]	0.75 [0.69, 0.81]
Fong 2018	134	23	45	98	Adults / Unspecified	0.75 [0.68, 0.81]	0.81 [0.73, 0.88]

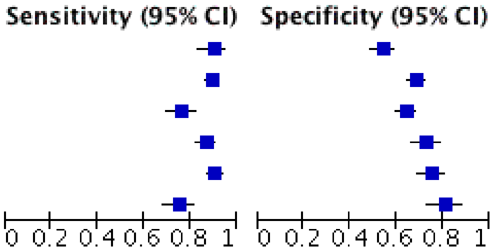

MTS

Study	TP	FP	FN	TN	Age group	Sensitivity (95% CI)	Specificity (95% CI)
Roukema 2006	205	469	51	340	Children	0.80 [0.75, 0.85]	0.42 [0.39, 0.46]

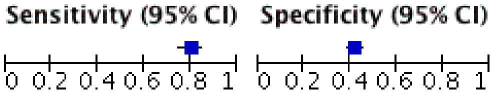

ESI = Emergency Severity Index; TP = true positive; FP = false positive; FN = false negative; TN = true negative; MTS = Manchester Triage System

Appendix 7. Determinants of triage systems' performance

Figure 7.1. Forest plot evaluating sensitivity and specificity of triage systems for identifying low urgency patients as defined by discharge home after the ED visit, ordered by annual census as a marker of patient volume

Adults

Study	TP	FP	FN	TN	Annual Census	Sensitivity (95% CI)	Specificity (95% CI)
Graff 2014	6497	2132	8463	28377	23000.0	0.43 [0.43, 0.44]	0.93 [0.93, 0.93]
Storm-Versloot 2011	66	76	96	638	31000.0	0.41 [0.33, 0.49]	0.89 [0.87, 0.92]
Storm-Versloot 2011	73	72	76	651	31000.0	0.49 [0.41, 0.57]	0.90 [0.88, 0.92]
Yuksen 2016	38	68	22	392	33936.0	0.63 [0.50, 0.75]	0.85 [0.82, 0.88]
Van der Wulp 2009	2259	2146	5911	27658	38500.0	0.28 [0.27, 0.29]	0.93 [0.93, 0.93]
Van der Wulp 2009	2641	3183	4415	24019	38500.0	0.37 [0.36, 0.39]	0.88 [0.88, 0.89]
Wuerz 2001	1113	674	1260	5204	55000.0	0.47 [0.45, 0.49]	0.89 [0.88, 0.89]
Chi 2006	713	747	376	1336	58000.0	0.65 [0.63, 0.68]	0.64 [0.62, 0.66]
Wuerz 2000	91	53	68	281	60000.0	0.57 [0.49, 0.65]	0.84 [0.80, 0.88]
Dong 2007	1519	1947	4189	21869	67000.0	0.27 [0.25, 0.28]	0.92 [0.91, 0.92]
Tanabe 2004	129	48	75	151	70000.0	0.63 [0.56, 0.70]	0.76 [0.69, 0.82]
Santos 2013	485	3080	535	19515	100000.0	0.48 [0.44, 0.51]	0.86 [0.86, 0.87]
Martins 2009	18265	63803	12464	222090	128000.0	0.59 [0.59, 0.60]	0.78 [0.78, 0.78]

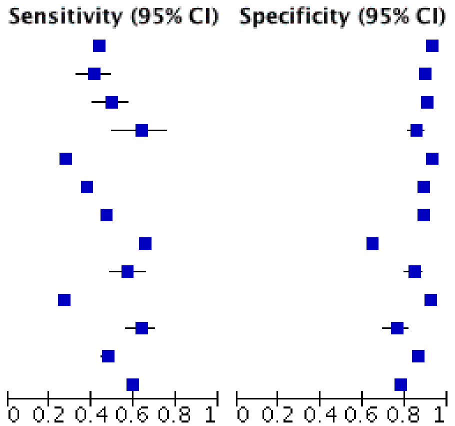

Children

Study	TP	FP	FN	TN	Annual Census	Sensitivity (95% CI)	Specificity (95% CI)
Aeimchanbanjong 2017	62	239	62	679	10000.0	0.50 [0.41, 0.59]	0.74 [0.71, 0.77]
Aeimchanbanjong 2017	64	165	59	753	10000.0	0.52 [0.43, 0.61]	0.82 [0.79, 0.84]
Aeimchanbanjong 2017	70	285	53	633	10000.0	0.57 [0.48, 0.66]	0.69 [0.66, 0.72]
Allon 2017	2631	2330	17726	60922	16722.0	0.13 [0.12, 0.13]	0.96 [0.96, 0.96]
Roukema 2006	147	256	62	600	30000.0	0.70 [0.64, 0.76]	0.70 [0.67, 0.73]
Chang 2013	3104	6821	6276	26327	42839.0	0.33 [0.32, 0.34]	0.79 [0.79, 0.80]
Green 2012	33	42	47	658	45000.0	0.41 [0.30, 0.53]	0.94 [0.92, 0.96]
Baumann 2005	44	66	28	372	53000.0	0.61 [0.49, 0.72]	0.85 [0.81, 0.88]
Gravel 2009	1937	2746	3124	50722	60000.0	0.38 [0.37, 0.40]	0.95 [0.95, 0.95]
Gouin 2005	8	19	38	472	65000.0	0.17 [0.08, 0.31]	0.96 [0.94, 0.98]
Al-Hindi 2014	65	295	45	2609	155000.0	0.59 [0.49, 0.68]	0.90 [0.89, 0.91]

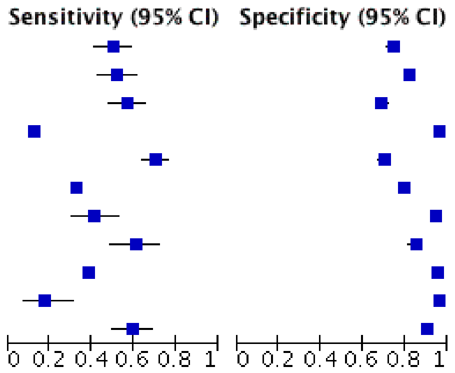

TP = true positive; FP = false positive; FN = false negative; TN = true negative

Figure 7.2. Forest plot evaluating sensitivity and specificity of triage systems for identifying low urgency patients as defined by discharge home after the ED visit, ordered by percentage hospitalization as a marker of case-mix severity of illness

Adults

Study	TP	FP	FN	TN	Hospitalization rate	Sensitivity (95% CI)	Specificity (95% CI)
Santos 2013	485	3080	535	19515	5.0	0.48 [0.44, 0.51]	0.86 [0.86, 0.87]
Martins 2009	18265	63803	12464	222090	10.0	0.59 [0.59, 0.60]	0.78 [0.78, 0.78]
Yuksen 2016	38	68	22	392	12.0	0.63 [0.50, 0.75]	0.85 [0.82, 0.88]
Storm-Versloot 2011	66	76	96	638	18.0	0.41 [0.33, 0.49]	0.89 [0.87, 0.92]
Storm-Versloot 2011	73	72	76	651	18.0	0.49 [0.41, 0.57]	0.90 [0.88, 0.92]
Dong 2007	1519	1947	4189	21869	19.0	0.27 [0.25, 0.28]	0.92 [0.91, 0.92]
Van der Wulp 2009	2259	2146	5911	27658	21.0	0.28 [0.27, 0.29]	0.93 [0.93, 0.93]
Van der Wulp 2009	2641	3183	4415	24019	21.0	0.37 [0.36, 0.39]	0.88 [0.88, 0.89]
Fong 2018	6	5	68	221	25.0	0.08 [0.03, 0.17]	0.98 [0.95, 0.99]
Wuerz 2001	1113	674	1260	5204	28.0	0.47 [0.45, 0.49]	0.89 [0.88, 0.89]
Wuerz 2000	91	53	68	281	32.0	0.57 [0.49, 0.65]	0.84 [0.80, 0.88]
Graff 2014	6497	2132	8463	28377	33.0	0.43 [0.43, 0.44]	0.93 [0.93, 0.93]
Chi 2006	713	747	376	1336	34.0	0.65 [0.63, 0.68]	0.64 [0.62, 0.66]

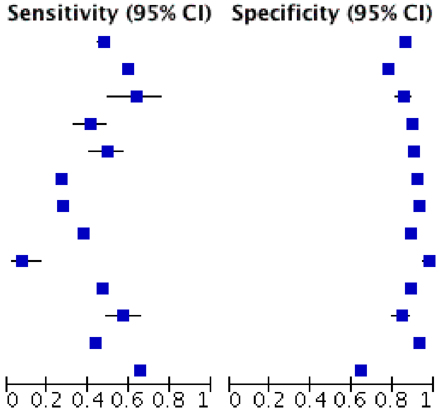

Children

Study	TP	FP	FN	TN	Hospitalization rate	Sensitivity (95% CI)	Specificity (95% CI)
Al-Hindi 2014	65	295	45	2609	4.0	0.59 [0.49, 0.68]	0.90 [0.89, 0.91]
Gouin 2005	8	19	38	472	9.0	0.17 [0.08, 0.31]	0.96 [0.94, 0.98]
Gravel 2009	1937	2746	3124	50722	9.0	0.38 [0.37, 0.40]	0.95 [0.95, 0.95]
Green 2012	33	42	47	658	10.0	0.41 [0.30, 0.53]	0.94 [0.92, 0.96]
Aeimchanbanjong 2017	62	239	62	679	12.0	0.50 [0.41, 0.59]	0.74 [0.71, 0.77]
Aeimchanbanjong 2017	64	165	59	753	12.0	0.52 [0.43, 0.61]	0.82 [0.79, 0.84]
Aeimchanbanjong 2017	70	285	53	633	12.0	0.57 [0.48, 0.66]	0.69 [0.66, 0.72]
Baumann 2005	44	66	28	372	14.0	0.61 [0.49, 0.72]	0.85 [0.81, 0.88]
Chang 2013	3104	6821	6276	26327	23.0	0.33 [0.32, 0.34]	0.79 [0.79, 0.80]
Allon 2017	2631	2330	17726	60922	24.0	0.13 [0.12, 0.13]	0.96 [0.96, 0.96]

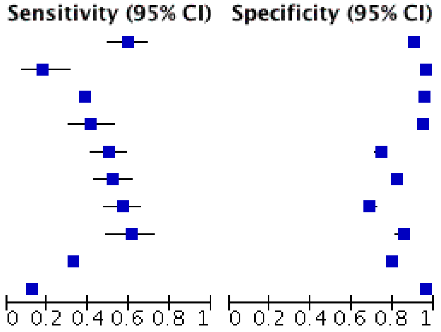

TP = true positive; FP = false positive; FN = false negative; TN = true negative
