# Supplementary material for: Performance of triage systems in emergency care: a systematic review and meta-analysis
Source: BMJ Open. 2019 May 28;9(5):e026471. doi: 10.1136/bmjopen-2018-026471 (PMC6549628; doi:10.1136/bmjopen-2018-026471)
Supplement: Author's manuscript [file bmjopen-2018-026471.draft_revisions.pdf]

BMJ Open

BMJ Open is committed to open peer review. As part of this commitment we make the peer review history of every article we publish publicly available.

When an article is published we post the peer reviewers' comments and the authors' responses online. We also post the versions of the paper that were used during peer review. These are the versions that the peer review comments apply to.

The versions of the paper that follow are the versions that were submitted during the peer review process. They are not the versions of record or the final published versions. They should not be cited or distributed as the published version of this manuscript.

BMJ Open is an open access journal and the full, final, typeset and author-corrected version of record of the manuscript is available on our site with no access controls, subscription charges or pay-per-view fees (http://bmjopen.bmj.com).

If you have any questions on BMJ Open's open peer review process please email info.bmjopen@bmj.com

BMJ Open

THE PERFORMANCE OF TRIAGE SYSTEMS IN EMERGENCY CARE: A SYSTEMATIC REVIEW AND META-ANALYSIS

Journal:	*BMJ Open*
Manuscript ID	bmjopen-2018-026471
Article Type:	Research
Date Submitted by the Author:	24-Sep-2018

Complete List of Authors:	Zachariasse, Joany; Erasmus MC-Sophia Childrens Hospital, Department of Pediatrics<br>van der Hagen, Vera; Erasmus MC-Sophia Childrens Hospital, Department of Pediatrics<br>Seiger, Nienke; Erasmus MC-Sophia Childrens Hospital, Department of Pediatrics<br>Mackway-Jones, Kevin; Manchester Royal Infirmary, Emergency Department<br>van Veen, Mirjam; Groene Hart Ziekenhuis, Pediatrics; Erasmus MC-Sophia Childrens Hospital, Department of Pediatrics<br>Moll, Henriette; Erasmus MC-Sophia Childrens Hospital, Department of Pediatrics

Keywords:	ACCIDENT & EMERGENCY MEDICINE, EPIDEMIOLOGY, Paediatric A&E and ambulatory care < PAEDIATRICS, PUBLIC HEALTH

SCHOLARONE™ Manuscripts

THE PERFORMANCE OF TRIAGE SYSTEMS IN EMERGENCY CARE: A SYSTEMATIC REVIEW AND META-ANALYSIS

Joany M Zachariasse*, Vera van der Hagen*, Nienke Seiger, Kevin C Mackway-Jones, Mirjam van Veen, Henriëtte A Moll

* Both authors contributed equally

Department of Paediatrics, Erasmus MC- Sophia Children's Hospital, University Medical Centre Rotterdam, PO Box 2060, 3000 CB Rotterdam, The Netherlands

Joany M Zachariasse

PhD-student

Department of Paediatrics, Erasmus MC- Sophia Children's Hospital, University Medical Centre Rotterdam, PO Box 2060, 3000 CB Rotterdam, The Netherlands

Vera van der Hagen

MD

Department of Paediatrics, Erasmus MC- Sophia Children's Hospital, University Medical Centre Rotterdam, PO Box 2060, 3000 CB Rotterdam, The Netherlands

Nienke Seiger

Paediatric resident

Emergency Department, Manchester Royal Infirmary, Oxford Road, Manchester M13 9WL, UK

Kevin C Mackway Jones

Professor of Emergency Medicine

Department of Paediatrics, Erasmus MC- Sophia Children's Hospital, University Medical Centre Rotterdam, PO Box 2060, 3000 CB Rotterdam, The Netherlands

Currently: Department of Paediatrics, Groene Hart Ziekenhuis, Postal Code 1098, 2800 BB Gouda, The Netherlands

Mirjam van Veen

Paediatrician

For peer review only - http://bmjopen.bmj.com/site/about/guidelines.xhtml

Department of Paediatrics, Erasmus MC- Sophia Children's Hospital, University Medical Centre

Rotterdam, PO Box 2060, 3000 CB Rotterdam, The Netherlands

Henriëtte A Moll

Professor of Paediatrics

Correspondence to:
Henriëtte A Moll
h.a.moll@erasmusmc.nl

Word count: 3729

Word count abstract: 248

Abbreviations:

CTAS Canadian Triage and Acuity Scale

ED emergency department

ESI Emergency Severity Index

FN false negative

FP false positive

MTS Manchester Triage System

TN true negative

TP true positive

Funding: This research received no specific grant from any funding agency in the public, commercial or not-for-profit sectors

Competing interests: No support from any organisation for the submitted work; KMJ is chair of the Manchester Triage Group; there are no other relationships or activities that could appear to have influenced the submitted work.

Dating sharing: No additional data available

For peer review only - http://bmjopen.bmj.com/site/about/guidelines.xhtml

Abstract

Objective

To assess and compare the performance of triage systems for identifying high and low urgency patients in the emergency department

Design

Systematic review and meta-analysis

Data sources

EMBASE, Medline OvidSP, Cochrane central, Web-of-science and CINAHL databases from 1980 to 2014 with an update in June 2016.

Eligibility criteria

Studies that evaluated an emergency medical triage system, assessed validity using any reference standard as proxy for true patient urgency and were written in English.

Review methods

Reviewers identified studies, extracted data, and assessed the quality of the evidence independently and in duplicate. Raw data were extracted to create 2x2 tables and calculate sensitivity and specificity. ED patient volume and case-mix severity of illness were investigated as determinants of triage systems' performance.

Results

Sixty eligible studies evaluated thirty different triage systems. Comparisons were restricted to the three triage systems that had at least multiple evaluations using the same reference standard (Canadian Triage and Acuity Scale, Emergency Severity Index, and Manchester Triage System). Overall validity to identify high and low urgency patients of each triage system was moderate to good, but performance was highly variable. In a subgroup analysis, no clear association was found between ED patient volume or case-mix severity of illness and triage systems' performance.

Conclusions

Established triage systems show a reasonable validity for the triage of patients at the ED, but performance varies considerably. Important research questions that remain are what determinants influence triage systems' performance and how the performance of existing triage systems can be improved.

For peer review only - http://bmjopen.bmj.com/site/about/guidelines.xhtml

Strengths and limitations of the study

- The present study was based on a comprehensive and systematic search and attempted to include as many relevant studies as possible

- This study is the first synthesis of the available evidence on triage systems as previous reviews have merely described the results of the individual studies .

- The majority of triage systems were evaluated by less than three studies. Even for the three most commonly used triage systems, few evaluations were available due to the variety of reference standards

- Due to the limited number of studies and the heterogeneity of study populations it was difficult to compare results across studies and we could not provide summary estimates.

For peer review only - http://bmjopen.bmj.com/site/about/guidelines.xhtml

Introduction

Overcrowding of emergency departments (EDs) is a universal and ever increasing problem.[1-3] Therefore, most EDs have a triage system in place to facilitate the prioritization of patients. In recent years several formal triage scales have been developed to standardize the approach to triage. These include amongst others the Australian Triage Scale, the Canadian Triage and Acuity Scale (CTAS), the Emergency Severity Index (ESI) and the Manchester Triage System (MTS).[4-7]

It is important to evaluate the performance of triage systems for their ability to accurately distinguish between both high and low urgency patients. The correct classification of high urgency patients is related to patient safety, because misclassification of high urgency patients to a low urgency level causes delay in diagnosis and treatment, potentially leading to morbidity or mortality. The correct classification of low urgency patients increases efficiency of the ED flow and reduces waiting times for the truly high urgency ED visits.

Research regarding the performance of triage systems mainly consists of observational studies in heterogeneous populations using a variety of reference standards. Previous reviews have primarily described the results of these individual studies without combining and interpreting the evidence into overall conclusions.[8-10] A systematic appraisal of the performance of commonly used triage systems can inform clinicians and policy makers about the safety and efficacy of available triage systems and provide insights on which triage system is safe and efficient to use. Moreover it can highlight gaps in current research and propose directions for future studies.

The aim of this systematic review is to provide a comprehensive overview of current evidence on the performance of triage systems. We assessed and compared the performance of the most commonly used triage systems for the prioritization of high- and low urgency patients at the ED, compared to any reference standard that is a proxy of true patient urgency. Furthermore, we aimed to investigate whether patient volume and case-mix at the ED, are determinants of triage systems' performance.

For peer review only - http://bmjopen.bmj.com/site/about/guidelines.xhtml

Methods

Search strategy and selection criteria

Meta-analysis of Observational Studies in Epidemiology (MOOSE) guidelines were followed for the conduct of this study.[11] We conducted a systematic review using a broad search strategy to identify all studies assessing the performance of triage systems in emergency care when compared to any reference standard that is a proxy of true patient urgency. A search strategy was developed by a health sciences librarian and included medical subject headings (MeSH) and text words related to triage, emergency care and validity (Appendix 1). We searched EMBASE, Medline OvidSP, Cochrane central, Web-of-science and CINAHL databases from 1980 to 2014 with an update in June 2016.

Two reviewers (MV and NS or JZ and VH) independently assessed eligibility for inclusion. Disagreements in article selection were resolved through discussion. Studies were selected if they evaluated an emergency medical triage system, assessed validity using a reference standard and were written in English. Letters, abstracts, reviews, conference proceedings, and case reports were excluded. We did not include studies on trauma triage systems, early warning scores, or studies conducted in specific subpopulations. Moreover, we excluded studies with less than 100 patients and studies based on case scenarios because these studies have of a high risk of bias, and studies conducted in low or lower-income economies[12] because these results are poorly generalizable to most emergency departments in western countries.

Data extraction

Two reviewers (JZ and VH) independently extracted data from each of the included studies. A single study could consist of multiple "triage evaluations", defined as the analysis of a single triage system in a single age group. Predefined age groups were 1) children; 2) adults or a combination of age groups; and 3) elderly. For each of the triage evaluations, the reviewers extracted the total number of included patients, the number of patients in each of the urgency categories of the triage system, the type of reference standard used, and the number of patients with a positive reference standard in each urgency category. If studies were based on overlapping data, we used the results from the most recent publication. For descriptive purposes we also collected data on study design and methods, patient demographics, and characteristics of the settings in which the study was performed.

For peer review only - http://bmjopen.bmj.com/site/about/guidelines.xhtml

Quality assessment

Two reviewers independently assessed quality of the selected articles using the QUADAS-2 (Quality Assessment of studies of Diagnostic Accuracy included in Systematic Reviews) checklist.[13] The QUADAS-2 evaluates 4 domains: patient selection, index test, reference standard, and flow and timing. Each domain is assessed in terms of risk of bias, and the first 3 domains also in terms of applicability. Because triage systems have some specific features as compared to other diagnostic tests, we adjusted the "reference standard domain" to make it applicable to our research question. We did not appraise whether the reference standard was interpreted without knowledge of the result of the index test, because this is unlikely when triage is applied in routine care. We did evaluate, however, whether data on the outcome was collected blinded to the result of the index test. Moreover, we did not judge the applicability of the reference standard because there is no consensus on this topic.[14] Therefore, we included all studies with reference standard that were a proxy for patient urgency. Any disagreements between reviewers were resolved in a consensus meeting.

Data analysis

We used descriptive analyses to provide an overview of the available evidence on triage systems. Further analyses were restricted to triage systems that underwent at least 3 evaluations with the same reference standard. The primary outcome of our review was sensitivity and specificity of each of the triage systems for the identification of high and low urgency patients. Because there is no golden standard to determine "true" patient urgency, we a-priori selected 3 reference standards as proxy for patient urgency. We considered mortality at the ED and ICU admission after the ED visits as reference standard for high urgency, and discharge after the ED visit (i.e. patients not admitted to hospital) as reference standard for low urgency. Although these measures are not perfect, they approximate the desired outcome: most patients that die at the ED or require ICU admission are of high urgency, while most patients that are discharged after the ED visit are not. Moreover, these measures are suitable for the analyses of large datasets and commonly reported in research on triage systems.[14] In addition, we considered any other reference standard with sufficient evaluations in the same triage system.

For peer review only - http://bmjopen.bmj.com/site/about/guidelines.xhtml

We calculated two by two tables of triage system against the reference standard for each individual study. Because triage systems are ordinal scales, we dichotomized the urgency categories into a high urgency and low urgency group. High urgency was defined as triage urgency level 1 (3-level systems) or triage urgency level 1 and 2 (4-level and 5-level systems).

We calculated sensitivities and specificities, and presented the results as forest plots. For clinical practice and for benchmarking purposes, it may be interesting to know the proportion of patients with a positive reference standard in each of the triage urgency categories. Therefore, we additionally calculated the proportion of patients with a positive reference standard per urgency category, and displayed the results in a bar chart to enable comparison between studies and between triage systems.

We hypothesized that ED patient volume and case-mix severity of illness were determinants of triage systems' performance. Therefore we decided that if a sufficient number of studies were identified, we would investigate the effect of these determinants on triage systems' performance using subgroup analyses. We considered annual ED census as a marker of patient volume and the percentage of hospitalized patients as a marker of case-mix severity of illness.

Computations were carried out with SPSS Statistics version 21.0 and figures were created using Review Manager 5.3 or R version 3.2.0.[15-17]

Patient and Public Involvement

Patients and public were not involved in this study.

For peer review only

For peer review only - http://bmjopen.bmj.com/site/about/guidelines.xhtml

Results

A total of 11,050 papers were identified in the electronic search, of which sixty were included in the final selection (figure 1).

The majority of studies were conducted in tertiary or university hospitals (n=43; 72%) and conducted in Europe/Central Asia or North America (n=44; 73%). Forty-three (72%) were single-center studies. A complete overview of the selected studies is presented in appendix 2.

Forty studies (67%) had a high risk of bias in at least one domain, and fourteen studies (35%) had a high risk of bias in two or more domains (figure 2 and appendix 3). The most common causes of concern were application of multiple triage systems for the same patient by the same nurse or by multiple nurses without blinding, retrospective retrieval of reference standard information without blinding for the triage outcome, and substantial amounts of missing data. In ten studies (17%) there were concerns regarding applicability.

Triage systems

In total, 77 individual triage evaluations were reported: 25 (33%) in children, 49 (64%) in adults, a combination of age groups or an unspecified population, and 3 (4%) in elderly. A total of 30 different triage systems were evaluated. The most commonly evaluated triage systems were the Emergency Severity Index (ESI) (n=19), the Manchester Triage System (MTS) (n=14), and the Canadian Triage and Acuity Scale (CTAS) (n=12). Other triage systems included the Taiwan Triage System (n=4), Netherlands Triage System (n=2), and Soterion Rapid Triage System (n=2). For 24 triage systems only one evaluation was published. These included 9 local or informally structured triage systems. The median sample size was 3,014 in children (range: 510 to 550,940), 1,851 in adults (range: 100 to 316,622), and 929 in elderly (range: 773 to 1,903).

Reference standards

A variety of reference standards were used and the majority of studies reported multiple reference standards (Appendix 4). 12 studies used mortality at the ED as a reference standard, 12 studies ICU admission and 41 studies hospital admission. Other commonly reported reference standards were length of stay at the ED (26 studies), resource use at the ED according to the ESI criteria (13 studies),

For peer review only - http://bmjopen.bmj.com/site/about/guidelines.xhtml

expert opinion (9 studies) and costs (8 studies). Because definitions of time to mortality, resource use and expert opinion were not consistent across studies, these results could not be compared. Moreover, length of stay at the ED and costs are outcome measures that are strongly dependent on ED characteristics, and could therefore not be used to make a comparison between studies.

The most commonly evaluated triage systems

Because we cannot interpret analyses of triage systems evaluated with different reference standards, we will further restrict our analyses to the triage systems with at least three evaluations using the same reference standard. This final selection includes studies evaluating the ESI, CTAS and MTS. Characteristics of these triage systems and a summary of the available evidence are presented in table 1. The ESI, CTAS and MTS were all evaluated in settings with a different patient volume as indicated by annual hospital census, and a different case-mix as indicated by percentage of hospitalization. For each of these triage systems, the majority of studies had risk of bias in at least one domain.

Accuracy of triage systems to identify high urgent patients

Mortality at the ED was reported in 7 evaluations of our final sample: 5 evaluations in adults, and 2 in children. Because of this low number of studies and the very low reported mortality rates (on average 0.2% in adults and <0.01% in children) it was not possible to perform comparative analyses.

ICU admission was reported in 5 evaluations in adults (2 ESI, 3 MTS) and 4 in children (3 CTAS, 1 MTS). Overall, sensitivity for ICU admission was moderate to good, ranging from 0.58 (95%CI 0.48 to 0.68) to 0.88 (95%CI 0.70 to 0.96) in adults and 0.71 (95%CI 0.66 to 0.77) to 0.93 (95%CI 0.89 to 0.95) in children. A clear difference in performance between the triage systems was not visible (figure.3).

Regardless of the triage system used, most of the ICU admitted patients were allocated to one of the two highest triage categories (Appendix 5). The exact proportion of ICU admitted patients in each triage category was highly variable, even within studies evaluating the same triage system. For example, the proportion of ICU admitted adults in MTS category 1 ranged from 21% to 79%. The number of studies, however, was too small to assess whether this variability was present in all triage systems and whether this could be explained by study or setting related factors.

For peer review only - http://bmjopen.bmj.com/site/about/guidelines.xhtml

Accuracy to identify low urgent patients

Hospital admission or discharge after the ED visit was reported as a reference standard in 13 evaluations in adults and 11 in children. Overall, specificity of the triage systems to accurately classify patients discharged home as low urgent ranged from 0.64 (95%CI 0.62 to 0.66) to 0.93 (95%CI 0.93 to 0.93) in adults and 0.70 (95%CI 0.67 to 0.73) to 0.96 (95%CI 0.94 to 0.98) in children (Figure 4). Again, sensitivities and specificities were highly variable within each of the triage systems. None of the triage systems showed a marked better specificity compared to the others.

The proportion of patients discharged after the ED visit increased from the higher to the lower urgency categories in all triage systems (Appendix 5). Again, there was a large variability within triage systems and substantial overlap between triage systems. In adults, the MTS seemed to have a higher variability compared to the other triage systems, but in children variability was greater for the CTAS.

The only additional reference standard with sufficient evaluations was resource use according to the ESI criteria (Appendix 6).

Direct comparison of triage systems

A total of 13 studies directly compared two or more triage systems. Only two studies comparing the ESI with the MTS were part of the final selection and provided data for construction of a contingency table. A single-center study by Storm-Versloot et al. directly compared the ESI with the MTS and with an informally structured triage system in the same patients.[18] This study found comparable sensitivities and specificities for hospital admission: specificity of the ESI was 0.89 (95%CI 0.87 to 0.91) and specificity of the MTS was 0.90 (95%CI 0.88 to 0.92). Beside hospital admission, the study also assessed validity of the triage systems using an expert panel and reported percentages of misclassification to a too low urgency level, so-called “undertriage”. Compared with the expert judgments, the ESI undertriaged 13.5% of patients while the MTS undertriaged 8.3% at the cost of a larger proportion of “overtriage”. The study concluded that all triage systems appeared equally valid. This study, however, has a high risk of bias in the index test domain because triage was performed by the same nurse using the different triage systems sequentially. It is plausible that this lack of blinding has reduced the differences between the triage systems. A second study by van der Wulp et al. compared triage in 2 teaching hospitals using the ESI with 2 teaching hospitals using the MTS.[19] Again, sensitivities and specificities for hospital admission were similar, with 0.93 (95%CI 0.93 to 0.93) specificity of the ESI, and 0.88 (95%CI 0.88 to 0.89)

For peer review only - http://bmjopen.bmj.com/site/about/guidelines.xhtml

specificity of the MTS. The study also assessed performance of the triage systems to predict mortality. The ESI classified 26 of 28 patients, and the MTS 28 of 29 patients that died at the ED as high urgent.

Determinants of triage systems' performance

The number of studies per triage system was rather small to perform subgroup analyses based on annual census or percentage hospitalization. As an explorative analysis, we ordered all selected studies that used hospital admission as a reference standard based on annual census, and percentage hospitalization (Appendix 7). There was no clear association between patient volume or case-mix and triage systems' sensitivity and specificity. A lower specificity for hospitals with the largest annual census and highest percentage hospitalization could not be ruled out, but requires a larger number of studies.

For peer review only - http://bmjopen.bmj.com/site/about/guidelines.xhtml

Discussion

In a systematic review of 60 observational studies evaluating triage systems, we found that numerous different triage systems are being used but that many lack a rigorous evaluation. The most commonly used and evaluated triage systems, CTAS, ESI and MTS, show a moderate to good validity to identify high and low urgent patients. Their performance, however, is highly variable and differences in study design, study populations and reference standards make a comparison of the available evidence difficult. Although based on a limited number of studies, no clear association between patient volume and case-mix severity of illness could be found.

Strengths and weaknesses

This is the first study that evaluates the performance of triage systems in a meta-analysis. Previous reviews have merely described the results of the individual studies without synthesizing the evidence. Moreover, none of the published reviews looked at other factors that determine triage systems' performance, such as ED characteristics to compare evidence from different studies.[8-10, 20-23]

Our review is based on a comprehensive search developed with a research librarian, includes duplicate assessment of eligibility and risk of bias, and duplicate data abstraction. Furthermore, the research question is based on a relevant and practical clinical issue. Triage systems are used worldwide to prioritize patients in the ED, but robust evidence on their performance is lacking.

The results of this review, however, should be interpreted taking into consideration the limitations of the underlying evidence. We included 60 studies in our review, but the majority of the 30 triage systems were evaluated by only one study. Therefore, we could only evaluate three most frequent used triage systems: CTAS, ESI and MTS. Even for these commonly used triage systems, few evaluations were available due to the variety of reference standards. Although the triage evaluations included the whole age spectrum, studies targeted at elderly patients were scarce. It is important to evaluate triage systems' performance separately for the most vulnerable populations at the ED, specifically children and elderly. In these patient groups the spectrum of disease, the presence of non-specific signs and symptoms and progression of disease course differs from that in adult patients.

We are not aware of any (randomized) controlled trial that investigates the effect of triage on patient outcome. Therefore we conducted a systematic review of observational studies. Comparing

For peer review only - http://bmjopen.bmj.com/site/about/guidelines.xhtml

observational studies is challenging because the effect of a triage system cannot be assessed independently of its context. Likely, other factors such as ED and hospital characteristics or local practices and training have influenced the results of the included studies. We chose to display the results of different studies in a forest plot. Because of the limited number of studies and heterogeneity of the study populations, it is difficult to compare the results from different triage systems and these plots should be interpreted with caution.

We aimed to explore heterogeneity between studies and more specifically the effect of differences in patient load and case-mix severity of illness. Unfortunately, due to the small number of studies per triage systems, we could not draw strong conclusions about the relation between these factors and triage systems' performance. There are more potential factors that could affect triage systems' validity, such as the local infrastructure, the experience and training of the triage nurse, the presence of a computerized triage application or variations in disease epidemiology. Moreover, since most included studies had risk of bias in at least one domain, we cannot rule out that study design and methodological quality have lead to heterogeneity of the results as well.

We predefined mortality at the ED and ICU admission as reference standards for high patient urgency and discharge home as a reference standard for low patient urgency. Due to the relatively low number of studies reporting mortality and the low mortality rate of patients in the ED, we could not use it as a reference standard. ICU and hospital admission are feasible reference standards for large study populations, and theoretically, criteria for ICU and hospital admission should be reasonably comparable between settings. It is possible, however, that ED and hospital characteristics and local practices result in differences in the decision to admit a patient between EDs.

Implications and future research

Our review identified 77 triage systems for which at least one evaluation was published. Probably, there are more triage systems in use, which are not formally evaluated. There are several advantages, not addressed in this review, of using an established triage system over a local triage scale. Beside that performance of the most commonly used triage systems is known, they have a formal governance structure and undergo regular updates. Moreover, there are standardized implementation guidelines and training programs available.[24-26] The CTAS, ESI and MTS all show a reasonable performance for triage

For peer review only - http://bmjopen.bmj.com/site/about/guidelines.xhtml

at the ED. Our results do not suggest that one of the established triage systems should be preferred over the other.

Our review indicates that large variation of performance exists even in studies assessing the same triage system. This suggests that other factors influence triage systems' performance. Consequently, generalizability of individual studies evaluating a triage system is low and a study on triage validity in one setting may not apply to a setting with different characteristics or in a different health care system. Our review demonstrates that the majority of studies evaluating triage systems were conducted in a single center. Furthermore, most multicenter studies provided only pooled results. Yet, multicenter studies using similar study designs and reference standard definitions are needed to evaluate the range of triage systems' performance in different settings. More importantly, these studies can provide valuable insights in determinants of triage systems' performance and areas of improvement.

The extensive use of triage systems in clinical practice contrasts with the limited number of studies evaluating their performance. Triage systems were typically developed based on expert opinion and implemented out of clinical necessity. They are mostly used in their country or region of origin: the MTS is widely used in the United Kingdom and Europe, the ESI in the United States, and the CTAS in Canada and in French-speaking countries.[27] Since most EDs already have experience with a certain triage system and some triage systems are recommended by national guidelines it could be worthwhile shifting away the focus on triage systems' performance towards improvement of the established triage systems. Our review suggests that there is room for improvement of all triage systems regarding both the correct identification of high urgent and low urgent patients.

The most commonly used triage systems, CTAS, ESI and MTS, have a reasonable validity for the triage of patients at the ED. Important research questions that remain are what determinants influence a triage systems' performance and how the performance of existing triage systems can be improved.

Acknowledgements

We thank Wichor Bramer, biomedical information specialist in Erasmus University Medical Centre, Rotterdam, for his help with developing the search strategy

For peer review only - http://bmjopen.bmj.com/site/about/guidelines.xhtml

Authors' contributions

JMZ and VvdH contributed equally to this work. All authors substantially contributed to the conception and design of the study and interpretation of the findings. JMZ, KMJ and HAM conceived the study idea. JMZ, VvdH, NS, MvV conducted the systematic review including screening of abstracts and full text and risk of bias assessment. JMZ and VvdH extracted data, performed the analyses and wrote the first draft of the manuscript. All authors revised it critically for important intellectual content and gave their approval of the final version. All authors had full access to all of the data (including statistical reports and tables) in the study and can take responsibility for the integrity of the data and the accuracy of the data analysis. HAM is guarantor.

For peer review only

For peer review only - http://bmjopen.bmj.com/site/about/guidelines.xhtml

References

1. Blunt I, Edwards N, Merry L. What's behind the A&E 'crisis'? *Briefing*: Nuffield Trust; 2015.
2. Australasian College for Emergency Medicine. Access Block and Overcrowding in Emergency departments. 2004.
3. Institute of Medicine. Hospital-based emergency care: at the breaking point. 2013.
4. Mackway-Jones K, Marsden J, Windle J. *Emergency triage*. London: BMJ; 2006.
5. Warren DW, Jarvis A, LeBlanc L, et al. Revisions to the Canadian Triage and Acuity Scale paediatric guidelines (PaedCTAS). *Cjem.* 2008;10:224-243.
6. Gilboy NT, P.; Travers, D.A.; Rosenau, A.M.; Eitel, D.R. *Emergency Severity Index, Version 4: Implementation Handbook*. Rockville, MD: Agency for Healthcare Research and Quality; 2005.
7. Australian College for Emergency Medicine. Guidelines on the implementation of the australasian triage scale in emergency departments. http://www.acem.org.au/media/policies_and_guidelines/G24_Implementation__ATS.pdf.
8. Christ M, Grossmann F, Winter D, Bingisser R, Platz E. Modern triage in the emergency department. *Dtsch Arztebl.* 2010;107:892-898.
9. Farrohknia N, Castren M, Ehrenberg A, et al. Emergency department triage scales and their components: a systematic review of the scientific evidence. *Scand J Trauma Resusc Emerg Med.* 2011;19:42.
10. Parenti N, Ferrara L, Bacchi Reggiani ML, Sangiorgi D, Lenzi T. Reliability and validity of two four-level emergency triage systems. *Eur J Emerg Med.* 2009;16:115-120.
11. Stroup DF, Berlin JA, Morton SC, et al. Meta-analysis of observational studies in epidemiology: a proposal for reporting. Meta-analysis Of Observational Studies in Epidemiology (MOOSE) group. *Jama.* 2000;283:2008-2012.
12. World Bank Group. World Bank Country and Lending Groups. https://datahelpdesk.worldbank.org/knowledgebase/articles/906519-world-bank-country-and-lending-groups. Accessed October 5th, 2017.
13. Whiting PF, Rutjes AW, Westwood ME, et al. QUADAS-2: a revised tool for the quality assessment of diagnostic accuracy studies. *Ann Intern Med.* 2011;155:529-536.
14. Kuriyama A, Urushidani S, Nakayama T. Five-level emergency triage systems: variation in assessment of validity. *Emerg Med J.* 2017.
15. R Development Core Team. R: A language and environment for statistical computing. Vienna, Austria: R Foundation for Statistical Computing; 2008.
16. IBM Corp. IBM SPSS Statistics for Windows. 21 ed. Armonk, NY 2011.
17. Review Manager (RevMan). 5.3 ed. Copenhagen: The Nordic Cochrane Centre, The Cochrane Collaboration; 2014.
18. Storm-Versloot MN, Ubbink DT, Kappelhof J, Luitse JSK. Comparison of an informally structured triage system, the emergency severity index, and the manchester triage system to distinguish patient priority in the emergency department. *Acad Emerg Med.* 2011;18:822-829.
19. van der Wulp I, Schrijvers AJP, van Stel HF. Predicting admission and mortality with the Emergency Severity Index and the Manchester Triage System: a retrospective observational study. *Emergency Medicine Journal.* 2009;26:506-509.
20. Azeredo TR, Guedes HM, Rebelo de Almeida RA, Chianca TC, Martins JC. Efficacy of the Manchester Triage System: a systematic review. *Int Emerg Nurs.* 2015;23:47-52.
21. Souza CC, Araujo FA, Chianca TC. [Scientific Literature on the Reliability and Validity of the Manchester Triage System (MTS) Protocol: A Integrative Literature Review]

Producao cientifica sobre a validade e confiabilidade do Protocolo de Manchester: revisao integrativa da literature. *Rev Esc Enferm USP.* 2015;49:144-151.

For peer review only - http://bmjopen.bmj.com/site/about/guidelines.xhtml

22. van Veen M, Moll HA. Reliability and validity of triage systems in paediatric emergency care. *Scand J Trauma Resusc Emerg Med.* 2009;17:38.

23. de Magalhaes-Barbosa MC, Robaina JR, Prata-Barbosa A, Lopes CS. Validity of triage systems for paediatric emergency care: a systematic review. *Emerg Med J.* 2017.

24. www.esitriage.com. Accessed October 5th, 2017. .

25. http://caep.ca/resources/ctas. Accessed October 5th, 2017.

26. www.triagenet.net. Accessed October 5th, 2017. Vol 2017.

27. Moll HA. Challenges in the validation of triage systems at emergency departments. *J Clin Epidemiol.* 2010;63:384-388.

For peer review only

For peer review only - http://bmjopen.bmj.com/site/about/guidelines.xhtml

Tables and Figure captions

Table 1. Evidence summary of the most commonly used triage systems

	**Canadian Triage And Acuity Scale (CTAS)**	**Emergency Severity Index (ESI)**	**Manchester Triage System (MTS)**
Triage system characteristics			
Description	List, based on presenting signs and symptoms	Flowchart, based on physical signs and expected resource use	Multiple flowcharts, based on presentational signs and symptoms
Number of levels	5	5	5
Classification and waiting time	Level I, Immediate<br>Level II, 15 min<br>Level III, 30 min<br>Level IV, 60 min<br>Level V, 2 hrs	Immediate<br>Emergent, 14 min<br>Urgent, 60 min<br>Semi-urgent, 2 hrs<br>Non-urgent, 24 hrs	Immediate<br>Very urgent, 10 min<br>Urgent, 60 min<br>Standard, 2 hrs<br>Non-urgent, 4 hrs
Quantity of evidence			
Total number of evaluations	12	19	14
Evaluations in children	8	3	6
Evaluations in elderly	1	2	0
Diversity of evidence (range)			
Number of hospitals per study	1-12	1-7	1-4
Inclusions per study	481-550,940	180-37,974	872-31,622
Hospital census	25,000-75,000	13,838-90,000	7,000-190,000
Hospitalization rate	8%-47%	10%-62%	5-33%
Risk of bias			
High risk of bias in at least one domain	50%	80%	62%
High risk of bias in >1 domain	8%	20%	8%

For peer review only

For peer review only - http://bmjopen.bmj.com/site/about/guidelines.xhtml

Figure 1. Flow diagram of study selection process

Figure 2. Risk of bias of included studies

Figure 3. Sensitivity and specificity of triage systems for identifying high urgency patients as defined by ICU admission

CTAS = Canadian Triage and Acuity Scale; TP = true positive; FP = false positive; FN = false negative; TN = true negative; ESI = Emergency Severity Index; MTS = Manchester Triage System

Figure 4. Sensitivity and specificity of triage systems for identifying low urgency patients as defined by discharge home after the ED visit

CTAS = Canadian Triage and Acuity Scale; TP = true positive; FP = false positive; FN = false negative; TN = true negative; ESI = Emergency Severity Index; MTS = Manchester Triage System

For peer review only

For peer review only - http://bmjopen.bmj.com/site/about/guidelines.xhtml

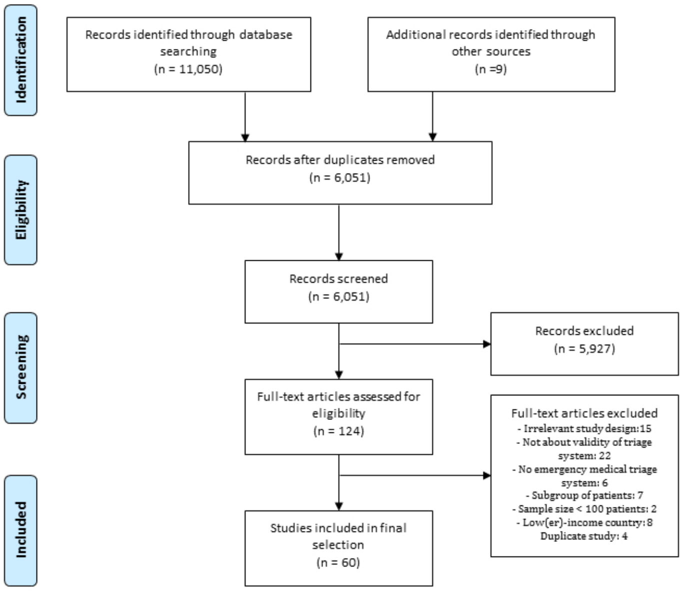

Figure 1. Flow diagram of study selection process

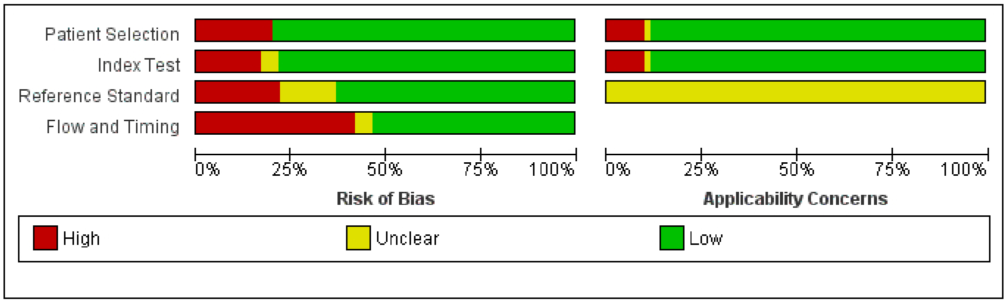

Figure 2. Risk of bias of included studies

239x70mm (72 x 72 DPI)

CTAS

Study	TP	FP	FN	TN	Age group	Sensitivity (95% CI)	Specificity (95% CI)
Gravel 2009	268	4415	21	53825	Children	0.93 [0.89, 0.95]	0.92 [0.92, 0.93]
Gravel 2013	1646	64284	434	482987	Children	0.79 [0.77, 0.81]	0.88 [0.88, 0.88]
Allon 2017	264	4697	128	78520	Children	0.67 [0.62, 0.72]	0.94 [0.94, 0.95]

ESI

Study	TP	FP	FN	TN	Age group	Sensitivity (95% CI)	Specificity (95% CI)
Tanabe 2004	22	155	3	223	Adults / Unspecified	0.88 [0.69, 0.97]	0.59 [0.54, 0.64]
Yuksen 2016	15	91	3	411	Adults / Unspecified	0.83 [0.59, 0.96]	0.82 [0.78, 0.85]

MTS

Study	TP	FP	FN	TN	Age group	Sensitivity (95% CI)	Specificity (95% CI)
Zachariasse 2016	208	11323	83	54447	Children	0.71 [0.66, 0.77]	0.83 [0.82, 0.83]
Graff 2014	1888	6741	422	36418	Adults / Unspecified	0.82 [0.80, 0.83]	0.84 [0.84, 0.85]
Steiner 2016	54	588	39	1726	Adults / Unspecified	0.58 [0.47, 0.68]	0.75 [0.73, 0.76]
Zachariasse 2017	836	34642	166	186925	Adults / Unspecified	0.83 [0.81, 0.86]	0.84 [0.84, 0.85]

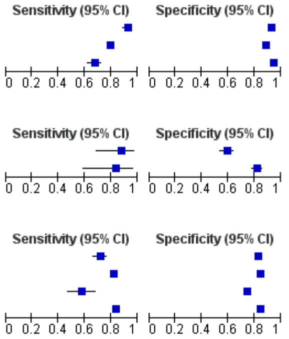

Figure 3. Sensitivity and specificity of triage systems for identifying high urgency patients as defined by ICU admission

CTAS = Canadian Triage and Acuity Scale; TP = true positive; FP = false positive; FN = false negative; TN = true negative; ESI = Emergency Severity Index; MTS = Manchester Triage System

320x136mm (72 x 72 DPI)

CTAS

Study	TP	FP	FN	TN	Age group	Sensitivity (95% CI)	Specificity (95% CI)
Gouin 2005	8	19	38	472	Children	0.17 [0.08, 0.31]	0.96 [0.94, 0.98]
Gravel 2009	1937	2746	3124	50722	Children	0.38 [0.37, 0.40]	0.95 [0.95, 0.95]
Gravel 2012	30	69	69	1296	Children	0.30 [0.21, 0.40]	0.95 [0.94, 0.96]
Chang 2013	3104	6821	6276	26327	Children	0.33 [0.32, 0.34]	0.79 [0.79, 0.80]
Gravel 2013	20876	45054	25093	458328	Children	0.45 [0.45, 0.46]	0.91 [0.91, 0.91]
Al-Hindi 2014	65	295	45	2609	Children	0.59 [0.49, 0.68]	0.90 [0.89, 0.91]
Allon 2017	2631	2330	17726	60922	Children	0.13 [0.12, 0.13]	0.96 [0.96, 0.96]
Dong 2007	1519	1947	4189	21869	Adults / Unspecified	0.27 [0.25, 0.28]	0.92 [0.91, 0.92]
Ng 2010	271	244	350	986	Adults / Unspecified	0.44 [0.40, 0.48]	0.80 [0.78, 0.82]

ESI

Study	TP	FP	FN	TN	Age group	Sensitivity (95% CI)	Specificity (95% CI)
Baumann 2005	44	66	28	372	Children	0.61 [0.49, 0.72]	0.85 [0.81, 0.88]
Travers 2009	276	168	50	679	Children	0.85 [0.80, 0.88]	0.80 [0.77, 0.83]
Green 2012	33	42	47	658	Children	0.41 [0.30, 0.53]	0.94 [0.92, 0.96]
Wuerz 2000	91	53	68	281	Adults / Unspecified	0.57 [0.49, 0.65]	0.84 [0.80, 0.88]
Wuerz 2001	1113	674	1260	5204	Adults / Unspecified	0.47 [0.45, 0.49]	0.89 [0.88, 0.89]
Tanabe 2004	129	48	75	151	Adults / Unspecified	0.63 [0.56, 0.70]	0.76 [0.69, 0.82]
Chi 2006	713	747	376	1336	Adults / Unspecified	0.65 [0.63, 0.68]	0.64 [0.62, 0.66]
Van der Wulp 2009	2259	2146	5911	27658	Adults / Unspecified	0.28 [0.27, 0.29]	0.93 [0.93, 0.93]
Storm-Versloot 2011	66	76	96	638	Adults / Unspecified	0.41 [0.33, 0.49]	0.89 [0.87, 0.92]
Yuksen 2016	38	68	22	392	Adults / Unspecified	0.63 [0.50, 0.75]	0.85 [0.82, 0.88]

MTS

Study	TP	FP	FN	TN	Age group	Sensitivity (95% CI)	Specificity (95% CI)
Roukema 2006	147	256	62	600	Children	0.70 [0.64, 0.76]	0.70 [0.67, 0.73]
Seiger 2014	2992	7410	3903	46430	Children	0.43 [0.42, 0.45]	0.86 [0.86, 0.87]
Van der Wulp 2009	2641	3183	4415	24019	Adults / Unspecified	0.37 [0.36, 0.39]	0.88 [0.88, 0.89]
Martins 2009	18265	63803	12464	222090	Adults / Unspecified	0.59 [0.59, 0.60]	0.78 [0.78, 0.78]
Storm-Versloot 2011	73	72	76	651	Adults / Unspecified	0.49 [0.41, 0.57]	0.90 [0.88, 0.92]
Santos 2013	485	3080	535	19515	Adults / Unspecified	0.48 [0.44, 0.51]	0.86 [0.86, 0.87]
Graff 2014	6497	2132	8463	28377	Adults / Unspecified	0.43 [0.43, 0.44]	0.93 [0.93, 0.93]

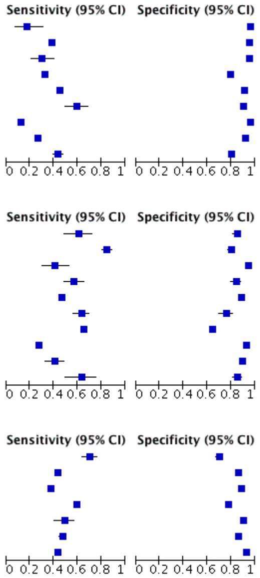

Figure 4. Sensitivity and specificity of triage systems for identifying low urgency patients as defined by discharge home after the ED visit
CTAS = Canadian Triage and Acuity Scale; TP = true positive; FP = false positive; FN = false negative; TN = true negative; ESI = Emergency Severity Index; MTS = Manchester Triage System

354x232mm (72 x 72 DPI)

Appendix 1. Search strategy

Embase
(triage* OR ((acuity OR severit* OR priorit* OR critical*) NEAR/3 (scale* OR level* OR index* OR score* OR measure* OR assessment*))):ab,ti AND ('emergency ward'/de OR 'emergency medicine'/de OR 'emergency care'/exp OR (((emergen* OR acute) NEAR/3 (department* OR ward* OR unit* OR room* OR care))):ab,ti) AND (reliability/exp OR reproducibility/de OR 'validation process'/de OR 'validation study'/de OR accuracy/de OR 'sensitivity and specificity'/exp OR 'diagnostic accuracy'/de OR evaluation/de OR validity/exp OR (reliab* OR reproducib* OR validation OR validaty OR consisten* OR variabilit* OR accura* OR 'intra observer' OR intraobserver OR sensitivity OR specificity):ab,ti OR evaluat*:ti) AND [english]/lim

Medline Ovid
(Triage/ OR (triage* OR ((acuity OR severit* OR priorit* OR critical*) ADJ3 (scale* OR level* OR index* OR score* OR measure* OR assessment*))).ab,ti.) AND (exp "Emergency Service, Hospital"/ OR "emergency medicine"/ OR "emergency care"/ OR (((emergen* OR acute) ADJ3 (department* OR ward* OR unit* OR room* OR care))).ab,ti.) AND (Reproducibility of Results/ OR Validation Studies.pt. OR exp "sensitivity and specificity"/ OR Evaluation Studies.pt. OR (reliab* OR reproducib* OR validation OR validaty OR consisten* OR variabilit* OR accura* OR "intra observer" OR intraobserver OR sensitivity OR specificity).ab,ti. OR evaluat*.ti.) AND english.la.

Cochrane central
(triage* OR ((acuity OR severit* OR priorit* OR critical*) NEAR/3 (scale* OR level* OR index* OR score* OR measure* OR assessment*))):ab,ti AND ((((emergen* OR acute) NEAR/3 (department* OR ward* OR unit* OR room* OR care))):ab,ti) AND ((reliab* OR reproducib* OR validation OR validaty OR consisten* OR variabilit* OR accura* OR 'intra observer' OR intraobserver OR sensitivity OR specificity):ab,ti OR evaluat*:ti)

Web-of-science
TS=(triage* OR ((acuity OR severit* OR priorit* OR critical*) NEAR/3 (scale* OR level* OR index* OR score* OR measure* OR assessment*))) AND TS=((((emergen* OR acute) NEAR/3 (department* OR ward* OR unit* OR room* OR care)))) AND (TS=(reliab* OR reproducib* OR validation OR validaty OR consisten* OR variabilit* OR accura* OR "intra observer" OR intraobserver OR sensitivity OR specificity) OR TI=(evaluat*)) AND LA=(English)

CINAHL
(MH Triage+ OR (triage* OR ((acuity OR severit* OR priorit* OR critical*) N3 (scale* OR level* OR index* OR score* OR measure* OR assessment*)))) AND (MH "Emergency Service"+ OR MH "emergency medicine"+ OR (((emergen* OR acute) N3 (department* OR ward* OR unit* OR room* OR care)))) AND (MH Reproducibility of Results+ OR MH Validity+ OR MH "sensitivity and specificity"+ OR MH Evaluation+ OR (reliab* OR reproducib* OR validation OR validaty OR consisten* OR variabilit* OR accura* OR "intra observer" OR intraobserver OR sensitivity OR specificity) OR TI (evaluat*))

Google Scholar
triage emergency|emergencies
reliability|reproducibility|validation|validity|consistency|accuracy|sensitivity|specificity|interobserver |intraobserver

For peer review only

Appendix 2. Selected studies

Table 2.1 Characteristics of included studies

Study	Setting	Annual census	Hospitalization rate	Study population	Triage System	Reference standard (data available)
Al-Hindi, 2014[1]	Single tertiary maternity and children's hospital, Saudi Arabia	155,000	4%	3014 children	Ped-CTAS	- Hospital admission<br>- Resource use, other<br>- Waiting time to physician
Allon, 2017[2]	Single tertiary children's hospital, Israel	-	24%	83,609 children	Ped-CTAS	- Hospital admission<br>- ICU admission<br>- Length of stay at the ED<br>- Left without being seen
Baumann, 2005[3]	Single academic hospital, USA	53,000	14%	510 children	ESI v.3	- Hospital admission<br>- Resource use based on ESI<br>- Length of stay at the ED<br>- Mortality at the ED<br>- Mortality after 1 year<br>- Left without being seen
Baumann, 2006[4]	Single academic hospital, USA	53,000	43%	929 elderly	ESI v.3	- Hospital admission<br>- ICU admission<br>- Resource use based on ESI<br>- Length of stay at the ED<br>- Mortality at the ED<br>- Mortality after 1 year<br>- Left without being seen
Brillman, 1996[5]	Single academic hospital, USA	65,000	6%	5106 patients	Automated Military Outpatient System	- Hospital admission<br>- Expert opinion
Bruijns, 2008[6]	Community hospital, South Africa	54,000	40%	798 adults	Cape Triage Score	- Hospital admission<br>- Mortality at the ED
Chang, 2012[7]	Single academic	-	23%	42,346	CTAS and	- Hospital admission

For peer review only

	hospital,<br>Taiwan			children	Ped-TTS	- Costs
Chi, 2006[8]	Single academic hospital,<br>Taiwan	58,000	34%	3172 adults	ESI v.2 and TTS	- Hospital admission<br>- Length of stay at the ED
Dong, 2007[9]	Single tertiary hospital,<br>Canada	67,000	19%	29,524 adults	CTAS	- Hospital admission<br>- Resource use, other<br>- Length of stay at the ED<br>- Mortality at the ED<br>- Costs
Dugas, 2016[10]	Sample of ED visits from 356 hospitals, USA	-	14%	25,198 adults	Combination of triage systems and Electronic emergency Triage System	- Hospital admission<br>- ICU admission<br>- Resource use, other<br>- Resource use based on ESI<br>- Composite outcome: in-hospital mortality, intensive care unit admission or transfer to operating room or catheterization suite
Eitel, 2003[11]	7 community hospitals,<br>USA	28,000-90,000	34% (mean)	1042 patients	ESI v.2	- Hospital admission<br>- Resource use based on ESI<br>- Length of stay at the ED<br>- Mortality: 60-day mortality
Erimsah, 2015[12]	Single academic hospital,<br>Turkey	40,000	16%	618 patients	Ministry of Health of Turkey's mandatory emergency triage instrument	- Hospital admission<br>- Resource use, other<br>- Length of stay at the ED<br>- Expert opinion<br>- Immediate lifesaving interventions<br>- Mortality: in-hospital mortality
Fan, 2013[13]	Single tertiary hospital,<br>China (Hong Kong)	128,000	-	100 children and adults	Hong Kong Accident and Emergency Triage Guidelines 4th	- Expert opinion

For peer review only

					version	
Funakoshi, 2016[14]	Single community hospital, Japan	31,793	-	17,121 adults	Japanese Triage and Acuity Scale	- Hospital admission<br>- Length of stay at the ED<br>- Mortality at the ED
Van Gerven, 2001[15]	Single academic hospital, Belgium	-	45%	3650 children and adults	Australian National Triage Scale	- Hospital admission<br>- Sentinel diagnosis
Gouin, 2005[16]	Single academic children's hospital, Canada	65,000	9%	1281 children	CTAS and local triage tool	- Hospital admission<br>- Resource use, other<br>- PRISA score
Gräff, 2014[17]	Single academic hospital, Germany	23,000	33%	45,469 children and adults	MTS	- Hospital admission<br>- ICU admission<br>- Resource use, other<br>- Length of stay at the ED<br>- Mortality: in-hospital mortality<br>- Length of stay in hospital<br>- Level of prehospital care
Gravel, 2009[18]	Single academic hospital, Canada	60,000	9%	5829 children	Ped-CTAS	- Hospital admission<br>- ICU admission<br>- Length of stay at the ED<br>- Mortality at the ED
Gravel, 2012[19]	9 academic hospitals, Canada	43,962	8%	1464 children	CTAS	- Hospital admission<br>- Resource use, other<br>- Length of stay at the ED
Gravel, 2013[20]	12 academic children's hospitals, Canada	25,000-75000	9%	550,940 children	CTAS	- Hospital admission<br>- ICU admission<br>- Length of stay at the ED<br>- Left without being seen
Green, 2012[21]	Single academic children's hospital, USA	45,000	10%	780 children	ESI v.4	- Hospital admission<br>- Resource use based on ESI<br>- Length of stay at the ED
Hansen,	Single hospital,	-	-	550 children	Danish	- Composite outcome "van Veen"; based

For peer review only

2017[22]	Denmark				Regions Pediatric Triage model	on abnormal vital parameters, life-threatening conditions, diagnostics, interventions and follow-up<br>- Composite outcome based on life threatening condition, transfer to higher hospital, ICU, or hospital admission
Hay, 2001[23]	Single hospital, Israel	-	-	2886 patients	Local triage system	- Expert opinion<br>- Waiting time to physician
Van Ierland, 2011[24]	Single academic hospital, the Netherlands	20,000	30% (adults) and 15% (children)	3207 adults and 701 children	Netherlands Triage System	- Hospital admission<br>- ICU admission<br>- Resource use, other<br>- Follow-up at outpatient clinic or GP
Jobe, 2013[25]	Single academic hospital, Belgium	42,000	36%	544 children and adults	Echelle Liégeoise d'Index de Sévérité à l'Admission	- Hospital admission<br>- ICU admission<br>- Resource use, other<br>- Resource use based on ESI<br>- Mortality at the ED
Lee, 2003[26]	4 hospitals, China (Hong Kong)	-	-	2410 patients	5-level triage scale	- Expert opinion
Lee, 2011[27]	Single academic hospital, South Korea	60,000	47%	1903 elderly	CTAS, revised edition	- Hospital admission<br>- ICU admission<br>- Resource use, other<br>- Mortality at the ED<br>- Costs<br>- Immediate lifesaving interventions<br>- Mortality: in-hospital mortality<br>- Length of stay in hospital
Lin, 2016[28]	Single academic hospital, China	217,000	-	275,378 children	Chinese pediatric emergency triage system	- Expert opinion<br>- Waiting time for care<br>- Patients' parents' satisfaction
Ma, 2008[29]	Single academic children's hospital, Canada	50,000	-	1618 children	Ped-CTAS	- Costs

For peer review only - http://bmjopen.bmj.com/site/about/guidelines.xhtml

Maleki, 2015[30]	Single teaching hospital, Iran	74,000	-	387 patients	ESI v.4 and Spot check triage system	- Waiting time to nurse<br>- Waiting time to physician<br>- Waiting time to first lab results
Maningas, 2006[31]	Single community hospital, USA	57,000	6%	7077 children	Soterion Rapid Triage System	- Hospital admission<br>- Length of stay at the ED<br>- Costs<br>- CPT codes
Maningas, 2006[32]	Single community hospital, USA	57,000	10%	33,850 adults	Soterion Rapid Triage System	- Hospital admission<br>- Length of stay at the ED<br>- Costs<br>- CPT codes
Martins, 2009[33]	Single community hospital, Portugal	128,000	10%	316,622 adults	MTS	- Hospital admission<br>- Mortality at the ED
Ng, 2010[34]	3 hospitals, Taiwan	-	34%	1851 adults	CTAS and Taiwan Triage System	- Hospital admission<br>- Length of stay at the ED<br>- Costs
Ng, 2011[35]	11 academic hospitals, 18 regional hospitals and 4 district hospitals, Taiwan	95,912 (average, university hospitals); 47,056 (average regional hospitals); 23,789 (average district hospitals	30%	10,533 adults	Taiwan Triage and Acuity Scale and Taiwan Triage System	- Hospital admission<br>- Resource use based on ESI<br>- Length of stay at the ED
Platts-Mills, 2010[36]	Single academic hospital, USA	64,000	58%	773 elderly	ESI v.4	- Immediate lifesaving interventions
Roukema,	Single teaching	30,000	-	1065	MTS	- Hospital admission

For peer review only

2006[37]	hospital,<br>the Netherlands			children		- Resource use based on ESI<br>- Composite outcome "van Veen"; based on abnormal vital parameters, life-threatening conditions, diagnostics, interventions and follow-up
Santos, 2013[38]	Single hospital, Portugal	-	5%	24,721 adults	MTS v.2	- Hospital admission<br>- Resource use, other<br>- Mortality: in-hospital mortality
Seiger, 2014[39]	2 academic and 2 teaching hospitals, the Netherlands, UK and Portugal	9000-60,000	11% (average)	60,735 children	MTS	- Hospital admission
Steiner, 2016[40]	Single tertiary hospital, Switzerland	-	-	2407 adults	MTS	- ICU admission<br>- Expert opinion<br>- Mortality: 30 day mortality<br>- Length of stay in hospital
Storm-Versloot, 2011[41]	Single academic hospital, the Netherlands	31,000	18%	876 patients	ESI, MTS and informally structured triage system	- Hospital admission<br>- Resource use based on ESI<br>- Length of stay at the ED<br>- Expert opinion
Storm-Versloot, 2014[42]	Single academic hospital,<br>the Netherlands	31,000	21%	906 patients	MTS and informally structured triage system	- Length of stay at the ED<br>- Waiting time from arrival to treatment room<br>- Waiting time from treatment room to discharge from ED<br>- Patient satisfaction
Taboulet, 2009[43]	Single teaching hospital,<br>France	30,000	18%	941 adults	FRench Emergency Nurses Classification in Hospital Scale v.2	- Hospital admission<br>- Resource use, other<br>- Waiting time to physician
Tanabe, 2004[44]	Single academic hospital,	70,000	-	403 children and adults	ESI v.3	- Resource use, other<br>- Length of stay at the ED

For peer review only

	USA					- Length of stay in hospital
Tanabe, 2004[45]	Single academic hospital, USA	70,000	-	403 children and adults	ESI v.3	- Hospital admission<br>- Resource use based on ESI<br>- Length of stay at the ED
Travers, 2002[46]	Single academic hospital, USA	-	-	305 patients	ESI and 3-level system	- Expert opinion
Travers, 2009[47]	1 academic hospital and 4 teaching/community hospitals, USA	13,838-44,987	-	1173 children	ESI v.4	- Hospital admission<br>- Resource use based on ESI<br>- Length of stay at the ED
Twomey, 2013[48]	1 private and 5 public community hospitals, South Africa	-	26%	2014 children	revised paediatric South African Triage Scale	- Hospital admission
Veen, van, 2008[49]	1 academic children's and 1 teaching hospital, the Netherlands	9000-30,000	-	13,554 children	MTS v.1	- Composite outcome "van Veen"; based on abnormal vital parameters, life-threatening conditions, diagnostics, interventions and follow-up
Veen, van, 2012[50]	1 academic children's and 1 teaching hospital, the Netherlands	9000-15,000	-	11,260 children	MTS incl. modifications for children with fever	- Composite outcome "van Veen"; based on abnormal vital parameters, life-threatening conditions, diagnostics, interventions and follow-up
Waldrop, 1997[51]	Single teaching hospital, USA	65,000	5% (adults), 8% (children)	13,294 adults and 1528 children	Local triage system	- Hospital admission<br>- Mortality at the ED<br>- Length of stay at the ED<br>- Left without being seen<br>- Waiting time to examination
Worster, 2007[52]	2 academic hospitals, Canada	36,000-395,000	-	481 patients	CTAS and ESI	- Resource use, other<br>- Mortality: in-hospital mortality
Wuerz, 2000[53]	2 academic hospitals, USA	60,000 (average)	32%	493 adults	ESI	- Hospital admission<br>- Resource use based on ESI<br>- Length of stay at the ED

For peer review only

						- Costs
Wuerz, 2001[54]	Single academic hospital, USA	50,000	-	180 adults	ESI	- Mortality: 6 month mortality
Wuerz, 2001[55]	2 academic hospitals, USA	50,000-60,000	28%	8251 adults	ESI	- Hospital admission<br>- Length of stay at the ED
Wulp, van der 2009[56]	4 teaching hospitals, the Netherlands	30,000-47,000	21%	72,232 children and adults	ESI and MTS	- Hospital admission<br>- Mortality at the ED
Wulp, van der, 2011[57]	Single academic hospital, the Netherlands	21,000	-	544 adults	ESI	- Worthing Phsyiological Scoring System<br>- Pain score
Yuksen, 2016[58]	Single academic hospital, Thailand	-	-	520 patients	ESI and 4-level nurse triage	- Hospital admission<br>- Resource use based on ESI<br>- Length of stay at the ED<br>- Mortality at the ED<br>- Immediate lifesaving interventions
Zachariasse, 2016[59]	Single academic children's hospital, the Netherlands	7000	-	50,062 children	MTS	- ICU admission
Zachariasse, 2017[60]	1 academic children's hospital, 1 teaching hospital and 1 community hospital, the Netherlands and Portugal	7000 – 190,000	-	222,603 adults and 66,060 children	MTS	- ICU admission<br>- Composite outcome based on physiologic parameters, patient disposition and resource use

Ped, Pediatric; *CTAS*, Canadian Triage and Acuity Scale; *MTS*, Manchester Triage System; *ESI*, Emergency Severity Index; *TTS*, Taiwan Triage System

For peer review only

References

1. Blunt I, Edwards N, Merry L. What's behind the A&E 'crisis'? *Briefing*: Nuffield Trust; 2015.
2. Australasian College for Emergency Medicine. Access Block and Overcrowding in Emergency departments. 2004.
3. Institute of Medicine. Hospital-based emergency care: at the breaking point. 2013.
4. Mackway-Jones K, Marsden J, Windle J. *Emergency triage*. London: BMJ; 2006.
5. Warren DW, Jarvis A, LeBlanc L, et al. Revisions to the Canadian Triage and Acuity Scale paediatric guidelines (PaedCTAS). *Cjem.* 2008;10:224-243.
6. Gilboy NT, P.; Travers, D.A.; Rosenau, A.M.; Eitel, D.R. *Emergency Severity Index, Version 4: Implementation Handbook*. Rockville, MD: Agency for Healthcare Research and Quality; 2005.
7. Australian College for Emergency Medicine. Guidelines on the implementation of the australasian triage scale in emergency departments. http://www.acem.org.au/media/policies_and_guidelines/G24_Implementation__ATS.pdf.
8. Christ M, Grossmann F, Winter D, Bingisser R, Platz E. Modern triage in the emergency department. *Dtsch Arztebl.* 2010;107:892-898.
9. Farrohknia N, Castren M, Ehrenberg A, et al. Emergency department triage scales and their components: a systematic review of the scientific evidence. *Scand J Trauma Resusc Emerg Med.* 2011;19:42.
10. Parenti N, Ferrara L, Bacchi Reggiani ML, Sangiorgi D, Lenzi T. Reliability and validity of two four-level emergency triage systems. *Eur J Emerg Med.* 2009;16:115-120.
11. Stroup DF, Berlin JA, Morton SC, et al. Meta-analysis of observational studies in epidemiology: a proposal for reporting. Meta-analysis Of Observational Studies in Epidemiology (MOOSE) group. *Jama.* 2000;283:2008-2012.
12. World Bank Group. World Bank Country and Lending Groups. https://datahelpdesk.worldbank.org/knowledgebase/articles/906519-world-bank-country-and-lending-groups. Accessed October 5th, 2017.
13. Whiting PF, Rutjes AW, Westwood ME, et al. QUADAS-2: a revised tool for the quality assessment of diagnostic accuracy studies. *Ann Intern Med.* 2011;155:529-536.
14. Kuriyama A, Urushidani S, Nakayama T. Five-level emergency triage systems: variation in assessment of validity. *Emerg Med J.* 2017.
15. R Development Core Team. R: A language and environment for statistical computing. Vienna, Austria: R Foundation for Statistical Computing; 2008.
16. IBM Corp. IBM SPSS Statistics for Windows. 21 ed. Armonk, NY 2011.
17. Review Manager (RevMan). 5.3 ed. Copenhagen: The Nordic Cochrane Centre, The Cochrane Collaboration; 2014.
18. Storm-Versloot MN, Ubbink DT, Kappelhof J, Luitse JSK. Comparison of an informally structured triage system, the emergency severity index, and the manchester triage system to distinguish patient priority in the emergency department. *Acad Emerg Med.* 2011;18:822-829.
19. van der Wulp I, Schrijvers AJP, van Stel HF. Predicting admission and mortality with the Emergency Severity Index and the Manchester Triage System: a retrospective observational study. *Emergency Medicine Journal.* 2009;26:506-509.
20. Wuerz RC, Travers D, Gilboy N, Eitel DR, Rosenau A, Yazhari R. Implementation and refinement of the Emergency severity index. *Acad Emerg Med.* 2001;8:170-176.
21. Ng CJ, Yen ZS, Tsai JCH, et al. Validation of the Taiwan triage and acuity scale: A new computerised five-level triage system. *Emerg Med J.* 2011;28:1026-1031.
22. Seiger N, van Veen M, Almeida H, et al. Improving the Manchester Triage System for pediatric emergency care: an international multicenter study. *PLoS One.* 2014;9:e83267.

For peer review only

23. Zachariasse JM, Seiger N, Rood PPM, et al. Validity of the Manchester triage system in emergency care: A prospective observational study. *PLoS ONE.* 2017;12.
24. Azeredo TR, Guedes HM, Rebelo de Almeida RA, Chianca TC, Martins JC. Efficacy of the Manchester Triage System: a systematic review. *Int Emerg Nurs.* 2015;23:47-52.
25. Souza CC, Araujo FA, Chianca TC. [Scientific Literature on the Reliability and Validity of the Manchester Triage System (MTS) Protocol: A Integrative Literature Review]
Producao cientifica sobre a validade e confiabilidade do Protocolo de Manchester: revisao integrativa da literature. *Rev Esc Enferm USP.* 2015;49:144-151.
26. van Veen M, Moll HA. Reliability and validity of triage systems in paediatric emergency care. *Scand J Trauma Resusc Emerg Med.* 2009;17:38.
27. de Magalhaes-Barbosa MC, Robaina JR, Prata-Barbosa A, Lopes CS. Validity of triage systems for paediatric emergency care: a systematic review. *Emerg Med J.* 2017.
28. www.esitriage.com. Accessed October 5th, 2017. .
29. http://caep.ca/resources/ctas. Accessed October 5th, 2017.
30. www.triagenet.net. Accessed October 5th, 2017. Vol 2017.
31. Moll HA. Challenges in the validation of triage systems at emergency departments. *J Clin Epidemiol.* 2010;63:384-388.

For peer review only

Appendix 3. Risk of bias assessment

Table 3.1 Risk of bias and applicability concerns summary: review authors' judgements about each domain for each included study

For peer review only

	Risk of Bias				Applicability Concerns		
	Patient Selection	Index Test	Reference Standard	Flow and Timing	Patient Selection	Index Test	Reference Standard
Al-Hindi 2014	+	+	–	–	+	+	?
Allon 2017	+	+	+	+	+	+	?
Baumann 2005	+	+	+	+	+	+	?
Baumann 2006	+	+	+	+	–	+	?
Brillman 1996	+	+	–	–	+	–	?
Bruijns 2008	+	+	+	–	+	+	?
Chang 2013	+	+	+	+	–	+	?
Chi 2006	–	–	–	–	+	–	?
Dong 2007	+	?	+	+	+	+	?
Dugas 2016	+	–	+	–	–	–	?
Eitel 2003	+	+	–	+	+	+	?
Erimsah 2015	+	+	?	–	+	–	?
Fan 2012	+	+	+	+	+	+	?
Funakoshi 2016	+	+	+	+	+	+	?
Gerven, van 2001	+	–	–	+	+	+	?
Gouin 2005	+	+	–	+	+	+	?
Gräff 2014	+	+	+	+	–	+	?
Gravel 2009	+	?	+	+	+	+	?
Gravel 2012	–	+	+	+	+	+	?
Gravel 2013	+	+	+	+	+	+	?
Green 2012	+	+	–	+	+	+	?
Hansen 2017	–	–	+	–	+	+	?
Hay 2001	–	+	–	–	+	–	?
Ierland, van 2011	+	+	+	–	+	+	?
Jobe 2013	+	?	+	?	+	+	?
Lee 2003	–	+	+	–	?	?	?
Lee 2011	+	+	+	+	+	+	?
Lin 2016	+	+	–	+	+	+	?
Ma 2008	+	+	+	+	+	+	?
Maleki 2015	+	+	?	–	+	+	?
Maningas 2006	–	+	?	+	+	+	?
Maningas 2006b	–	+	+	+	+	+	?
Martins 2009	+	+	+	+	+	+	?
Ng 2010	+	–	?	–	+	+	?
Ng 2011	–	+	+	–	+	+	?
Platts-Mills 2010	+	+	–	+	+	+	?
Roukema 2006	+	+	+	+	+	+	?
Santos 2013	–	+	+	+	+	+	?
Seiger 2014	+	+	+	+	+	+	?
Steiner 2016	+	+	+	+	–	+	?

view only

Appendix 4. Reference standards used in studies evaluating triage systems in the emergency department

**Patient disposition and follow-up**	Hospital admission<br>ICU admission<br>Follow-up at outpatient clinic or GP	41 studies<br>12 studies<br>1 study
**Waiting times**	Length of stay at the ED<br>Length of stay in hospital<br>Waiting time to physician<br>Other: Waiting time to nurse, Waiting time to examination, Waiting time to first lab results, Waiting time from arrival to treatment room, Waiting time from treatment room to discharge from ED, Waiting time for care	26 studies<br>4 studies<br>4 studies<br>1 study each
**Resource use**	Resource use based on ESI criteria<br>Costs<br>Current Procedural Terminology (CPT) codes<br>Other	13 studies<br>8 studies<br>2 studies<br>14 studies
**Mortality**	Mortality at the ED<br>In-hospital mortality<br>1 year mortality<br>Other: 30-day mortality, 60-day mortality, 6 month mortality	12 studies<br>5 studies<br>2 studies<br>1 study each
**Composite outcomes**	"Immediate lifesaving interventions"<br>"MTS reference standard": Combination of abnormal physiologic parameters, life-threatening conditions, resource use, follow-up<br>"Critical or time-sensitive outcomes": in-hospital mortality, intensive care unit admission or transfer to operating room or catheterization suite<br>"Actual clinical outcomes": Combination of life threatening condition, transfer to higher hospital, ICU, or hospital admission<br>Composite outcome based on physiologic parameters, patient disposition and resource use	4 studies<br>4 studies<br>1 study<br>1 study<br>1 study
**Physiologic parameters**	Worthing Phsyiological Scoring System<br>Pain score	1 study<br>1 study
**Other**	Expert opinion<br>Left without being seen<br>Patient or parent satisfaction<br>Level of prehospital care<br>Sentinel diagnosis<br>Pediatric risk of admission (PRISA) score	9 studies<br>5 studies<br>2 studies<br>1 study<br>1 study<br>1 study

For peer review only

Appendix 5. Reference standard per urgency category

Figure 5.1 Proportion of ICU admissions per urgency category in the most commonly evaluated triage systems.

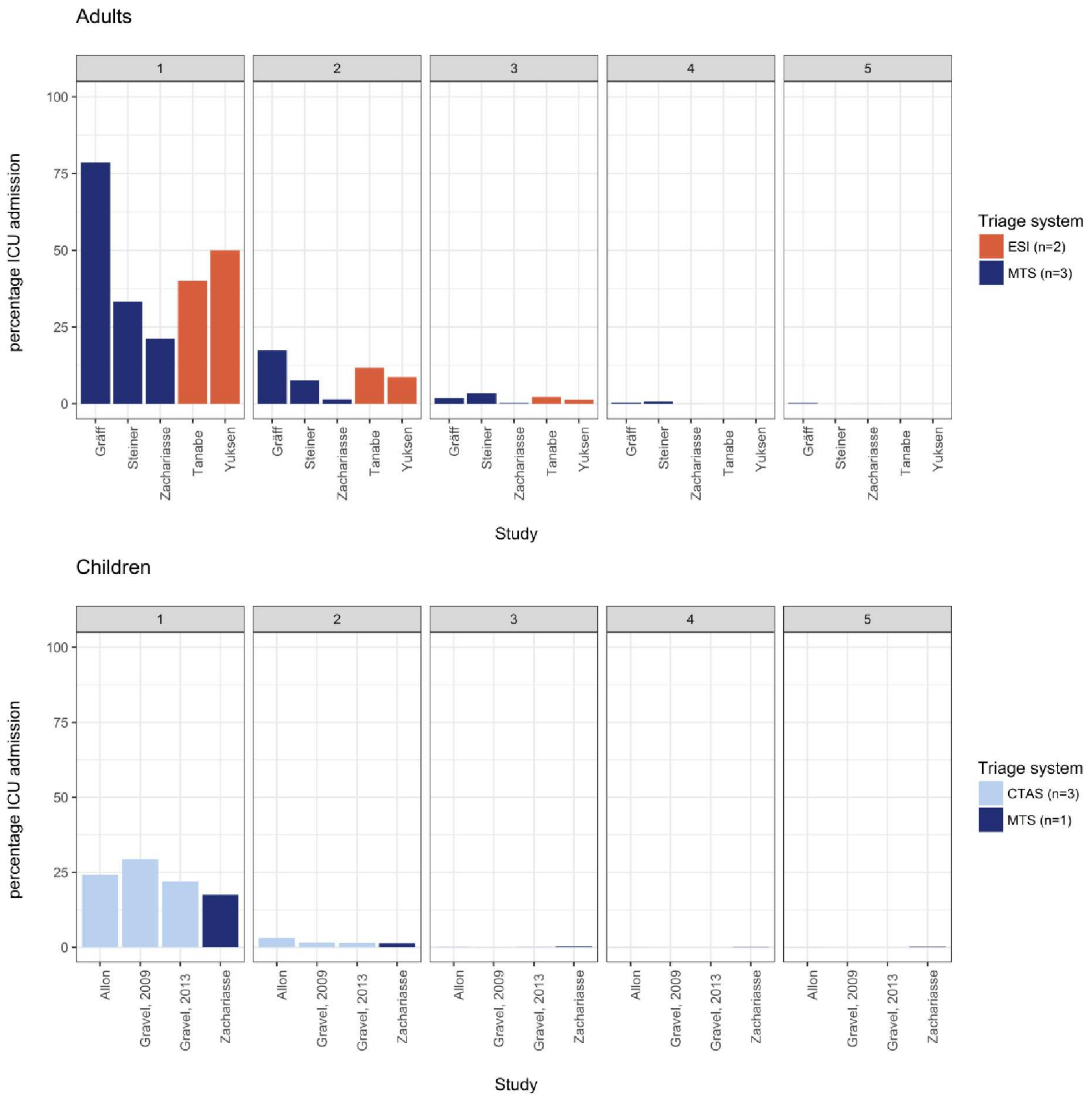

Figure 5.2 Proportion of patients discharged home per urgency category in the most commonly evaluated triage systems

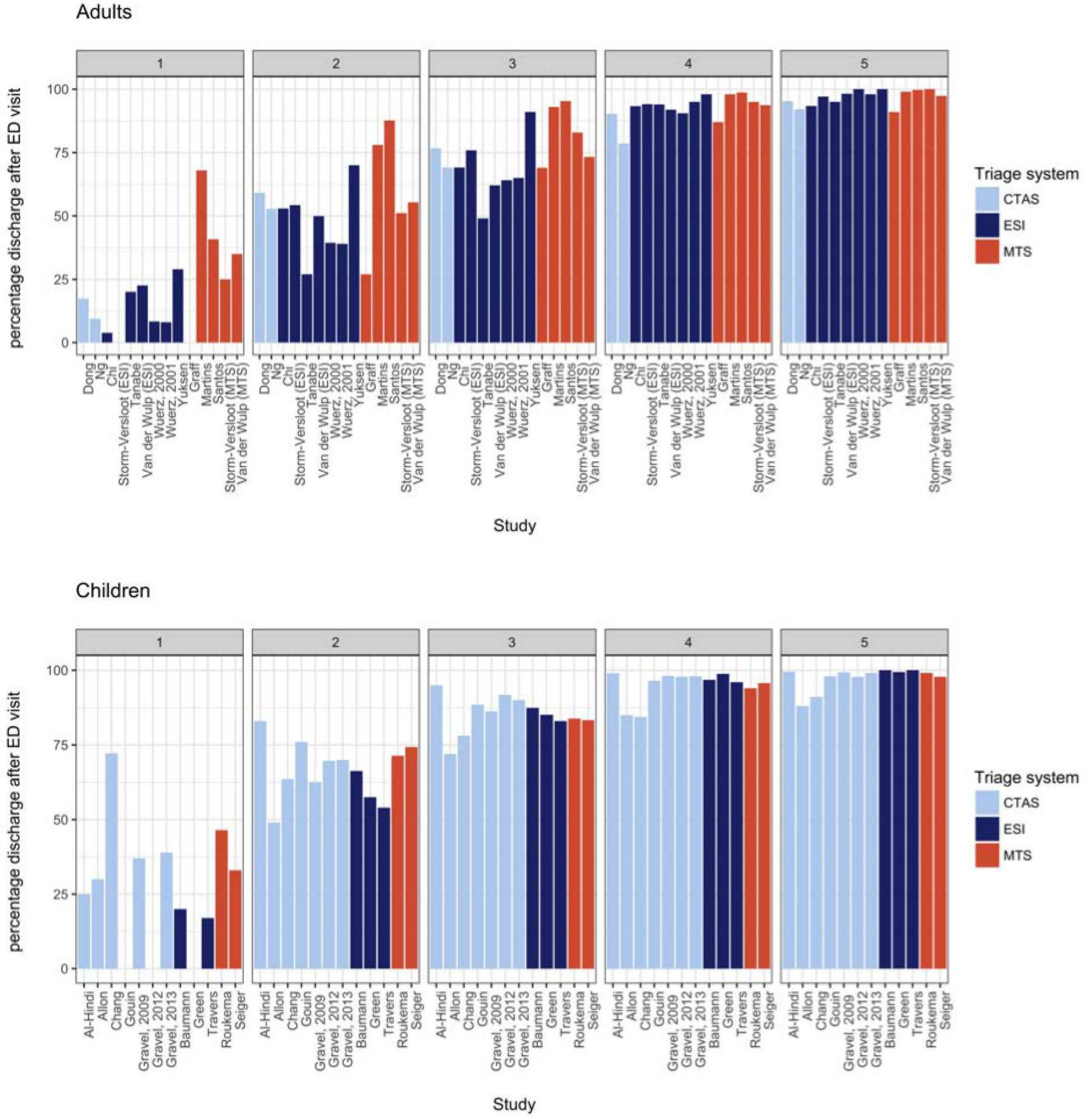

Appendix 6. Other reference standards

Other reference standards

13 studies used this reference standard of which 6 provided data to construct a 2x2 table and were conducted in one of the commonly used triage systems. One study evaluated resource use in the MTS, and all others studied the ESI.

Because the ESI states that resource prediction should only be used for the less acute patients, we used a different cut-off for dichotomization to calculate sensitivity and specificity. We dichotomized the triage categories into a high urgent group consisting of triage urgency levels 1, 2 and 3 with a low urgent group consisting of triage categories 4 and 5.

Overall, sensitivity of the triage systems to accurately classify patients with 2 or more resources in the highest urgency categories ranged from 0.87 (95%CI 0.82 to 0.91) to 0.91 (95%CI 0.87 to 0.94) in adults and from 0.76 (95%CI 0.70 to 0.82) to 0.90 (95%CI 0.87 to 0.92) in children (Fig. 7). Specificity of the triage system to accurately classify patients with 1 or 0 resources in the lowest two urgency categories ranged from 0.73 (95%CI 0.66 to 0.79) to 0.75 (95%CI 0.69 to 0.81) in adults and from 0.42 (95%CI 0.39 to 0.46) to 0.75 (95%CI 0.69 to 0.81) in children.

For peer review only

Fig 6.1. Forest plot evaluating sensitivity and specificity of triage systems using resource use according to ESI criteria as reference standard

ESI

Study	TP	FP	FN	TN	Age group	Sensitivity (95% CI)	Specificity (95% CI)
Baumann 2005	101	183	11	215	Children	0.90 [0.83, 0.95]	0.54 [0.49, 0.59]
Travers 2009	480	200	56	438	Children	0.90 [0.87, 0.92]	0.69 [0.65, 0.72]
Green 2012	158	206	49	367	Children	0.76 [0.70, 0.82]	0.64 [0.60, 0.68]
Wuerz 2000	241	59	36	157	Adults / Unspecified	0.87 [0.82, 0.91]	0.73 [0.66, 0.79]
Yuksen 2016	278	53	29	160	Adults / Unspecified	0.91 [0.87, 0.94]	0.75 [0.69, 0.81]

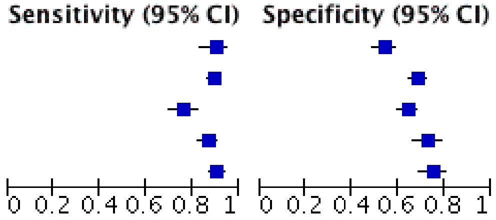

MTS

Study	TP	FP	FN	TN	Age group	Sensitivity (95% CI)	Specificity (95% CI)
Roukema 2006	205	469	51	340	Children	0.80 [0.75, 0.85]	0.42 [0.39, 0.46]

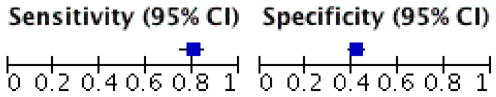

ESI = Emergency Severity Index; TP = true positive; FP = false positive; FN = false negative; TN = true negative; MTS = Manchester Triage System

review only

For peer review only - http://bmjopen.bmj.com/site/about/guidelines.xhtml

Appendix 7. Determinants of triage systems' performance

Figure 7.1. Forest plot evaluating sensitivity and specificity of triage systems for identifying low urgency patients as defined by discharge home after the ED visit, ordered by annual census as a marker of patient volume

Adults

Study	TP	FP	FN	TN	Annual Census	Sensitivity (95% CI)	Specificity (95% CI)
Graff 2014	6497	2132	8463	28377	23000.0	0.43 [0.43, 0.44]	0.93 [0.93, 0.93]
Storm-Versloot 2011	73	72	76	651	31000.0	0.49 [0.41, 0.57]	0.90 [0.88, 0.92]
Storm-Versloot 2011	66	76	96	638	31000.0	0.41 [0.33, 0.49]	0.89 [0.87, 0.92]
Yuksen 2016	38	68	22	392	33936.0	0.63 [0.50, 0.75]	0.85 [0.82, 0.88]
Van der Wulp 2009	2641	3183	4415	24019	38500.0	0.37 [0.36, 0.39]	0.88 [0.88, 0.89]
Van der Wulp 2009	2259	2146	5911	27658	38500.0	0.28 [0.27, 0.29]	0.93 [0.93, 0.93]
Wuerz 2001	1113	674	1260	5204	55000.0	0.47 [0.45, 0.49]	0.89 [0.88, 0.89]
Chi 2006	713	747	376	1336	58000.0	0.65 [0.63, 0.68]	0.64 [0.62, 0.66]
Wuerz 2000	91	53	68	281	60000.0	0.57 [0.49, 0.65]	0.84 [0.80, 0.88]
Dong 2007	1519	1947	4189	21869	67000.0	0.27 [0.25, 0.28]	0.92 [0.91, 0.92]
Tanabe 2004	129	48	75	151	70000.0	0.63 [0.56, 0.70]	0.76 [0.69, 0.82]
Santos 2013	485	3080	535	19515	100000.0	0.48 [0.44, 0.51]	0.86 [0.86, 0.87]
Martins 2009	18265	63803	12464	222090	128000.0	0.59 [0.59, 0.60]	0.78 [0.78, 0.78]

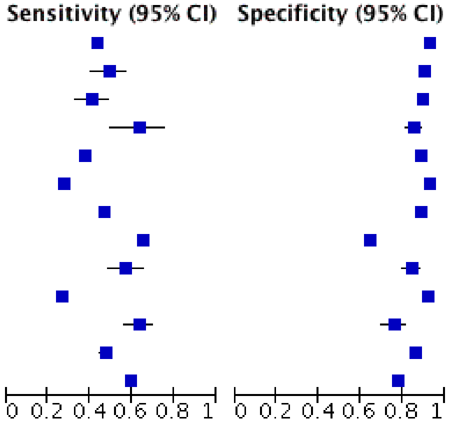

Children

Study	TP	FP	FN	TN	Annual Census	Sensitivity (95% CI)	Specificity (95% CI)
Allon 2017	2631	2330	17726	60922	16722.0	0.13 [0.12, 0.13]	0.96 [0.96, 0.96]
Roukema 2006	147	256	62	600	30000.0	0.70 [0.64, 0.76]	0.70 [0.67, 0.73]
Chang 2013	3104	6821	6276	26327	42839.0	0.33 [0.32, 0.34]	0.79 [0.79, 0.80]
Green 2012	33	42	47	658	45000.0	0.41 [0.30, 0.53]	0.94 [0.92, 0.96]
Baumann 2005	44	66	28	372	53000.0	0.61 [0.49, 0.72]	0.85 [0.81, 0.88]
Gravel 2009	1937	2746	3124	50722	60000.0	0.38 [0.37, 0.40]	0.95 [0.95, 0.95]
Gouin 2005	8	19	38	472	65000.0	0.17 [0.08, 0.31]	0.96 [0.94, 0.98]
Al-Hindi 2014	65	295	45	2609	155000.0	0.59 [0.49, 0.68]	0.90 [0.89, 0.91]

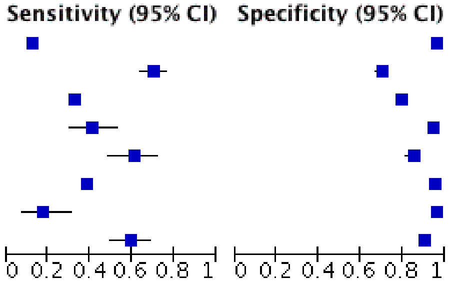

TP = true positive; FP = false positive; FN = false negative; TN = true negative

Figure 7.2. Forest plot evaluating sensitivity and specificity of triage systems for identifying low urgency patients as defined by discharge home after the ED visit, ordered by percentage hospitalization as a marker of case-mix severity of illness

Adults

Study	TP	FP	FN	TN	Hospitalization rate	Sensitivity (95% CI)	Specificity (95% CI)
Santos 2013	485	3080	535	19515	5.0	0.48 [0.44, 0.51]	0.86 [0.86, 0.87]
Martins 2009	18265	63803	12464	222090	10.0	0.59 [0.59, 0.60]	0.78 [0.78, 0.78]
Yuksen 2016	38	68	22	392	12.0	0.63 [0.50, 0.75]	0.85 [0.82, 0.88]
Storm-Versloot 2011	73	72	76	651	18.0	0.49 [0.41, 0.57]	0.90 [0.88, 0.92]
Storm-Versloot 2011	66	76	96	638	18.0	0.41 [0.33, 0.49]	0.89 [0.87, 0.92]
Dong 2007	1519	1947	4189	21869	19.0	0.27 [0.25, 0.28]	0.92 [0.91, 0.92]
Van der Wulp 2009	2641	3183	4415	24019	21.0	0.37 [0.36, 0.39]	0.88 [0.88, 0.89]
Van der Wulp 2009	2259	2146	5911	27658	21.0	0.28 [0.27, 0.29]	0.93 [0.93, 0.93]
Wuerz 2001	1113	674	1260	5204	28.0	0.47 [0.45, 0.49]	0.89 [0.88, 0.89]
Wuerz 2000	91	53	68	281	32.0	0.57 [0.49, 0.65]	0.84 [0.80, 0.88]
Graff 2014	6497	2132	8463	28377	33.0	0.43 [0.43, 0.44]	0.93 [0.93, 0.93]
Chi 2006	713	747	376	1336	34.0	0.65 [0.63, 0.68]	0.64 [0.62, 0.66]

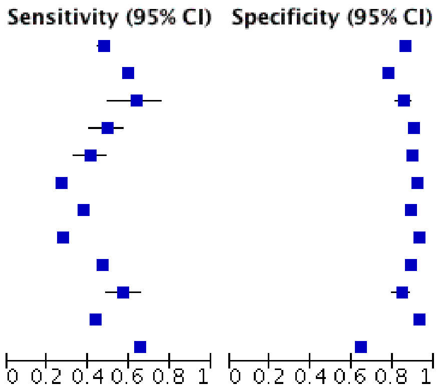

Children

Study	TP	FP	FN	TN	Hospitalization rate	Sensitivity (95% CI)	Specificity (95% CI)
Al-Hindi 2014	65	295	45	2609	4.0	0.59 [0.49, 0.68]	0.90 [0.89, 0.91]
Gravel 2009	1937	2746	3124	50722	9.0	0.38 [0.37, 0.40]	0.95 [0.95, 0.95]
Gouin 2005	8	19	38	472	9.0	0.17 [0.08, 0.31]	0.96 [0.94, 0.98]
Green 2012	33	42	47	658	10.0	0.41 [0.30, 0.53]	0.94 [0.92, 0.96]
Baumann 2005	44	66	28	372	14.0	0.61 [0.49, 0.72]	0.85 [0.81, 0.88]
Chang 2013	3104	6821	6276	26327	23.0	0.33 [0.32, 0.34]	0.79 [0.79, 0.80]
Allon 2017	2631	2330	17726	60922	24.0	0.13 [0.12, 0.13]	0.96 [0.96, 0.96]

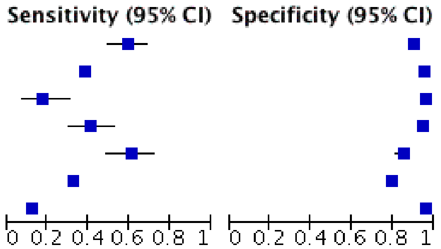

TP = true positive; FP = false positive; FN = false negative; TN = true negative

MOOSE Checklist for Meta-analyses of Observational Studies

Item No	Recommendation	Reported on Page No
Reporting of background should include		
1	Problem definition	p.6
2	Hypothesis statement	-
3	Description of study outcome(s)	p.6, 7, 8
4	Type of exposure or intervention used	p.6,7
5	Type of study designs used	p.7
6	Study population	p.6,7
Reporting of search strategy should include		
7	Qualifications of searchers (eg, librarians and investigators)	Title page
8	Search strategy, including time period included in the synthesis and key words	p.7, appendix 1
9	Effort to include all available studies, including contact with authors	p.7
10	Databases and registries searched	p.7, appendix 1
11	Search software used, name and version, including special features used (eg, explosion)	-
12	Use of hand searching (eg, reference lists of obtained articles)	Fig. 1
13	List of citations located and those excluded, including justification	Fig. 1, appendix 2
14	Method of addressing articles published in languages other than English	p.7
15	Method of handling abstracts and unpublished studies	p.7
16	Description of any contact with authors	-
Reporting of methods should include		
17	Description of relevance or appropriateness of studies assembled for assessing the hypothesis to be tested	p.7
18	Rationale for the selection and coding of data (eg, sound clinical principles or convenience)	p.7
19	Documentation of how data were classified and coded (eg, multiple raters, blinding and interrater reliability)	p.7
20	Assessment of confounding (eg, comparability of cases and controls in studies where appropriate)	-
21	Assessment of study quality, including blinding of quality assessors, stratification or regression on possible predictors of study results	p.8
22	Assessment of heterogeneity	p.9
23	Description of statistical methods (eg, complete description of fixed or random effects models, justification of whether the chosen models account for predictors of study results, dose-response models, or cumulative meta-analysis) in sufficient detail to be replicated	p.9
24	Provision of appropriate tables and graphics	Tables 1-2, Figs 1-9
Reporting of results should include		
25	Graphic summarizing individual study estimates and overall estimate	Figs 3,5,7
26	Table giving descriptive information for each study included	Appendix 2
27	Results of sensitivity testing (eg, subgroup analysis)	Figs 8-9
28	Indication of statistical uncertainty of findings	-

For peer review only

Item No	Recommendation	Reported on Page No
Reporting of discussion should include		
29	Quantitative assessment of bias (eg, publication bias)	p.29-30
30	Justification for exclusion (eg, exclusion of non-English language citations)	-
31	Assessment of quality of included studies	p.29-30
Reporting of conclusions should include		
32	Consideration of alternative explanations for observed results	p.30-31
33	Generalization of the conclusions (ie, appropriate for the data presented and within the domain of the literature review)	p.30-31
34	Guidelines for future research	p.31
35	Disclosure of funding source	p.3

From: Stroup DF, Berlin JA, Morton SC, et al, for the Meta-analysis Of Observational Studies in Epidemiology (MOOSE) Group. Meta-analysis of Observational Studies in Epidemiology. A Proposal for Reporting. *JAMA*. 2000;283(15):2008-2012. doi: 10.1001/jama.283.15.2008.

For peer review only

BMJ Open

THE PERFORMANCE OF TRIAGE SYSTEMS IN EMERGENCY CARE: A SYSTEMATIC REVIEW AND META-ANALYSIS

Journal:	*BMJ Open*
Manuscript ID	bmjopen-2018-026471.R1
Article Type:	Research
Date Submitted by the Author:	17-Jan-2019

Complete List of Authors:	Zachariasse, Joany; Erasmus MC-Sophia Childrens Hospital, Department of Pediatrics<br>van der Hagen, Vera; Erasmus MC-Sophia Childrens Hospital, Department of Pediatrics<br>Seiger, Nienke; Erasmus MC-Sophia Childrens Hospital, Department of Pediatrics<br>Mackway-Jones, Kevin; Manchester Royal Infirmary, Emergency Department<br>van Veen, Mirjam; Groene Hart Ziekenhuis, Pediatrics; Erasmus MC-Sophia Childrens Hospital, Department of Pediatrics<br>Moll, Henriette; Erasmus MC-Sophia Childrens Hospital, Department of Pediatrics

**Primary Subject Heading**:	Emergency medicine
Secondary Subject Heading:	Epidemiology
Keywords:	ACCIDENT & EMERGENCY MEDICINE, EPIDEMIOLOGY, Paediatric A&E and ambulatory care < PAEDIATRICS, PUBLIC HEALTH

SCHOLARONE™
Manuscripts

THE PERFORMANCE OF TRIAGE SYSTEMS IN EMERGENCY CARE: A SYSTEMATIC REVIEW AND META-ANALYSIS

Joany M Zachariasse*, Vera van der Hagen*, Nienke Seiger, Kevin C Mackway-Jones, Mirjam van Veen, Henriëtte A Moll

* Both authors contributed equally

Department of Paediatrics, Erasmus MC- Sophia Children's Hospital, University Medical Centre Rotterdam, PO Box 2060, 3000 CB Rotterdam, The Netherlands
Joany M Zachariasse
PhD-student

Department of Paediatrics, Erasmus MC- Sophia Children's Hospital, University Medical Centre Rotterdam, PO Box 2060, 3000 CB Rotterdam, The Netherlands
Vera van der Hagen
MD

Department of Paediatrics, Erasmus MC- Sophia Children's Hospital, University Medical Centre Rotterdam, PO Box 2060, 3000 CB Rotterdam, The Netherlands
Nienke Seiger
Paediatric resident

Emergency Department, Manchester Royal Infirmary, Oxford Road, Manchester M13 9WL, UK
Kevin C Mackway Jones
Professor of Emergency Medicine

Department of Paediatrics, Erasmus MC- Sophia Children's Hospital, University Medical Centre Rotterdam, PO Box 2060, 3000 CB Rotterdam, The Netherlands
Currently: Department of Paediatrics, Groene Hart Ziekenhuis, Postal Code 1098, 2800 BB Gouda, The Netherlands
Mirjam van Veen
Paediatrician

For peer review only

For peer review only - http://bmjopen.bmj.com/site/about/guidelines.xhtml

Department of Paediatrics, Erasmus MC- Sophia Children's Hospital, University Medical Centre Rotterdam, PO Box 2060, 3000 CB Rotterdam, The Netherlands

Henriëtte A Moll

Professor of Paediatrics

Correspondence to:
Henriëtte A Moll
h.a.moll@erasmusmc.nl

For peer review only

Word count: 3995

Word count abstract: 275

Abbreviations:

ATS Australasian Triage Scale

CTAS Canadian Triage and Acuity Scale

ED emergency department

ESI Emergency Severity Index

FN false negative

FP false positive

MTS Manchester Triage System

TN true negative

TP true positive

Funding: This research received no specific grant from any funding agency in the public, commercial or not-for-profit sectors

Competing interests: No support from any organisation for the submitted work; KMJ is chair of the Manchester Triage Group; there are no other relationships or activities that could appear to have influenced the submitted work.

Dating sharing: No additional data available

For peer review only - http://bmjopen.bmj.com/site/about/guidelines.xhtml

Abstract

Objective

To assess and compare the performance of triage systems for identifying high and low urgency patients in the emergency department

Design

Systematic review and meta-analysis

Data sources

EMBASE, Medline OvidSP, Cochrane central, Web-of-science and CINAHL databases from 1980 to 2016 with the final update in December 2018.

Eligibility criteria

Studies that evaluated an emergency medical triage system, assessed validity using any reference standard as proxy for true patient urgency and were written in English. Studies conducted in low(er)-income countries, based on case-scenarios or involving less than 100 patients were excluded.

Review methods

Reviewers identified studies, extracted data, and assessed the quality of the evidence independently and in duplicate. The QUADAS-2 checklist was used to assess risk of bias. Raw data were extracted to create 2x2 tables and calculate sensitivity and specificity. ED patient volume and case-mix severity of illness were investigated as determinants of triage systems' performance.

Results

Sixty-six eligible studies evaluated thirty-three different triage systems. Comparisons were restricted to the three triage systems that had at least multiple evaluations using the same reference standard (Canadian Triage and Acuity Scale, Emergency Severity Index, and Manchester Triage System). Overall validity to identify high and low urgency patients of each triage system was moderate to good, but performance was highly variable. In a subgroup analysis, no clear association was found between ED patient volume or case-mix severity of illness and triage systems' performance.

Conclusions

Established triage systems show a reasonable validity for the triage of patients at the ED, but performance varies considerably. Important research questions that remain are what determinants influence triage systems' performance and how the performance of existing triage systems can be improved.

For peer review only

For peer review only - http://bmjopen.bmj.com/site/about/guidelines.xhtml

Strengths and limitations of the study

- The present study was based on a comprehensive and systematic search and attempted to include as many relevant studies as possible

- This study is the first synthesis of the available evidence on triage systems as previous reviews have merely described the results of the individual studies .

- The majority of triage systems were evaluated by less than three studies. Even for the three most commonly used triage systems, few evaluations were available due to the variety of reference standards

- Due to the limited number of studies and the heterogeneity of study populations it was difficult to compare results across studies and we could not provide summary estimates.

For peer review only - http://bmjopen.bmj.com/site/about/guidelines.xhtml

Introduction

Overcrowding of emergency departments (EDs) is a universal and ever increasing problem.[1-3] Therefore, most EDs have a triage system in place to facilitate the prioritisation of patients. In recent years several formal triage scales have been developed to standardize the approach to triage. These include amongst others the Australasian Triage Scale, the Canadian Triage and Acuity Scale (CTAS), the Emergency Severity Index (ESI) and the Manchester Triage System (MTS).[4-7]

It is important to evaluate the performance of triage systems for their ability to accurately distinguish between both high and low urgency patients. The correct classification of high urgency patients is related to patient safety, because misclassification of high urgency patients to a low urgency level causes delay in diagnosis and treatment, potentially leading to morbidity or mortality. The correct classification of low urgency patients increases efficiency of the ED flow and reduces waiting times for the truly high urgency ED visits.

Research regarding the performance of triage systems mainly consists of observational studies in heterogeneous populations using a variety of reference standards. Previous reviews have primarily described the results of these individual studies without combining and interpreting the evidence into overall conclusions.[8-10] A systematic appraisal of the performance of commonly used triage systems can inform clinicians and policy makers about the safety and efficacy of available triage systems and provide insights on which triage system is safe and efficient to use. Moreover it can highlight gaps in current research and propose directions for future studies.

The aim of this systematic review is to provide a comprehensive overview of current evidence on the performance of triage systems. We assessed and compared the performance of the most commonly used triage systems for the prioritisation of high- and low urgency patients at the ED, compared to any reference standard that is a proxy of true patient urgency. Furthermore, we aimed to investigate whether patient volume and case-mix at the ED, are determinants of triage systems' performance.

For peer review only - http://bmjopen.bmj.com/site/about/guidelines.xhtml

Methods

Search strategy

Meta-analysis of Observational Studies in Epidemiology (MOOSE) guidelines were followed for the conduct of this study.[11] We conducted a systematic review using a broad search strategy to identify all studies assessing the performance of triage systems in emergency care when compared to any reference standard that is a proxy of true patient urgency. A search strategy was developed by a health sciences librarian and included medical subject headings (MeSH) and text words related to triage, emergency care and validity (Appendix 1). We searched EMBASE, Medline OvidSP, Cochrane central, Web-of-science and CINAHL databases from 1980 to 2016 with the final update in December 2018.

Study eligibility

Studies were selected that assess the performance of triage systems in emergency care with a defined outcome measure as a proxy of true patient-urgency. We selected studies based on the following PICO:

Population: We included studies evaluating triage in the general populations of patients attending the emergency department. We excluded studies restricted to specific patient subgroups (such as specific diseases).

Interventions: We included any studies assessing emergency department triage systems, defined as any tool aimed to classify patients at the emergency department based on the urgency or severity of their condition. We did not include studies evaluating trauma triage systems or early warning scores.

Comparators: Since no golden standard for the evaluation of triage systems exists, we included all studies evaluating the performance of triage systems using one or more defined reference standard as a proxy for true patient urgency.

Outcome: We defined outcome as the sensitivity and specificity of the triage system for the identification of high-urgent and low-urgent patients. A-priori, we selected mortality at the ED and ICU admission after the ED visit as reference standard for high-urgency and discharge home after the ED visit as reference standard for low urgency. We additionally considered any other reference standard with sufficient evaluations.

For peer review only - http://bmjopen.bmj.com/site/about/guidelines.xhtml

Letters, abstracts, reviews, conference proceedings, and case reports were excluded as well as studies not written in English. We excluded studies with less than 100 patients and studies based on case scenarios because these studies have of a high risk of bias. Moreover, we excluded studies conducted in low or lower-income economies[12] because their unique characteristics, including the number of patients, epidemiology of diseases and available resources, make study results difficult to compare to most emergency departments in middle or higher income countries. Two reviewers (MV and NS or JZ and VH) independently assessed eligibility for inclusion. Disagreements in article selection were resolved through discussion.

Data extraction

Two reviewers (JZ and VH) independently extracted data from each of the included studies. A single study could consist of multiple “triage evaluations”, defined as the analysis of a single triage system in a single age group. Predefined age groups were 1) children; 2) adults or a combination of age groups; and 3) elderly. For each of the triage evaluations, the reviewers extracted the total number of included patients, the number of patients in each of the urgency categories of the triage system, the type of reference standard used, and the number of patients with a positive reference standard in each urgency category. If studies were based on overlapping data, we used the results from the most recent publication. For descriptive purposes we also collected data on study design and methods, patient demographics, and characteristics of the settings in which the study was performed.

Quality assessment

Two reviewers independently assessed quality of the selected articles using the QUADAS-2 (Quality Assessment of studies of Diagnostic Accuracy included in Systematic Reviews) checklist.[13] The QUADAS-2 evaluates 4 domains: patient selection, index test, reference standard, and flow and timing. Each domain is assessed in terms of risk of bias, and the first 3 domains also in terms of applicability. Because triage systems have some specific features as compared to other diagnostic tests, we adjusted the “reference standard domain” to make it applicable to our research question. We did not appraise whether the reference standard was interpreted without knowledge of the result of the index test, because this is unlikely when triage is applied in routine care. We did evaluate, however, whether data

For peer review only - http://bmjopen.bmj.com/site/about/guidelines.xhtml

For peer review only

on the outcome was collected blinded to the result of the index test. Moreover, we did not judge the applicability of the reference standard because there is no consensus on this topic.[14] Therefore, we included all studies with reference standard that were a proxy for patient urgency. Any disagreements between reviewers were resolved in a consensus meeting.

Data analysis

We used descriptive analyses to provide an overview of the available evidence on triage systems. Further analyses were restricted to triage systems that underwent at least 3 evaluations with the same reference standard. The primary outcome of our review was sensitivity and specificity of each of the triage systems for the identification of high and low urgency patients. Because there is no golden standard to determine "true" patient urgency, we a-priori selected 3 reference standards as proxy for patient urgency. We considered mortality at the ED and ICU admission after the ED visits as reference standard for high urgency, and discharge after the ED visit (i.e. patients not admitted to hospital) as reference standard for low urgency. Although these measures are not perfect, they approximate the desired outcome: most patients that die at the ED or require ICU admission are of high urgency, while most patients that are discharged after the ED visit are not. Moreover, these measures are suitable for the analyses of large datasets and commonly reported in research on triage systems.[14] In addition, we considered any other reference standard with sufficient evaluations in the same triage system.

We calculated two by two tables of triage system against the reference standard for each individual study. Because triage systems are ordinal scales, we dichotomized the urgency categories into a high urgency and low urgency group. High urgency was defined as triage urgency level 1 (3-level systems) or triage urgency level 1 and 2 (4-level and 5-level systems).

We calculated sensitivities and specificities, and presented the results as forest plots. For clinical practice and for benchmarking purposes, it may be interesting to know the proportion of patients with a positive reference standard in each of the triage urgency categories. Therefore, we additionally calculated the proportion of patients with a positive reference standard per urgency category, and displayed the results in a bar chart to enable comparison between studies and between triage systems.

We hypothesized that ED patient volume and case-mix severity of illness were determinants of triage systems' performance. Therefore we decided that if a sufficient number of studies were identified, we

For peer review only - http://bmjopen.bmj.com/site/about/guidelines.xhtml

would investigate the effect of these determinants on triage systems' performance using subgroup analyses. We considered annual ED census as a marker of patient volume and the percentage of hospitalized patients as a marker of case-mix severity of illness.

Computations were carried out with SPSS Statistics version 21.0 and figures were created using Review Manager 5.3 or R version 3.2.0.[15-17]

Patient and Public Involvement

Patients and public were not involved in this study.

For peer review only

For peer review only - http://bmjopen.bmj.com/site/about/guidelines.xhtml

Results

A total of 12,684 papers were identified in the electronic search, of which sixty-six were included in the final selection (figure 1).

The majority of studies were conducted in tertiary or university hospitals (n=46; 70%) and conducted in Europe/Central Asia or North America (n=45; 68%). 49 (74%) were single-centre studies. A complete overview of the selected studies is presented in appendix 2.

44 studies (67%) had a high risk of bias in at least one domain, and 17 studies (26%) had a high risk of bias in two or more domains (figure 2 and appendix 3). The most common causes of concern were application of multiple triage systems for the same patient by the same nurse or by multiple nurses without blinding, retrospective retrieval of reference standard information without blinding for the triage outcome, and substantial amounts of missing data. In 11 studies (17%) there were concerns regarding applicability.

Triage systems

In total, 89 individual triage evaluations were reported: 34 (38%) in children, 52 (58%) in adults, a combination of age groups or an unspecified population, and 3 (3%) in elderly. A total of 33 different triage systems were evaluated. The most commonly evaluated triage systems were the Emergency Severity Index (ESI) (n=22), the Manchester Triage System (MTS) (n=15), and the Canadian Triage and Acuity Scale (CTAS) (n=13). Other triage systems included the Taiwan Triage System (n=4), Australasian Triage Scale (n=3), South African Triage Scale (n=3), Netherlands Triage System (n=2), and Soterion Rapid Triage System (n=2). For 25 triage systems only one evaluation was published. These included 9 local or informally structured triage systems. The median sample size was 1,496 in children (range: 510 to 550,940), 1,447 in adults (range: 100 to 316,622), and 929 in elderly (range: 773 to 1,903).

Reference standards

A variety of reference standards were used and the majority of studies reported multiple reference standards (Appendix 4). 12 studies used mortality at the ED as a reference standard, 13 studies ICU admission and 47 studies hospital admission. Other commonly reported reference standards were length of stay at the ED (27 studies), resource use at the ED according to the ESI criteria (14 studies),

For peer review only - http://bmjopen.bmj.com/site/about/guidelines.xhtml

expert opinion (9 studies) and costs (8 studies). Because definitions of time to mortality, resource use and expert opinion were not consistent across studies, these results could not be compared. Moreover, length of stay at the ED and costs are outcome measures that are strongly dependent on ED characteristics, and could therefore not be used to make a comparison between studies.

The most commonly evaluated triage systems

Because we cannot interpret analyses of triage systems evaluated with different reference standards, we will further restrict our analyses to the triage systems with at least three evaluations using the same reference standard. This final selection includes studies evaluating the ESI, CTAS and MTS. Characteristics of these triage systems and a summary of the available evidence are presented in table 1. The ESI, CTAS and MTS were all evaluated in settings with a different patient volume as indicated by annual hospital census, and a different case-mix as indicated by percentage of hospitalisation. For each of these triage systems, the majority of studies had risk of bias in at least one domain.

Accuracy of triage systems to identify high urgent patients

Mortality at the ED was reported in 7 evaluations of our final sample: 5 evaluations in adults, and 2 in children. Because of this low number of studies and the very low reported mortality rates (on average 0.2% in adults and <0.01% in children) it was not possible to perform comparative analyses.

ICU admission was reported in 5 evaluations in adults (2 ESI, 3 MTS) and 4 in children (3 CTAS, 1 MTS). Overall, sensitivity for ICU admission was moderate to good, ranging from 0.58 (95%CI 0.48 to 0.68) to 0.88 (95%CI 0.70 to 0.96) in adults and 0.71 (95%CI 0.66 to 0.77) to 0.93 (95%CI 0.89 to 0.95) in children. A clear difference in performance between the triage systems was not visible (figure.3).

Regardless of the triage system used, most of the ICU admitted patients were allocated to one of the two highest triage categories (Appendix 5). The exact proportion of ICU admitted patients in each triage category was highly variable, even within studies evaluating the same triage system. For example, the proportion of ICU admitted adults in MTS category 1 ranged from 21% to 79%. The number of studies, however, was too small to assess whether this variability was present in all triage systems and whether this could be explained by study or setting related factors.

For peer review only - http://bmjopen.bmj.com/site/about/guidelines.xhtml

Accuracy to identify low urgent patients

Hospital admission or discharge after the ED visit was reported as a reference standard in 14 evaluations in adults and 15 in children. Overall, specificity of the triage systems to accurately classify patients discharged home as low urgent ranged from 0.64 (95%CI 0.62 to 0.66) to 0.98 (95%CI 0.95 to 0.99) in adults and 0.69 (95%CI 0.66 to 0.72) to 0.96 (95%CI 0.94 to 0.98) in children (Figure 4). Again, sensitivities and specificities were highly variable within each of the triage systems. None of the triage systems showed a marked better specificity compared to the others.

The proportion of patients discharged after the ED visit increased from the higher to the lower urgency categories in all triage systems (Appendix 5). Again, there was a large variability within triage systems and substantial overlap between triage systems. In adults, the MTS seemed to have a higher variability compared to the other triage systems, but in children variability was greater for the CTAS.

The only additional reference standard with sufficient evaluations was resource use according to the ESI criteria (Appendix 6).

Direct comparison of triage systems

A total of 13 studies directly compared two or more triage systems. Most of these studies, however, were assessed as having a high risk of bias in the index test domain, because triage was performed by the same nurse or without blinding. Performing triage while using different triage systems sequentially, is likely to reduce the differences between triage systems. Therefore, these results should be interpreted with caution.

In ten studies, an established five-level triage system was compared with a local or informally structured triage system with three or four levels. Seven of these studies reported that the 5-level triage system provided better discrimination or better sensitivities and specificities than the local triage system and should be preferred.[18-24] One study in children found that the local triage system performed better than the established triage system (CTAS).[25]

Two studies comparing the ESI with the MTS in adults found that sensitivities and specificities for hospital admission and the prediction of mortality were largely similar.[26 27] In one of these studies, the MTS undertriaged a smaller proportion of patients compared to the ESI (8.3% versus 13.5%) at the cost of a larger proportion of “overtriage”.[26] One study in adults observed no statistically significant

For peer review only - http://bmjopen.bmj.com/site/about/guidelines.xhtml

difference between the CTAS and ESI regarding the prediction of emergency department resource utilization and immediate patient outcomes.[28] One study in children compared the ATS and ESI and found similar sensitivities and specificities for the identification of patients requiring hospital admission.[29] One single-centre study in children compared five triage systems (ATS, CTAS, ESI, MTS and a local triage system called the Ramathibodi Triage System) and concluded that the ESI showed the best validity for predicting hospital admission (AUC 0.78, 95%CI 0.74–0.81).[30] In this study, the local triage system showed the highest sensitivity (50%) and the ATS the highest specificity (94%).

Determinants of triage systems' performance

The number of studies per triage system was rather small to perform subgroup analyses based on annual census or percentage hospitalisation. As an explorative analysis, we ordered all selected studies that used hospital admission as a reference standard based on annual census, and percentage hospitalisation (Appendix 7). There was no clear association between patient volume or case-mix and triage systems' sensitivity and specificity. A lower specificity for hospitals with the largest annual census and highest percentage hospitalisation could not be ruled out, but requires a larger number of studies.

For peer review only

For peer review only - http://bmjopen.bmj.com/site/about/guidelines.xhtml

Discussion

In a systematic review of sixty-six observational studies evaluating triage systems, we found that numerous different triage systems are being used but that many lack a rigorous evaluation. The most commonly used and evaluated triage systems, CTAS, ESI and MTS, show a moderate to good validity to identify high and low urgent patients. Their performance, however, is highly variable and differences in study design, study populations and reference standards make a comparison of the available evidence difficult. Although based on a limited number of studies, no clear association between patient volume and case-mix severity of illness could be found.

Strengths and weaknesses

This is the first study that evaluates the performance of triage systems in a meta-analysis. Previous reviews have merely described the results of the individual studies without synthesizing the evidence. Moreover, none of the published reviews looked at other factors that determine triage systems' performance, such as ED characteristics to compare evidence from different studies.[8-10 31-34]

Our review is based on a comprehensive search developed with a research librarian, includes duplicate assessment of eligibility and risk of bias, and duplicate data abstraction. Furthermore, the research question is based on a relevant and practical clinical issue. Triage systems are used worldwide to prioritize patients in the ED, but robust evidence on their performance is lacking.

The results of this review, however, should be interpreted taking into consideration the limitations of the underlying evidence. We included sixty-six studies in our review, but the majority of the thirty-three triage systems were evaluated by only one study. Therefore, we could only evaluate three most frequent used triage systems: CTAS, ESI and MTS. Even for these commonly used triage systems, few evaluations were available due to the variety of reference standards. Although the triage evaluations included the whole age spectrum, studies targeted at elderly patients were scarce. It is important to evaluate triage systems' performance separately for the most vulnerable populations at the ED, specifically children and elderly. In these patient groups the spectrum of disease, the presence of non-specific signs and symptoms and progression of disease course differs from that in adult patients.

We are not aware of any (randomized) controlled trial that investigates the effect of triage on patient outcome. Therefore we conducted a systematic review of observational studies. Comparing

For peer review only - http://bmjopen.bmj.com/site/about/guidelines.xhtml

observational studies is challenging because the effect of a triage system cannot be assessed independently of its context. Likely, other factors such as ED and hospital characteristics or local practices and training have influenced the results of the included studies. We chose to display the results of different studies in a forest plot. Because of the limited number of studies and heterogeneity of the study populations, it is difficult to compare the results from different triage systems and these plots should be interpreted with caution.

We aimed to explore heterogeneity between studies and more specifically the effect of differences in patient load and case-mix severity of illness. Unfortunately, due to the small number of studies per triage systems, we could not draw strong conclusions about the relation between these factors and triage systems' performance. There are more potential factors that could affect triage systems' validity, such as the local infrastructure, the experience and training of the triage nurse, the presence of a computerized triage application or variations in disease epidemiology. Moreover, since most included studies had risk of bias in at least one domain, we cannot rule out that study design and methodological quality have led to heterogeneity of the results as well.

We predefined mortality at the ED and ICU admission as reference standards for high patient urgency and discharge home as a reference standard for low patient urgency. Due to the relatively low number of studies reporting mortality and the low mortality rate of patients in the ED, we could not use it as a reference standard. ICU and hospital admission are feasible reference standards for large study populations, and theoretically, criteria for ICU and hospital admission should be reasonably comparable between settings. It is possible, however, that ED and hospital characteristics and local practices result in differences in the decision to admit a patient between EDs.

We restricted our review to triage systems in high and higher-middle income countries. We applied this selection because EDs in lower income countries have their own unique characteristics and challenges. Several recently published studies have addressed triage in low-income settings.[35 36]

Implications and future research

Our review identified thirty-tree triage systems for which at least one evaluation was published. Probably, there are more triage systems in use, which are not formally evaluated. There are several advantages, not addressed in this review, of using an established triage system over a local triage scale. Beside that performance of the most commonly used triage systems is known, they have a formal

For peer review only - http://bmjopen.bmj.com/site/about/guidelines.xhtml

governance structure and undergo regular updates. Moreover, there are standardized implementation guidelines and training programs available.[37-39] The CTAS, ESI and MTS all show a reasonable performance for triage at the ED. Our results do not suggest that one of the established triage systems should be preferred over the other.

Our review indicates that large variation of performance exists even in studies assessing the same triage system. This suggests that other factors influence triage systems' performance. Consequently, generalizability of individual studies evaluating a triage system is low and a study on triage validity in one setting may not apply to a setting with different characteristics or in a different health care system. Our review demonstrates that the majority of studies evaluating triage systems were conducted in a single-centre. Furthermore, most multicentre studies provided only pooled results. Yet, multicentre studies using similar study designs and reference standard definitions are needed to evaluate the range of triage systems' performance in different settings. More importantly, these studies can provide valuable insights in determinants of triage systems' performance and areas of improvement.

The extensive use of triage systems in clinical practice contrasts with the limited number of studies evaluating their performance. Triage systems were typically developed based on expert opinion and implemented out of clinical necessity. They are mostly used in their country or region of origin: the MTS is widely used in the United Kingdom and Europe, the ESI in the United States, and the CTAS in Canada and in French-speaking countries.[40] Since most EDs already have experience with a certain triage system and some triage systems are recommended by national guidelines it could be worthwhile shifting away the focus on triage systems' performance towards improvement of the established triage systems. Our review suggests that there is room for improvement of all triage systems regarding both the correct identification of high urgent and low urgent patients.

The most commonly used triage systems, CTAS, ESI and MTS, have a reasonable validity for the triage of patients at the ED. Important research questions that remain are what determinants influence a triage systems' performance and how the performance of existing triage systems can be improved.

Acknowledgements

We thank Wichor Bramer, biomedical information specialist in Erasmus University Medical Centre, Rotterdam, for his help with developing the search strategy

For peer review only - http://bmjopen.bmj.com/site/about/guidelines.xhtml

Authors' contributions

JMZ and VvdH contributed equally to this work. All authors substantially contributed to the conception and design of the study and interpretation of the findings. JMZ, KMJ and HAM conceived the study idea. JMZ, VvdH, NS, MvV conducted the systematic review including screening of abstracts and full text and risk of bias assessment. JMZ and VvdH extracted data, performed the analyses and wrote the first draft of the manuscript. All authors revised it critically for important intellectual content and gave their approval of the final version. All authors had full access to all of the data (including statistical reports and tables) in the study and can take responsibility for the integrity of the data and the accuracy of the data analysis. HAM is guarantor.

For peer review only

For peer review only - http://bmjopen.bmj.com/site/about/guidelines.xhtml

References

1. Blunt I, Edwards N, Merry L. What's behind the A&E 'crisis'? *Briefing* 2015.
2. Australasian College for Emergency Medicine. Access Block and Overcrowding in Emergency departments. , 2004.
3. Hospital-based emergency care: at the breaking point. ; 2013.
4. Mackway-Jones K, Marsden J, Windle J. Emergency triage. London: BMJ 2006.
5. Warren DW, Jarvis A, LeBlanc L, et al. Revisions to the Canadian Triage and Acuity Scale paediatric guidelines (PaedCTAS). *Cjem* 2008;10(3):224-43.
6. Gilboy NT, P.; Travers, D.A.; Rosenau, A.M.; Eitel, D.R. Emergency Severity Index, Version 4: Implementation Handbook. Rockville, MD: Agency for Healthcare Research and Quality 2005.
7. Australian College for Emergency Medicine. Guidelines on the implementation of the australasian triage scale in emergency departments. http://www.acem.org.au/media/policies_and_guidelines/G24_Implementation__ATS.pdf [
8. Christ M, Grossmann F, Winter D, et al. Modern triage in the emergency department. *Dtsch Arztebl* 2010;107(50):892-98.
9. Farrohknia N, Castren M, Ehrenberg A, et al. Emergency department triage scales and their components: a systematic review of the scientific evidence. *Scand J Trauma Resusc Emerg Med* 2011;19:42.
10. Parenti N, Ferrara L, Bacchi Reggiani ML, et al. Reliability and validity of two four-level emergency triage systems. *Eur J Emerg Med* 2009;16(3):115-20.
11. Stroup DF, Berlin JA, Morton SC, et al. Meta-analysis of observational studies in epidemiology: a proposal for reporting. Meta-analysis Of Observational Studies in Epidemiology (MOOSE) group. *Jama* 2000;283(15):2008-12.
12. World Bank Group. World Bank Country and Lending Groups. https://datahelpdesk.worldbank.org/knowledgebase/articles/906519-world-bank-country-and-lending-groups. Accessed October 5th, 2017.
13. Whiting PF, Rutjes AW, Westwood ME, et al. QUADAS-2: a revised tool for the quality assessment of diagnostic accuracy studies. *Ann Intern Med* 2011;155(8):529-36.
14. Kuriyama A, Urushidani S, Nakayama T. Five-level emergency triage systems: variation in assessment of validity. *Emerg Med J* 2017
15. R: A language and environment for statistical computing [program]. Vienna, Austria: R Foundation for Statistical Computing, 2008.
16. IBM SPSS Statistics for Windows [program]. 21 version. Armonk, NY 2011.
17. Review Manager (RevMan) [program]. 5.3 version. Copenhagen: The Nordic Cochrane Centre, The Cochrane Collaboration, 2014.
18. Chang YC, Ng CJ, Wu CT, et al. Effectiveness of a five-level Paediatric Triage System: An analysis of resource utilisation in the emergency department in Taiwan. *Emerg Med J* 2012
19. Chi CH, Huang CM. Comparison of the Emergency Severity Index (ESI) and the Taiwan triage system in predicting resource utilization. *J Formos Med Assoc* 2006;105(8):617-25.

For peer review only - http://bmjopen.bmj.com/site/about/guidelines.xhtml

20. Maleki M, Fallah R, Riahi L, et al. Effectiveness of Five-Level Emergency Severity Index Triage System Compared With Three-Level Spot Check: An Iranian Experience. *Arch Trauma Res* 2015;4(4):e29214.
21. Ng CJ, Hsu KH, Kuan JT, et al. Comparison between Canadian Triage and Acuity Scale and Taiwan Triage System in emergency departments. *J Formos Med Assoc* 2010;109(11):828-37.
22. Ng CJ, Yen ZS, Tsai JCH, et al. Validation of the Taiwan triage and acuity scale: A new computerised five-level triage system. *Emerg Med J* 2011;28(12):1026-31.
23. Yuksen C, Sawatmongkornkul S, Suttabuth S, et al. Emergency severity index compared with 4-level triage at the emergency department of Ramathibodi University Hospital. *Asian Biomed* 2016;10(2):155-61. doi: 10.5372/1905-7415.1002.477
24. Travers DA, Waller AE, Bowling JM, et al. Five-level triage system more effective than three-level in tertiary emergency department. *J Emerg Nurs* 2002;28(5):395-400.
25. Gouin S, Gravel J, Amre DK, et al. Evaluation of the Paediatric Canadian Triage and Acuity Scale in a pediatric ED. *Am J Emerg Med* 2005;23(3):243-47.
26. Storm-Versloot MN, Ubbink DT, Kappelhof J, et al. Comparison of an informally structured triage system, the emergency severity index, and the manchester triage system to distinguish patient priority in the emergency department. *Acad Emerg Med* 2011;18(8):822-29.
27. van der Wulp I, Schrijvers AJP, van Stel HF. Predicting admission and mortality with the Emergency Severity Index and the Manchester Triage System: a retrospective observational study. *Emergency Medicine Journal* 2009;26(7):506-09.
28. Worster A, Fernandes CM, Eva K, et al. Predictive validity comparison of two five-level triage acuity scales. *Eur J Emerg Med* 2007;14(4):188-92.
29. Ghafarypour-Jahrom M, Taghizadeh M, Heidari K, et al. Validity and Reliability of the Emergency Severity Index and Australasian Triage System in Pediatric Emergency Care of Mofid Children's Hospital in Iran. *Bull Emerg Trauma* 2018;6(4):329-33.
30. Aeimchanbanjong K, Pandee U. Validation of different pediatric triage systems in the emergency department. *World J Emerg Med* 2017;8(3):223-27.
31. Azeredo TR, Guedes HM, Rebelo de Almeida RA, et al. Efficacy of the Manchester Triage System: a systematic review. *Int Emerg Nurs* 2015;23(2):47-52.
32. Souza CC, Araujo FA, Chianca TC. [Scientific Literature on the Reliability and Validity of the Manchester Triage System (MTS) Protocol: A Integrative Literature Review]
Producao cientifica sobre a validade e confiabilidade do Protocolo de Manchester: revisao integrativa da literature. *Rev Esc Enferm USP* 2015;49(1):144-51.
33. van Veen M, Moll HA. Reliability and validity of triage systems in paediatric emergency care. *Scand J Trauma Resusc Emerg Med* 2009;17:38.
34. de Magalhaes-Barbosa MC, Robaina JR, Prata-Barbosa A, et al. Validity of triage systems for paediatric emergency care: a systematic review. *Emerg Med J* 2017
35. Hansoti B, Jenson A, Keefe D, et al. Reliability and validity of pediatric triage tools evaluated in Low resource settings: a systematic review. *BMC Pediatr* 2017;17(1):37.
36. Jenson A, Hansoti B, Rothman R, et al. Reliability and validity of emergency department triage tools in low- and middle-income countries: a systematic review. *Eur J Emerg Med* 2018;25(3):154-60.
37. www.esitriage.com. Accessed October 5th, 2017. .

For peer review only - http://bmjopen.bmj.com/site/about/guidelines.xhtml

38. http://caep.ca/resources/ctas. Accessed October 5th, 2017.
39. www.triagenet.net. Accessed October 5th, 2017. [accessed October 5th 2017.
40. Moll HA. Challenges in the validation of triage systems at emergency departments. *J Clin Epidemiol* 2010;63(4):384-88.

For peer review only

For peer review only - http://bmjopen.bmj.com/site/about/guidelines.xhtml

Tables and Figure captions

Table 1. Evidence summary of the most commonly used triage systems

	**Canadian Triage And Acuity Scale (CTAS)**	**Emergency Severity Index (ESI)**	**Manchester Triage System (MTS)**
Triage system characteristics			
Description	List, based on presenting signs and symptoms	Flowchart, based on physical signs and expected resource use	Multiple flowcharts, based on presentational signs and symptoms
Number of levels	5	5	5
Classification and waiting time	Level I, Immediate<br>Level II, 15 min<br>Level III, 30 min<br>Level IV, 60 min<br>Level V, 2 hrs	Immediate<br>Emergent, 14 min<br>Urgent, 60 min<br>Semi-urgent, 2 hrs<br>Non-urgent, 24 hrs	Immediate<br>Very urgent, 10 min<br>Urgent, 60 min<br>Standard, 2 hrs<br>Non-urgent, 4 hrs
Quantity of evidence			
Total number of evaluations	13	21	15
Evaluations in children	9	4	7
Evaluations in elderly	1	2	0
Diversity of evidence (range)			
Number of hospitals per study	1-12	1-7	1-4
Inclusions per study	481-550,940	180-37,974	872-31,622
Hospital census	10,000-75,000	10,000-90,000	7,000-190,000
Hospitalisation rate	8%-47%	10%-62%	5-33%
Risk of bias			
High risk of bias in at least one domain	54%	81%	67%
High risk of bias in >1 domain	15%	23%	13%

For peer review only - http://bmjopen.bmj.com/site/about/guidelines.xhtml

Figure 1. Flow diagram of study selection process

Figure 2. Risk of bias of included studies

Figure 3. Sensitivity and specificity of triage systems for identifying high urgency patients as defined by ICU admission

CTAS = Canadian Triage and Acuity Scale; TP = true positive; FP = false positive; FN = false negative; TN = true negative; ESI = Emergency Severity Index; MTS = Manchester Triage System

Figure 4. Sensitivity and specificity of triage systems for identifying low urgency patients as defined by discharge home after the ED visit

CTAS = Canadian Triage and Acuity Scale; TP = true positive; FP = false positive; FN = false negative; TN = true negative; ESI = Emergency Severity Index; MTS = Manchester Triage System

For peer review only

For peer review only - http://bmjopen.bmj.com/site/about/guidelines.xhtml

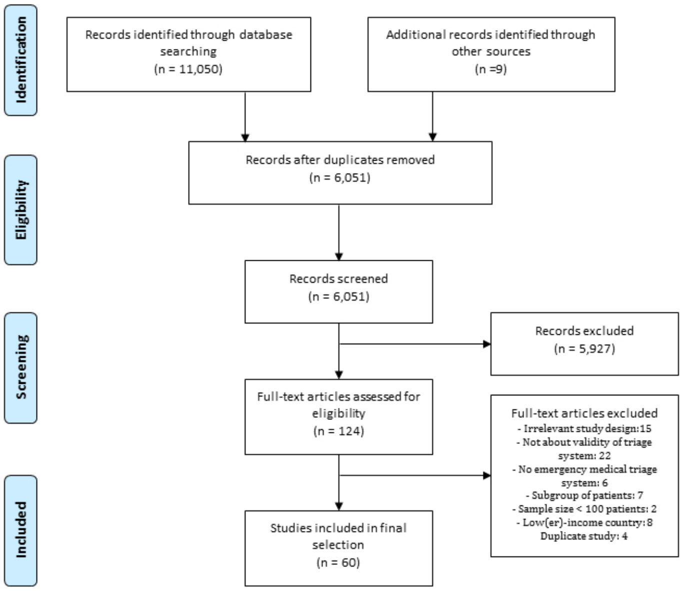

Figure 1. Flow diagram of study selection process

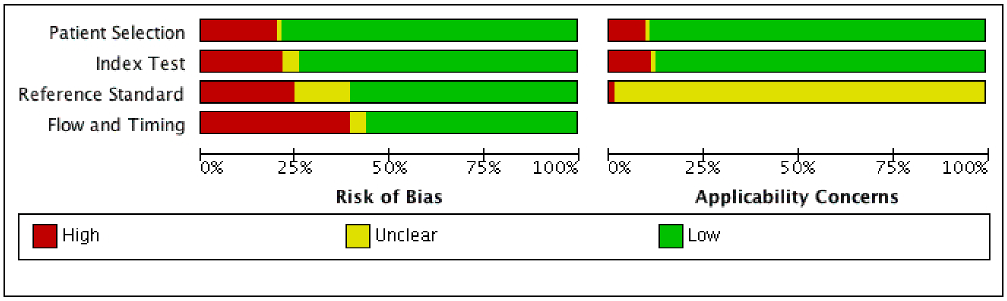

Figure 2. Risk of bias of included studies

241x70mm (72 x 72 DPI)

CTAS

Study	TP	FP	FN	TN	Age group	Sensitivity (95% CI)	Specificity (95% CI)
Gravel 2009	268	4415	21	53825	Children	0.93 [0.89, 0.95]	0.92 [0.92, 0.93]
Gravel 2013	1646	64284	434	482987	Children	0.79 [0.77, 0.81]	0.88 [0.88, 0.88]
Allon 2017	264	4697	128	78520	Children	0.67 [0.62, 0.72]	0.94 [0.94, 0.95]

ESI

Study	TP	FP	FN	TN	Age group	Sensitivity (95% CI)	Specificity (95% CI)
Tanabe 2004	22	155	3	223	Adults / Unspecified	0.88 [0.69, 0.97]	0.59 [0.54, 0.64]
Yuksen 2016	15	91	3	411	Adults / Unspecified	0.83 [0.59, 0.96]	0.82 [0.78, 0.85]

MTS

Study	TP	FP	FN	TN	Age group	Sensitivity (95% CI)	Specificity (95% CI)
Zachariasse 2016	208	11323	83	54447	Children	0.71 [0.66, 0.77]	0.83 [0.82, 0.83]
Graff 2014	1888	6741	422	36418	Adults / Unspecified	0.82 [0.80, 0.83]	0.84 [0.84, 0.85]
Steiner 2016	54	588	39	1726	Adults / Unspecified	0.58 [0.47, 0.68]	0.75 [0.73, 0.76]
Zachariasse 2017	836	34642	166	186925	Adults / Unspecified	0.83 [0.81, 0.86]	0.84 [0.84, 0.85]

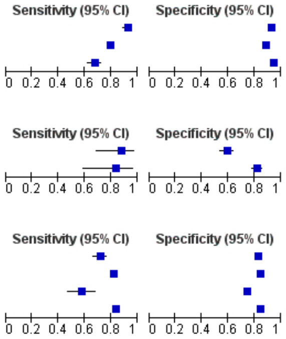

Figure 3. Sensitivity and specificity of triage systems for identifying high urgency patients as defined by ICU admission
CTAS = Canadian Triage and Acuity Scale; TP = true positive; FP = false positive; FN = false negative; TN = true negative; ESI = Emergency Severity Index; MTS = Manchester Triage System

320x136mm (72 x 72 DPI)

CTAS

Study	TP	FP	FN	TN	Age group	Sensitivity (95% CI)	Specificity (95% CI)
Gouin 2005	8	19	38	472	Children	0.17 [0.08, 0.31]	0.96 [0.94, 0.98]
Gravel 2009	1937	2746	3124	50722	Children	0.38 [0.37, 0.40]	0.95 [0.95, 0.95]
Gravel 2012	30	69	69	1296	Children	0.30 [0.21, 0.40]	0.95 [0.94, 0.96]
Gravel 2013	20876	45054	25093	458328	Children	0.45 [0.45, 0.46]	0.91 [0.91, 0.91]
Chang 2013	3104	6821	6276	26327	Children	0.33 [0.32, 0.34]	0.79 [0.79, 0.80]
Al-Hindi 2014	65	295	45	2609	Children	0.59 [0.49, 0.68]	0.90 [0.89, 0.91]
Aeimchanbanjong 2017	62	239	62	679	Children	0.50 [0.41, 0.59]	0.74 [0.71, 0.77]
Allon 2017	2631	2330	17726	60922	Children	0.13 [0.12, 0.13]	0.96 [0.96, 0.96]
Dong 2007	1519	1947	4189	21869	Adults / Unspecified	0.27 [0.25, 0.28]	0.92 [0.91, 0.92]
Ng 2010	271	244	350	986	Adults / Unspecified	0.44 [0.40, 0.48]	0.80 [0.78, 0.82]

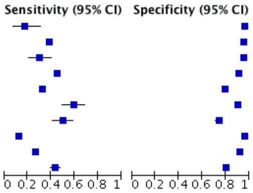

ESI

Study	TP	FP	FN	TN	Age group	Sensitivity (95% CI)	Specificity (95% CI)
Baumann 2005	44	66	28	372	Children	0.61 [0.49, 0.72]	0.85 [0.81, 0.88]
Travers 2009	276	168	50	679	Children	0.85 [0.80, 0.88]	0.80 [0.77, 0.83]
Green 2012	33	42	47	658	Children	0.41 [0.30, 0.53]	0.94 [0.92, 0.96]
Aeimchanbanjong 2017	64	165	59	753	Children	0.52 [0.43, 0.61]	0.82 [0.79, 0.84]
Wuerz 2000	91	53	68	281	Adults / Unspecified	0.57 [0.49, 0.65]	0.84 [0.80, 0.88]
Wuerz 2001	1113	674	1260	5204	Adults / Unspecified	0.47 [0.45, 0.49]	0.89 [0.88, 0.89]
Tanabe 2004	129	48	75	151	Adults / Unspecified	0.63 [0.56, 0.70]	0.76 [0.69, 0.82]
Chi 2006	713	747	376	1336	Adults / Unspecified	0.65 [0.63, 0.68]	0.64 [0.62, 0.66]
Van der Wulp 2009	2259	2146	5911	27658	Adults / Unspecified	0.28 [0.27, 0.29]	0.93 [0.93, 0.93]
Storm-Versloot 2011	66	76	96	638	Adults / Unspecified	0.41 [0.33, 0.49]	0.89 [0.87, 0.92]
Yuksen 2016	38	68	22	392	Adults / Unspecified	0.63 [0.50, 0.75]	0.85 [0.82, 0.88]
Fong 2018	6	5	68	221	Adults / Unspecified	0.08 [0.03, 0.17]	0.98 [0.95, 0.99]

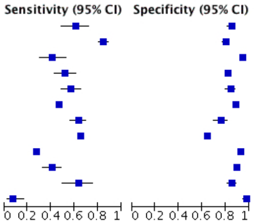

MTS

Study	TP	FP	FN	TN	Age group	Sensitivity (95% CI)	Specificity (95% CI)
Roukema 2006	147	256	62	600	Children	0.70 [0.64, 0.76]	0.70 [0.67, 0.73]
Seiger 2014	2992	7410	3903	46430	Children	0.43 [0.42, 0.45]	0.86 [0.86, 0.87]
Aeimchanbanjong 2017	70	285	53	633	Children	0.57 [0.48, 0.66]	0.69 [0.66, 0.72]
Van der Wulp 2009	2641	3183	4415	24019	Adults / Unspecified	0.37 [0.36, 0.39]	0.88 [0.88, 0.89]
Martins 2009	18265	63803	12464	222090	Adults / Unspecified	0.59 [0.59, 0.60]	0.78 [0.78, 0.78]
Storm-Versloot 2011	73	72	76	651	Adults / Unspecified	0.49 [0.41, 0.57]	0.90 [0.88, 0.92]
Santos 2013	485	3080	535	19515	Adults / Unspecified	0.48 [0.44, 0.51]	0.86 [0.86, 0.87]
Graff 2014	6497	2132	8463	28377	Adults / Unspecified	0.43 [0.43, 0.44]	0.93 [0.93, 0.93]

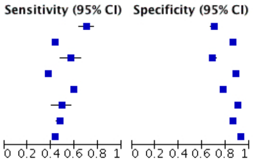

Figure 4. Sensitivity and specificity of triage systems for identifying low urgency patients as defined by discharge home after the ED visit

CTAS = Canadian Triage and Acuity Scale; TP = true positive; FP = false positive; FN = false negative; TN = true negative; ESI = Emergency Severity Index; MTS = Manchester Triage System

359x255mm (72 x 72 DPI)

Appendix 1. Search strategy

Embase

(triage* OR ((acuity OR severit* OR priorit* OR critical*) NEAR/3 (scale* OR level* OR index* OR score* OR measure* OR assessment*))):ab,ti AND ('emergency ward'/de OR 'emergency medicine'/de OR 'emergency care'/exp OR (((emergen* OR acute) NEAR/3 (department* OR ward* OR unit* OR room* OR care))):ab,ti) AND (reliability/exp OR reproducibility/de OR 'validation process'/de OR 'validation study'/de OR accuracy/de OR 'sensitivity and specificity'/exp OR 'diagnostic accuracy'/de OR evaluation/de OR validity/exp OR (reliab* OR reproducib* OR validation OR validaty OR consisten* OR variabilit* OR accura* OR 'intra observer' OR intraobserver OR sensitivity OR specificity):ab,ti OR evaluat*:ti) AND [english]/lim

Medline Ovid

(Triage/ OR (triage* OR ((acuity OR severit* OR priorit* OR critical*) ADJ3 (scale* OR level* OR index* OR score* OR measure* OR assessment*))).ab,ti.) AND (exp "Emergency Service, Hospital"/ OR "emergency medicine"/ OR "emergency care"/ OR (((emergen* OR acute) ADJ3 (department* OR ward* OR unit* OR room* OR care))).ab,ti.) AND (Reproducibility of Results/ OR Validation Studies.pt. OR exp "sensitivity and specificity"/ OR Evaluation Studies.pt. OR (reliab* OR reproducib* OR validation OR validaty OR consisten* OR variabilit* OR accura* OR "intra observer" OR intraobserver OR sensitivity OR specificity).ab,ti. OR evaluat*.ti.) AND english.la.

Cochrane central

(triage* OR ((acuity OR severit* OR priorit* OR critical*) NEAR/3 (scale* OR level* OR index* OR score* OR measure* OR assessment*))):ab,ti AND ((((emergen* OR acute) NEAR/3 (department* OR ward* OR unit* OR room* OR care))):ab,ti) AND ((reliab* OR reproducib* OR validation OR validaty OR consisten* OR variabilit* OR accura* OR 'intra observer' OR intraobserver OR sensitivity OR specificity):ab,ti OR evaluat*:ti)

Web-of-science

TS=(triage* OR ((acuity OR severit* OR priorit* OR critical*) NEAR/3 (scale* OR level* OR index* OR score* OR measure* OR assessment*))) AND TS=((((emergen* OR acute) NEAR/3 (department* OR ward* OR unit* OR room* OR care)))) AND (TS=(reliab* OR reproducib* OR validation OR validaty OR consisten* OR variabilit* OR accura* OR "intra observer" OR intraobserver OR sensitivity OR specificity) OR TI=(evaluat*)) AND LA=(English)

CINAHL

(MH Triage+ OR (triage* OR ((acuity OR severit* OR priorit* OR critical*) N3 (scale* OR level* OR index* OR score* OR measure* OR assessment*)))) AND (MH "Emergency Service"+ OR MH "emergency medicine"+ OR (((emergen* OR acute) N3 (department* OR ward* OR unit* OR room* OR care)))) AND (MH Reproducibility of Results+ OR MH Validity+ OR MH "sensitivity and specificity"+ OR MH Evaluation+ OR (reliab* OR reproducib* OR validation OR validaty OR consisten* OR variabilit* OR accura* OR "intra observer" OR intraobserver OR sensitivity OR specificity) OR TI (evaluat*))

Google Scholar

triage emergency|emergencies
reliability|reproducibility|validation|validity|consistency|accuracy|sensitivity|specificity|interobserver |intraobserver

For peer review only

Appendix 2. Selected studies

Table 2.1 Characteristics of included studies

Study	Setting	Annual census	Hospitalization rate	Study population	Triage System	Reference standard (data available)
Aeimchanbanjong, 2017[1]	Single academic hospital, Thailand	10,000	12%	1041 children	ATS, CTS, ESI, MTS, Ramathibodi Triage System	- Hospital admission
Al-Hindi, 2014[2]	Single tertiary maternity and children's hospital, Saudi Arabia	155,000	4%	3,014 children	Ped-CTAS	- Hospital admission<br>- Resource use, other<br>- Waiting time to physician
Allon, 2017[3]	Single tertiary children's hospital, Israel	-	24%	83,609 children	Ped-CTAS	- Hospital admission<br>- ICU admission<br>- Length of stay at the ED<br>- Left without being seen
Baumann, 2005[4]	Single academic hospital, USA	53,000	14%	510 children	ESI v.3	- Hospital admission<br>- Resource use based on ESI<br>- Length of stay at the ED<br>- Mortality at the ED<br>- Mortality after 1 year<br>- Left without being seen
Baumann, 2006[5]	Single academic hospital, USA	53,000	43%	929 elderly	ESI v.3	- Hospital admission<br>- ICU admission<br>- Resource use based on ESI<br>- Length of stay at the ED<br>- Mortality at the ED<br>- Mortality after 1 year<br>- Left without being seen
Brillman, 1996[6]	Single academic hospital,	65,000	6%	5,106 patients	Automated Military	- Hospital admission<br>- Expert opinion

	USA				Outpatient System	
Bruijns, 2008[7]	Single community hospital, South Africa	54,000	40%	798 adults	Cape Triage Score	- Hospital admission<br>- Mortality at the ED
Chang, 2012[8]	Single academic hospital, Taiwan	-	23%	42,346 children	CTAS and Ped-TTS	- Hospital admission<br>- Costs
Chi, 2006[9]	Single academic hospital, Taiwan	58,000	34%	3,172 adults	ESI v.2 and TTS	- Hospital admission<br>- Length of stay at the ED
Dong, 2007[10]	Single tertiary hospital, Canada	67,000	19%	29,524 adults	CTAS	- Hospital admission<br>- Resource use, other<br>- Length of stay at the ED<br>- Mortality at the ED<br>- Costs
Dugas, 2016[11]	Sample of ED visits from 356 hospitals, USA	-	14%	25,198 adults	Combination of triage systems and Electronic emergency Triage System	- Hospital admission<br>- ICU admission<br>- Resource use, other<br>- Resource use based on ESI<br>- Composite outcome: in-hospital mortality, intensive care unit admission or transfer to operating room or catheterization suite
Eitel, 2003[12]	7 community hospitals, USA	28,000-90,000	34% (mean)	1,042 patients	ESI v.2	- Hospital admission<br>- Resource use based on ESI<br>- Length of stay at the ED<br>- Mortality: 60-day mortality
Engan, 2018[13]	Single academic hospital, Norway	4,750	44%	782 children	modified version of the pediatric SATS	- Hospital admission<br>- Resource use, other
Erimsah, 2015[14]	Single academic hospital,	40,000	16%	618 patients	Ministry of Health of	- Hospital admission<br>- Resource use, other

For peer review only

	Turkey				Turkey's mandatory emergency triage instrument	- Length of stay at the ED<br>- Expert opinion<br>- Immediate lifesaving interventions<br>- Mortality: in-hospital mortality
Fan, 2013[15]	Single tertiary hospital,<br>China (Hong Kong)	128,000	-	100 children and adults	Hong Kong Accident and Emergency Triage Guidelines 4th version	- Expert opinion
Fong, 2018[16]	Single community hospital, Singapore	-	25%	300 adults	ESI and Patient Acuity Category Scale	- Hospital admission<br>- Resource use based on ESI
Funakoshi, 2016[17]	Single community hospital,<br>Japan	31,793	-	17,121 adults	Japanese Triage and Acuity Scale	- Hospital admission<br>- Length of stay at the ED<br>- Mortality at the ED
Ganapathy, 2018[18]	Single community hospital, Singapore	-	17%	172,933 children	Singapore Paediatric Triage Scale	- Hospital admission<br>- ICU admission<br>- Length of stay at the ED<br>- Length of stay in hospital
Van Gerven, 2001[19]	Single academic hospital,<br>Belgium	-	45%	3,650 children and adults	Australian National Triage Scale	- Hospital admission<br>- Sentinel diagnosis
Ghafarypour-Jahrom, 2018[20]	Single academic hospital, Iran	45,000	-	1,300 children	ATS and ESI	- Hospital admission
Gouin, 2005[21]	Single academic children's hospital,<br>Canada	65,000	9%	1,281 children	CTAS and local triage tool	- Hospital admission<br>- Resource use, other<br>- PRISA score
Gräff, 2014[22]	Single academic hospital,<br>Germany	23,000	33%	45,469 children and adults	MTS	- Hospital admission<br>- ICU admission<br>- Resource use, other

For peer review only

						- Length of stay at the ED<br>- Mortality: in-hospital mortality<br>- Length of stay in hospital<br>- Level of prehospital care
Gravel, 2009[23]	Single academic hospital, Canada	60,000	9%	5,829 children	Ped-CTAS	- Hospital admission<br>- ICU admission<br>- Length of stay at the ED<br>- Mortality at the ED
Gravel, 2012[24]	9 academic hospitals, Canada	43,962	8%	1,464 children	CTAS	- Hospital admission<br>- Resource use, other<br>- Length of stay at the ED
Gravel, 2013[25]	12 academic children's hospitals, Canada	25,000-75000	9%	550,940 children	CTAS	- Hospital admission<br>- ICU admission<br>- Length of stay at the ED<br>- Left without being seen
Green, 2012[26]	Single academic children's hospital, USA	45,000	10%	780 children	ESI v.4	- Hospital admission<br>- Resource use based on ESI<br>- Length of stay at the ED
Hansen, 2017[27]	Single hospital, Denmark	-	-	550 children	Danish Regions Pediatric Triage model	- Composite outcome "van Veen"; based on abnormal vital parameters, life-threatening conditions, diagnostics, interventions and follow-up<br>- Composite outcome based on life threatening condition, transfer to higher hospital, ICU, or hospital admission
Hay, 2001[28]	Single hospital, Israel	-	-	2,886 patients	Local triage system	- Expert opinion<br>- Waiting time to physician
Van Ierland, 2011[29]	Single academic hospital, the Netherlands	20,000	30% (adults) and 15% (children)	3,207 adults and 701 children	Netherlands Triage System	- Hospital admission<br>- ICU admission<br>- Resource use, other<br>- Follow-up at outpatient clinic or GP

For peer review only

Jobe, 2013[30]	Single academic hospital, Belgium	42,000	36%	544 children and adults	Echelle Liégeoise d'Index de Sévérité à l'Admission	- Hospital admission<br>- ICU admission<br>- Resource use, other<br>- Resource use based on ESI<br>- Mortality at the ED
Lee, 2003[31]	4 hospitals, China (Hong Kong)	-	-	2,410 patients	5-level triage scale	- Expert opinion
Lee, 2011[32]	Single academic hospital, South Korea	60,000	47%	1,903 elderly	CTAS, revised edition	- Hospital admission<br>- ICU admission<br>- Resource use, other<br>- Mortality at the ED<br>- Costs<br>- Immediate lifesaving interventions<br>- Mortality: in-hospital mortality<br>- Length of stay in hospital
Lin, 2016[33]	Single academic hospital, China	217,000	-	275,378 children	Chinese pediatric emergency triage system	- Expert opinion<br>- Waiting time for care<br>- Patients' parents' satisfaction
Ma, 2008[34]	Single academic children's hospital, Canada	50,000	-	1,618 children	Ped-CTAS	- Costs
Maleki, 2015[35]	Single teaching hospital, Iran	74,000	-	387 patients	ESI v.4 and Spot check triage system	- Waiting time to nurse<br>- Waiting time to physician<br>- Waiting time to first lab results
Maningas, 2006[36]	Single community hospital, USA	57,000	6%	7,077 children	Soterion Rapid Triage System	- Hospital admission<br>- Length of stay at the ED<br>- Costs<br>- CPT codes
Maningas, 2006[37]	Single community hospital, USA	57,000	10%	33,850 adults	Soterion Rapid Triage System	- Hospital admission<br>- Length of stay at the ED<br>- Costs<br>- CPT codes

For peer review only

Martins, 2009[38]	Single community hospital, Portugal	128,000	10%	316,622 adults	MTS	- Hospital admission<br>- Mortality at the ED
Meyer, 2018[39]	Single community hospital, South Africa	-	22%	4,002 patients	SATS	- Hospital admission
Ng, 2010[40]	3 hospitals, Taiwan	-	34%	1,851 adults	CTAS and Taiwan Triage System	- Hospital admission<br>- Length of stay at the ED<br>- Costs
Ng, 2011[41]	11 academic hospitals, 18 regional hospitals and 4 district hospitals, Taiwan	95,912 (average, university hospitals); 47,056 (average regional hospitals); 23,789 (average district hospitals	30%	10,533 adults	Taiwan Triage and Acuity Scale and Taiwan Triage System	- Hospital admission<br>- Resource use based on ESI<br>- Length of stay at the ED
Platts-Mills, 2010[42]	Single academic hospital, USA	64,000	58%	773 elderly	ESI v.4	- Immediate lifesaving interventions
Roukema, 2006[43]	Single teaching hospital, the Netherlands	30,000	-	1,065 children	MTS	- Hospital admission<br>- Resource use based on ESI<br>- Composite outcome “van Veen”; based on abnormal vital parameters, life-threatening conditions, diagnostics, interventions and follow-up
Santos, 2013[44]	Single hospital, Portugal	-	5%	24,721 adults	MTS v.2	- Hospital admission<br>- Resource use, other<br>- Mortality: in-hospital mortality

For peer review only

Seiger, 2014[45]	2 academic and 2 teaching hospitals, the Netherlands, UK and Portugal	9000-60,000	11% (average)	60,735 children	MTS	- Hospital admission
Steiner, 2016[46]	Single tertiary hospital, Switzerland	-	-	2,407 adults	MTS	- ICU admission<br>- Expert opinion<br>- Mortality: 30 day mortality<br>- Length of stay in hospital
Storm-Versloot, 2011[47]	Single academic hospital, the Netherlands	31,000	18%	876 patients	ESI, MTS and informally structured triage system	- Hospital admission<br>- Resource use based on ESI<br>- Length of stay at the ED<br>- Expert opinion
Storm- Versloot, 2014[48]	Single academic hospital, the Netherlands	31,000	21%	906 patients	MTS and informally structured triage system	- Length of stay at the ED<br>- Waiting time from arrival to treatment room<br>- Waiting time from treatment room to discharge from ED<br>- Patient satisfaction
Taboulet, 2009[49]	Single teaching hospital, France	30,000	18%	941 adults	FRench Emergency Nurses Classification in Hospital Scale v.2	- Hospital admission<br>- Resource use, other<br>- Waiting time to physician
Tanabe, 2004[50]	Single academic hospital, USA	70,000	-	403 children and adults	ESI v.3	- Resource use, other<br>- Length of stay at the ED<br>- Length of stay in hospital
Tanabe, 2004[51]	Single academic hospital, USA	70,000	-	403 children and adults	ESI v.3	- Hospital admission<br>- Resource use based on ESI<br>- Length of stay at the ED
Travers, 2002[52]	Single academic hospital, USA	-	-	305 patients	ESI and 3-level system	- Expert opinion
Travers, 2009[53]	1 academic hospital	13,838-	-	1,173	ESI v.4	- Hospital admission

For peer review only

	and 4 teaching/community hospitals, USA	44,987		children		- Resource use based on ESI<br>- Length of stay at the ED
Twomey, 2013[54]	1 private and 5 public community hospitals, South Africa	-	26%	2,014 children	revised paediatric SATS	- Hospital admission
Veen, van, 2008[55]	1 academic children's and 1 teaching hospital, the Netherlands	9,000-30,000	-	13,554 children	MTS v.1	- Composite outcome "van Veen"; based on abnormal vital parameters, life-threatening conditions, diagnostics, interventions and follow-up
Veen, van, 2012[56]	1 academic children's and 1 teaching hospital, the Netherlands	9,000-15,000	-	11,260 children	MTS incl. modifications for children with fever	- Composite outcome "van Veen"; based on abnormal vital parameters, life-threatening conditions, diagnostics, interventions and follow-up
Waldrop, 1997[57]	Single teaching hospital, USA	65,000	5% (adults), 8% (children)	13,294 adults and 1528 children	Local triage system	- Hospital admission<br>- Mortality at the ED<br>- Length of stay at the ED<br>- Left without being seen<br>- Waiting time to examination
Worster, 2007[58]	2 academic hospitals, Canada	36,000-395,000	-	481 patients	CTAS and ESI	- Resource use, other<br>- Mortality: in-hospital mortality
Wuerz, 2000[59]	2 academic hospitals, USA	60,000 (average)	32%	493 adults	ESI	- Hospital admission<br>- Resource use based on ESI<br>- Length of stay at the ED<br>- Costs
Wuerz, 2001[60]	Single academic hospital, USA	50,000	-	180 adults	ESI	- Mortality: 6 month mortality
Wuerz, 2001[61]	2 academic hospitals, USA	50,000-60,000	28%	8,251 adults	ESI	- Hospital admission<br>- Length of stay at the ED

For peer review only

Wulp, van der 2009[62]	4 teaching hospitals, the Netherlands	30,000-47,000	21%	72,232 children and adults	ESI and MTS	- Hospital admission<br>- Mortality at the ED
Wulp, van der, 2011[63]	Single academic hospital, the Netherlands	21,000	-	544 adults	ESI	- Worthing Phsyiological Scoring System<br>- Pain score
Yuksen, 2016[64]	Single academic hospital, Thailand	-	-	520 patients	ESI and 4-level nurse triage	- Hospital admission<br>- Resource use based on ESI<br>- Length of stay at the ED<br>- Mortality at the ED<br>- Immediate lifesaving interventions
Zachariasse, 2016[65]	Single academic children's hospital, the Netherlands	7,000	-	50,062 children	MTS	- ICU admission
Zachariasse, 2017[66]	1 academic children's hospital, 1 teaching hospital and 1 community hospital, the Netherlands and Portugal	7,000 – 190,000	-	222,603 adults and 66,060 children	MTS	- ICU admission<br>- Composite outcome based on physiologic parameters, patient disposition and resource use

ATS, Australasian Triage Scale; *CTAS*, Canadian Triage and Acuity Scale; *ESI*, Emergency Severity Index; *MTS*, Manchester Triage System; *Ped*, Pediatric; *SATS,* South African Triage Scale; *TTS*, Taiwan Triage System;

For peer review only

References

1. Aeimchanbanjong K, Pandee U. Validation of different pediatric triage systems in the emergency department. *World J Emerg Med.* 2017;8:223-227.

2. Al-Hindi AA, Al-Akhfash AA, Fareed AM, Alhusainan KS, Algasomy SF, Althowainy IR. Efficacy of implementation of a 5 scale pediatric triage and acuity scale in pediatric emergency, Saudi Arabia. *Saudi Medical Journal.* 2014;35:999-1004.

3. Allon R, Feldman O, Karminsky A, Steinberg C, Leiba R, Shavit I. Validity of the Pediatric Canadian Triage Acuity Scale in a tertiary children's hospital in Israel. *Eur J Emerg Med.* 2017.

4. Baumann MR, Strout TD. Evaluation of the Emergency Severity Index (version 3) triage algorithm in pediatric patients. *Acad Emerg Med.* 2005;12:219-224.

5. Baumann MR, Strout TD. Triage of Geriatric Patients in the Emergency Department: Validity and Survival With the Emergency Severity Index. *Ann Emerg Med.* 2007;49:234-240.

6. Brillman JC, Doezema D, Tandberg D, et al. Triage: Limitations in predicting need for emergent care and hospital admission. *ANN EMERG MED.* 1996;27:493-500.

7. Bruijns SR, Wallis LA, Burch VC. A prospective evaluation of the Cape triage score in the emergency department of an urban public hospital in South Africa. *Emerg Med J.* 2008;25:398-402.

8. Chang YC, Ng CJ, Wu CT, Chen LC, Chen JC, Hsu KH. Effectiveness of a five-level Paediatric Triage System: An analysis of resource utilisation in the emergency department in Taiwan. *Emerg Med J.* 2012.

9. Chi CH, Huang CM. Comparison of the Emergency Severity Index (ESI) and the Taiwan triage system in predicting resource utilization. *J Formos Med Assoc.* 2006;105:617-625.

10. Dong SL, Bullard MJ, Meurer DP, et al. Predictive Validity of a Computerized Emergency Triage Tool. *Acad Emerg Med.* 2007;14:16-21.

11. Dugas AF, Kirsch TD, Toerper M, et al. An Electronic Emergency Triage System to Improve Patient Distribution by Critical Outcomes. *J Emerg Med.* 2016;50:910-918.

12. Eitel DR, Travers DA, Rosenau AM, Gilboy N, Wuerz RC. The Emergency Severity Index triage algorithm version 2 is reliable and valid. *Acad Emerg Med.* 2003;10:1070-1080.

13. Engan M, Hirth A, Tronnes H. Validation of a Modified Triage Scale in a Norwegian Pediatric Emergency Department. *Int J Pediatr.* 2018;2018:4676758.

14. Erimşah ME, Yaka E, Yilmaz S, Kama A, Pekdemir M. Inter-rater reliability and validity of the Ministry of Health of Turkey's mandatory emergency triage instrument. *EMA Emerg Med Australas.* 2015;27:210-215.

15. Fan MMW, Leung LP. Validation of the Hong Kong accident and Emergency Triage Guidelines. *Hong Kong Med J.* 2013;19:198-202.

16. Fong RY, Glen WSS, Mohamed Jamil AK, Tam WWS, Kowitlawakul Y. Comparison of the Emergency Severity Index versus the Patient Acuity Category Scale in an emergency setting. *Int Emerg Nurs.* 2018;41:13-18.

17. Funakoshi H, Shiga T, Homma Y, et al. Validation of the modified Japanese Triage and Acuity Scale-based triage system emphasizing the physiologic variables or mechanism of injuries. *Int J Emer Med.* 2016;9:1-6.

For peer review only

18. Ganapathy S, Yeo JG, Thia XHM, Hei GMA, Tham LP. The Singapore Paediatric Triage Scale Validation Study. *Singapore Med J.* 2018;59:205-209.

19. Van Gerven R, Delooz H, Sermeus W. Systematic triage in the emergency department using the Australian National Triage Scale: a pilot project. *Eur J Emerg Med.* 2001;8:3-7.

20. Ghafarypour-Jahrom M, Taghizadeh M, Heidari K, Derakhshanfar H. Validity and Reliability of the Emergency Severity Index and Australasian Triage System in Pediatric Emergency Care of Mofid Children's Hospital in Iran. *Bull Emerg Trauma.* 2018;6:329-333.

21. Gouin S, Gravel J, Amre DK, Bergeron S. Evaluation of the Paediatric Canadian Triage and Acuity Scale in a pediatric ED. *Am J Emerg Med.* 2005;23:243-247.

22. Gräff I, Goldschmidt B, Glien P, et al. The German version of the Manchester triage system and its quality criteria - First assessment of validity and reliability. *PLoS ONE.* 2014;9.

23. Gravel J, Manzano S, Arsenault M. Validity of the Canadian Paediatric Triage and Acuity Scale in a tertiary care hospital. *Can J Emerg Med.* 2009;11:23-28.

24. Gravel J, Gouin S, Goldman RD, et al. The Canadian Triage and Acuity Scale for children: A prospective multicenter evaluation. *Ann Emerg Med.* 2012;60:71-77.

25. Gravel J, Fitzpatrick E, Gouin S, et al. Performance of the Canadian Triage and Acuity Scale for children: a multicenter database study. *Ann Emerg Med.* 2013;61:27-32 e23.

26. Green NA, Durani Y, Brecher D, Depiero A, Loiselle J, Attia M. Emergency severity index version 4: A valid and reliable tool in pediatric emergency department triage. *Pediatr Emerg Care.* 2012;28:753-757.

27. Hansen LH, Mogensen CB, Wittenhoff L, Skjot-Arkil H. The danish regions pediatric triage model has a limited ability to detect both critically ill children as well as children to be sent home without treatment - a study of diagnostic accuracy. *Scand J Trauma Resusc Emerg Med.* 2017;25:55.

28. Hay E, Bekerman L, Rosenberg G, Peled R. Quality assurance of nurse triage: Consistency of results over three years. *Am J Emerg Med.* 2001;19:113-117.

29. van Ierland Y, van Veen M, Huibers L, Giesen P, Moll HA. Validity of telephone and physical triage in emergency care: The Netherlands Triage System. *Fam Pract.* 2011;28:334-341.

30. Jobe J, Ghuysen A, Gerard P, Hartstein G, D'Orio V. Reliability and validity of a new French-language triage algorithm: The ELISA scale. *Emerg Med J.* 2013.

31. Lee A, Hazlett CB, Chow S, et al. How to minimize inappropriate utilization of Accident and Emergency Departments: Improve the validity of classifying the general practice cases amongst the A&E attendees. *Health Policy.* 2003;66:159-168.

32. Lee JY, Oh SH, Peck EH, et al. The validity of the Canadian Triage and Acuity Scale in predicting resource utilization and the need for immediate life-saving interventions in elderly emergency department patients. *Scand J Trauma Resusc Emerg Med.* 2011;19:68.

33. Lin GX, Yang YL, Kudirka D, et al. Implementation of a Pediatric Emergency Triage System in Xiamen, China. *Chin Med J (Engl).* 2016;129:2416-2421.

34. Ma W, Gafni A, Goldman RD. Correlation of the Canadian Pediatric Emergency Triage and Acuity Scale to ED resource utilization. *Am J Emerg Med.* 2008;26:893-897.

For peer review only

35. Maleki M, Fallah R, Riahi L, Delavari S, Rezaei S. Effectiveness of Five-Level Emergency Severity Index Triage System Compared With Three-Level Spot Check: An Iranian Experience. *Arch Trauma Res.* 2015;4:e29214.

36. Maningas PA, Hime DA, Parker DE. The use of the soterion rapid triage system in children presenting to the Emergency Department. *J Emerg Med.* 2006;31:353-359.

37. Maningas PA, Hime DA, Parker DE, McMurry TA. The Soterion Rapid Triage System: Evaluation of inter-rater reliability and validity. *J Emerg Med.* 2006;30:461-469.

38. Martins HM, Cuna LM, Freitas P. Is Manchester (MTS) more than a triage system? A study of its association with mortality and admission to a large Portuguese hospital. *Emerg Med J.* 2009;26:183-186.

39. Meyer GD, Meyer TN, Gaunt CB. Validity of the South African Triage Scale in a rural district hospital. *Afr J Emerg Med.* 2018;8:145-149.

40. Ng CJ, Hsu KH, Kuan JT, et al. Comparison between Canadian Triage and Acuity Scale and Taiwan Triage System in emergency departments. *J Formos Med Assoc.* 2010;109:828-837.

41. Ng CJ, Yen ZS, Tsai JCH, et al. Validation of the Taiwan triage and acuity scale: A new computerised five-level triage system. *Emerg Med J.* 2011;28:1026-1031.

42. Platts-Mills TF, Travers D, Biese K, et al. Accuracy of the emergency severity index triage instrument for identifying elder emergency department patients receiving an immediate life-saving intervention. *Acad Emerg Med.* 2010;17:238-243.

43. Roukema J, Steyerberg EW, van Meurs A, Ruige M, van der Lei J, Moll HA. Validity of the Manchester Triage System in paediatric emergency care. *Emerg Med J.* 2006;23:906-910.

44. Santos AP, Freitas P, Martins HMG. Manchester triage system version II and resource utilisation in emergency department. *Emerg Med J.* 2013.

45. Seiger N, van Veen M, Almeida H, et al. Improving the Manchester Triage System for pediatric emergency care: an international multicenter study. *PLoS One.* 2014;9:e83267.

46. Steiner D, Renetseder F, Kutz A, et al. Performance of the Manchester Triage System in Adult Medical Emergency Patients: A Prospective Cohort Study. *J Emerg Med.* 2016;50:678-689.

47. Storm-Versloot MN, Ubbink DT, Kappelhof J, Luitse JSK. Comparison of an informally structured triage system, the emergency severity index, and the manchester triage system to distinguish patient priority in the emergency department. *Acad Emerg Med.* 2011;18:822-829.

48. Storm-Versloot MN, Vermeulen H, van Lammeren N, Luitse JS, Goslings JC. Influence of the Manchester triage system on waiting time, treatment time, length of stay and patient satisfaction; a before and after study. *Emerg Med J.* 2014;31:13-18.

49. Taboulet P, Moreira V, Haas L, et al. Triage with the French Emergency Nurses Classification in Hospital scale: Reliability and validity. *Eur J Emerg Med.* 2009;16:61-67.

50. Tanabe P, Gimbel R, Yarnold PR, Adams JG. The Emergency Severity Index (version 3) 5-level triage system scores predict ED resource consumption. *J Emerg Nurs.* 2004;30:22-29.

51. Tanabe P, Gimbel R, Yarnold PR, Kyriacou DN, Adams JG. Reliability and Validity of Scores on the Emergency Severity Index Version 3. *Acad Emerg Med.* 2004;11:59-65.

For peer review only

52. Travers DA, Waller AE, Bowling JM, Flowers D, Tintinalli J. Five-level triage system more effective than three-level in tertiary emergency department. *J Emerg Nurs.* 2002;28:395-400.

53. Travers DA, Waller AE, Katznelson J, Agans R. Reliability and validity of the emergency severity index for pediatric triage. *Acad Emerg Med.* 2009;16:843-849.

54. Twomey M, Cheema B, Buys H, et al. Vital signs for children at triage: A multicentre validation of the revised South African Triage Scale (SATS) for children. *Samj South African Medical Journal.* 2013;103:304-308.

55. van Veen M, Steyerberg EW, Ruige M, et al. Manchester triage system in paediatric emergency care: prospective observational study. *BMJ.* 2008;337:a1501.

56. Van Veen M, Steyerberg EW, Van't Klooster M, et al. The Manchester triage system: Improvements for paediatric emergency care. *Emerg Med J.* 2012;29:654-659.

57. Waldrop RD, Harper DE, Mandry C. Prospective assessment of triage in an urban emergency department. *SOUTH MED J.* 1997;90:1208-1212.

58. Worster A, Fernandes CM, Eva K, Upadhye S. Predictive validity comparison of two five-level triage acuity scales. *Eur J Emerg Med.* 2007;14:188-192.

59. Wuerz RC, Milne LW, Eitel DR, Travers D, Gilboy N. Reliability and validity of a new five-level triage instrument. *Acad Emerg Med.* 2000;7:236-242.

60. Wuerz R, Eitel D, Gilboy N, et al. Emergency severity index triage category is associated with six-month survival. *Acad Emerg Med.* 2001;8:61-64.

61. Wuerz RC, Travers D, Gilboy N, Eitel DR, Rosenau A, Yazhari R. Implementation and refinement of the Emergency severity index. *Acad Emerg Med.* 2001;8:170-176.

62. van der Wulp I, Schrijvers AJP, van Stel HF. Predicting admission and mortality with the Emergency Severity Index and the Manchester Triage System: a retrospective observational study. *Emergency Medicine Journal.* 2009;26:506-509.

63. Van Der Wulp I, Rullmann HAA, Leenen LPH, Van Stel HF. Associations of the Emergency Severity Index triage categories with patients' vital signs at triage: A prospective observational study. *Emerg Med J.* 2011;28:1032-1035.

64. Yuksen C, Sawatmongkornkul S, Suttabuth S, Sawanyawisuth K, Sittichanbuncha Y. Emergency severity index compared with 4-level triage at the emergency department of Ramathibodi University Hospital. *Asian Biomed.* 2016;10:155-161.

65. Zachariasse JM, Kuiper JW, de Hoog M, Moll HA, van Veen M. Safety of the Manchester Triage System to Detect Critically Ill Children at the Emergency Department. *J Pediatr.* 2016;177:232-237.e231.

66. Zachariasse JM, Seiger N, Rood PPM, et al. Validity of the Manchester triage system in emergency care: A prospective observational study. *PLoS ONE.* 2017;12.

Appendix 3. Risk of bias assessment

Table 3.1 Risk of bias and applicability concerns summary: review authors' judgements about each domain for each included study

For peer review only

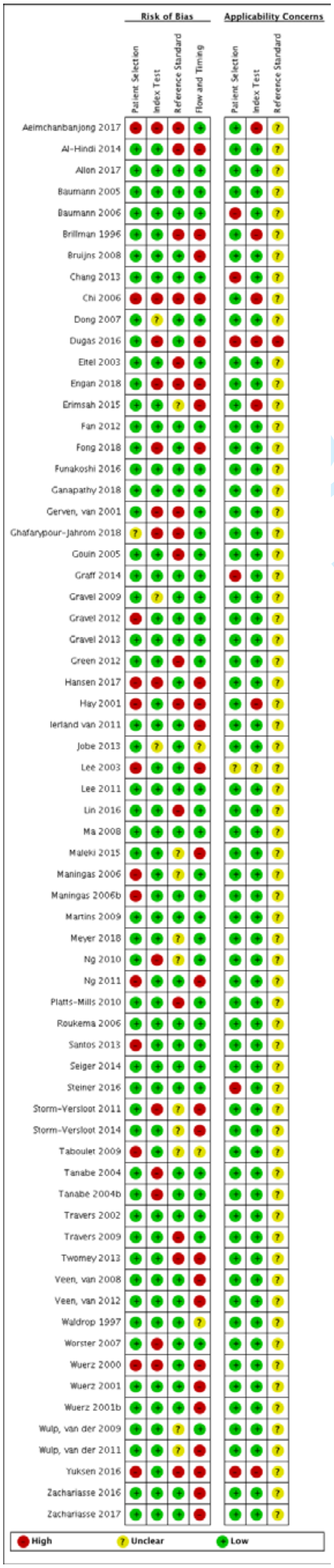

For peer review only

Appendix 4. Reference standards used in studies evaluating triage systems in the emergency department

**Patient disposition and follow-up**	Hospital admission	47 studies
	ICU admission	13 studies
	Follow-up at outpatient clinic or GP	1 study
**Waiting times**	Length of stay at the ED	27 studies
	Length of stay in hospital	5 studies
	Waiting time to physician	4 studies
	Other: Waiting time to nurse, Waiting time to examination, Waiting time to first lab results, Waiting time from arrival to treatment room, Waiting time from treatment room to discharge from ED, Waiting time for care	1 study each
**Resource use**	Resource use based on ESI criteria	14 studies
	Costs	8 studies
	Current Procedural Terminology (CPT) codes	2 studies
	Other	15 studies
**Mortality**	Mortality at the ED	12 studies
	In-hospital mortality	5 studies
	1 year mortality	2 studies
	Other: 30-day mortality, 60-day mortality, 6 month mortality	1 study each
**Composite outcomes**	"Immediate lifesaving interventions"	4 studies
	"MTS reference standard": Combination of abnormal physiologic parameters, life-threatening conditions, resource use, follow-up	4 studies
	"Critical or time-sensitive outcomes": in-hospital mortality, intensive care unit admission or transfer to operating room or catheterization suite	1 study
	"Actual clinical outcomes": Combination of life threatening condition, transfer to higher hospital, ICU, or hospital admission	1 study
	Composite outcome based on physiologic parameters, patient disposition and resource use	1 study
**Physiologic parameters**	Worthing Phsyiological Scoring System	1 study
	Pain score	1 study
**Other**	Expert opinion	9 studies
	Left without being seen	5 studies
	Patient or parent satisfaction	2 studies
	Level of prehospital care	1 study
	Sentinel diagnosis	1 study
	Pediatric risk of admission (PRISA) score	1 study

For peer review only

Appendix 5. Reference standard per urgency category

Figure 5.1 Proportion of ICU admissions per urgency category in the most commonly evaluated triage systems.

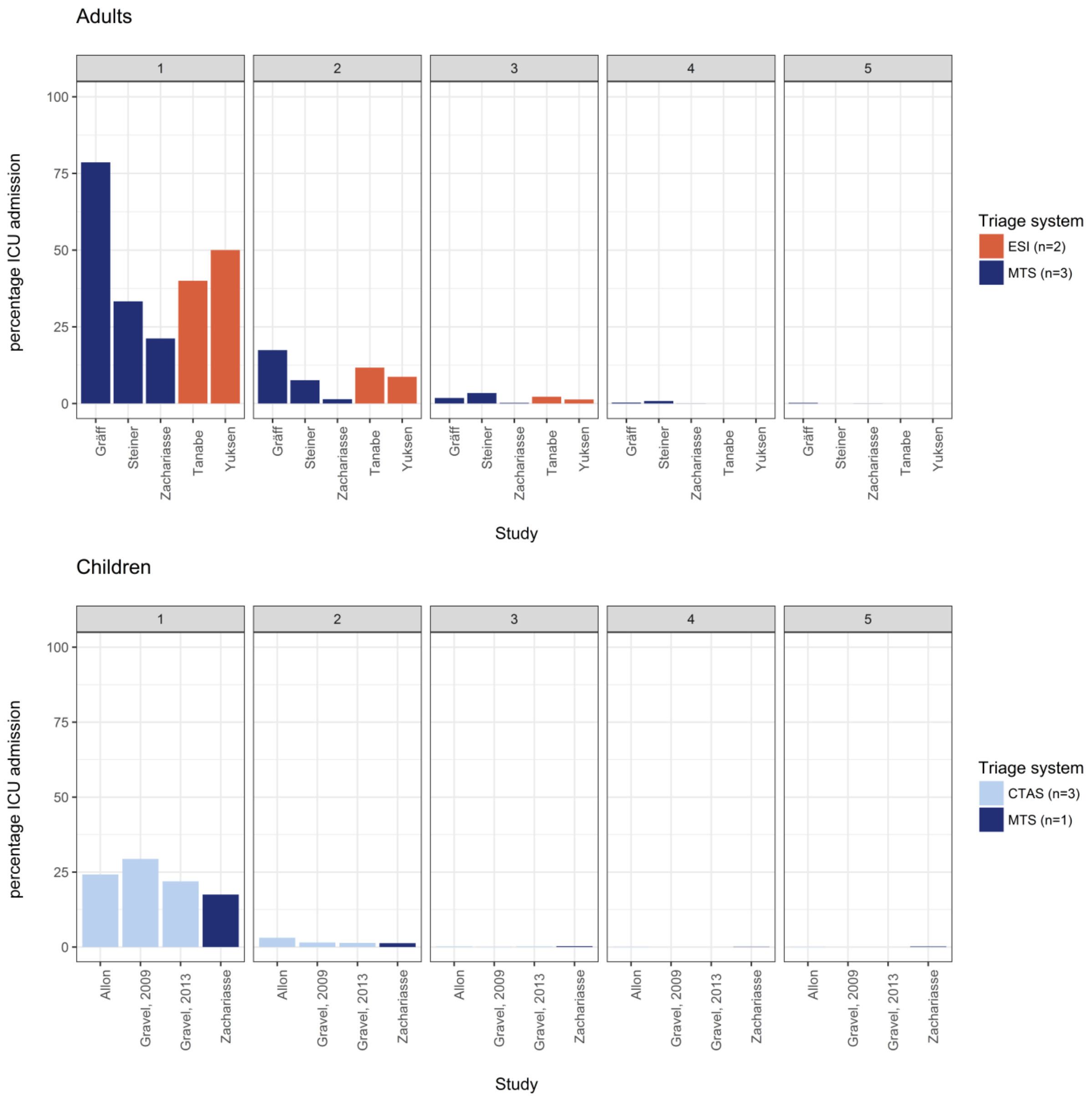

Figure 5.2 Proportion of patients discharged home per urgency category in the most commonly evaluated triage systems

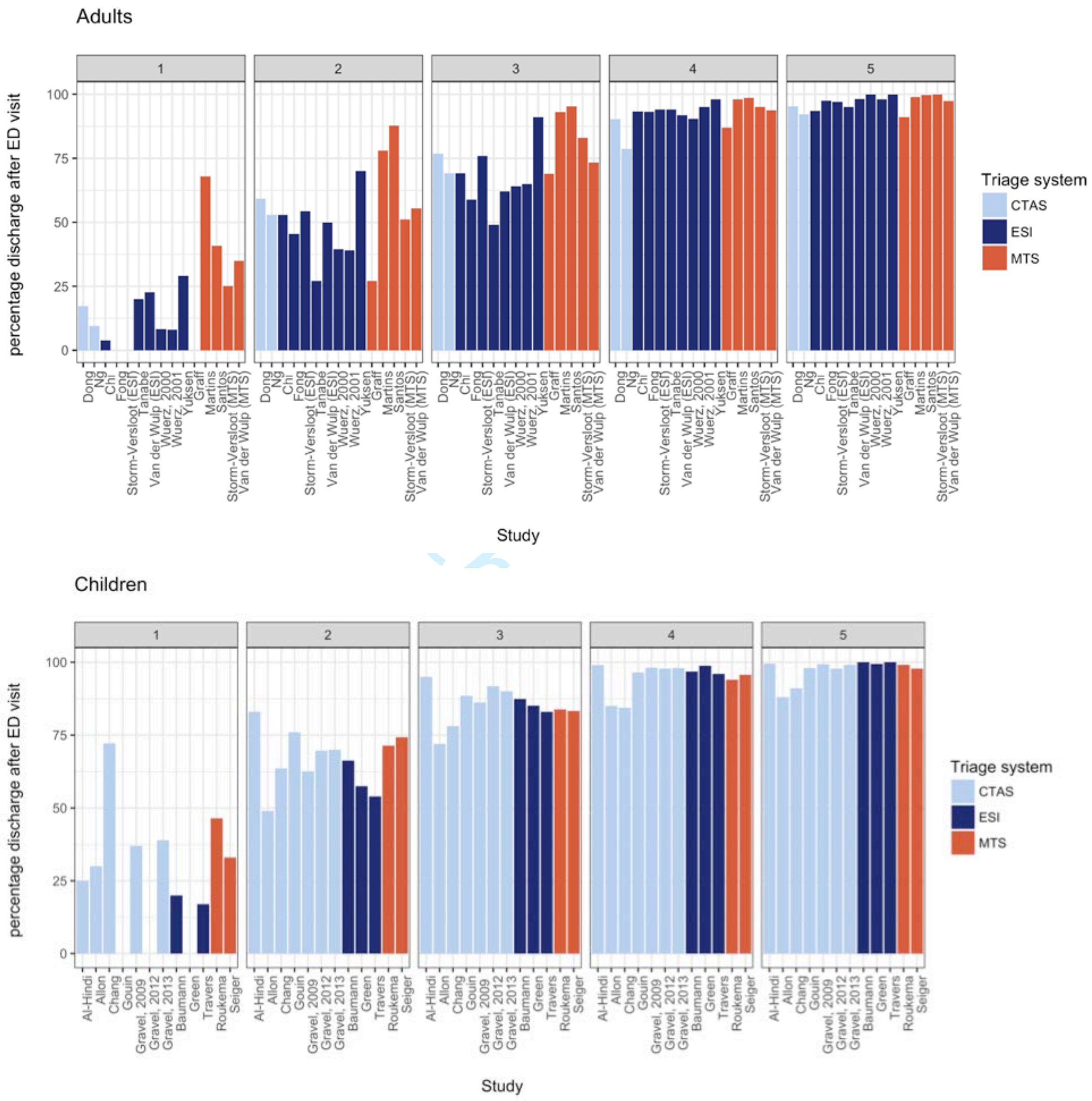

Appendix 6. Other reference standards

Other reference standards

14 studies used resource use according to ESI criteria as a reference standard of which 7 provided data to construct a 2x2 table and were conducted in one of the commonly used triage systems. One study evaluated resource use in the MTS, and all others studied the ESI.

Because the ESI states that resource prediction should only be used for the less acute patients, we used a different cut-off for dichotomization to calculate sensitivity and specificity. We dichotomized the triage categories into a high urgent group consisting of triage urgency levels 1, 2 and 3 with a low urgent group consisting of triage categories 4 and 5.

Overall, sensitivity of the triage systems to accurately classify patients with 2 or more resources in the highest urgency categories ranged from 0.75 (95%CI 0.68 to 0.81) to 0.91 (95%CI 0.87 to 0.94) in adults and from 0.76 (95%CI 0.70 to 0.82) to 0.90 (95%CI 0.87 to 0.92) in children (Fig. 7). Specificity of the triage system to accurately classify patients with 1 or 0 resources in the lowest two urgency categories ranged from 0.73 (95%CI 0.66 to 0.79) to 0.81 (95%CI 0.73 to 0.88) in adults and from 0.42 (95%CI 0.39 to 0.46) to 0.75 (95%CI 0.69 to 0.81) in children.

For peer review only

Fig 6.1. Forest plot evaluating sensitivity and specificity of triage systems using resource use according to ESI criteria as reference standard

ESI

Study	TP	FP	FN	TN	Age group	Sensitivity (95% CI)	Specificity (95% CI)
Baumann 2005	101	183	11	215	Children	0.90 [0.83, 0.95]	0.54 [0.49, 0.59]
Travers 2009	480	200	56	438	Children	0.90 [0.87, 0.92]	0.69 [0.65, 0.72]
Green 2012	158	206	49	367	Children	0.76 [0.70, 0.82]	0.64 [0.60, 0.68]
Wuerz 2000	241	59	36	157	Adults / Unspecified	0.87 [0.82, 0.91]	0.73 [0.66, 0.79]
Yuksen 2016	278	53	29	160	Adults / Unspecified	0.91 [0.87, 0.94]	0.75 [0.69, 0.81]
Fong 2018	134	23	45	98	Adults / Unspecified	0.75 [0.68, 0.81]	0.81 [0.73, 0.88]

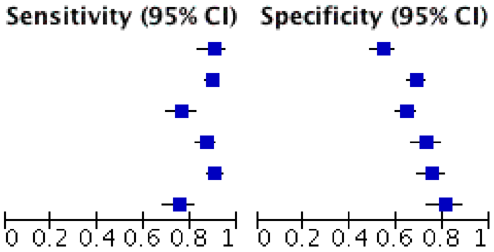

MTS

Study	TP	FP	FN	TN	Age group	Sensitivity (95% CI)	Specificity (95% CI)
Roukema 2006	205	469	51	340	Children	0.80 [0.75, 0.85]	0.42 [0.39, 0.46]

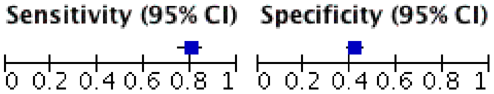

ESI = Emergency Severity Index; TP = true positive; FP = false positive; FN = false negative; TN = true negative; MTS = Manchester Triage System

view only

Appendix 7. Determinants of triage systems' performance

Figure 7.1. Forest plot evaluating sensitivity and specificity of triage systems for identifying low urgency patients as defined by discharge home after the ED visit, ordered by annual census as a marker of patient volume

Adults

Study	TP	FP	FN	TN	Annual Census	Sensitivity (95% CI)	Specificity (95% CI)
Graff 2014	6497	2132	8463	28377	23000.0	0.43 [0.43, 0.44]	0.93 [0.93, 0.93]
Storm-Versloot 2011	66	76	96	638	31000.0	0.41 [0.33, 0.49]	0.89 [0.87, 0.92]
Storm-Versloot 2011	73	72	76	651	31000.0	0.49 [0.41, 0.57]	0.90 [0.88, 0.92]
Yuksen 2016	38	68	22	392	33936.0	0.63 [0.50, 0.75]	0.85 [0.82, 0.88]
Van der Wulp 2009	2259	2146	5911	27658	38500.0	0.28 [0.27, 0.29]	0.93 [0.93, 0.93]
Van der Wulp 2009	2641	3183	4415	24019	38500.0	0.37 [0.36, 0.39]	0.88 [0.88, 0.89]
Wuerz 2001	1113	674	1260	5204	55000.0	0.47 [0.45, 0.49]	0.89 [0.88, 0.89]
Chi 2006	713	747	376	1336	58000.0	0.65 [0.63, 0.68]	0.64 [0.62, 0.66]
Wuerz 2000	91	53	68	281	60000.0	0.57 [0.49, 0.65]	0.84 [0.80, 0.88]
Dong 2007	1519	1947	4189	21869	67000.0	0.27 [0.25, 0.28]	0.92 [0.91, 0.92]
Tanabe 2004	129	48	75	151	70000.0	0.63 [0.56, 0.70]	0.76 [0.69, 0.82]
Santos 2013	485	3080	535	19515	100000.0	0.48 [0.44, 0.51]	0.86 [0.86, 0.87]
Martins 2009	18265	63803	12464	222090	128000.0	0.59 [0.59, 0.60]	0.78 [0.78, 0.78]

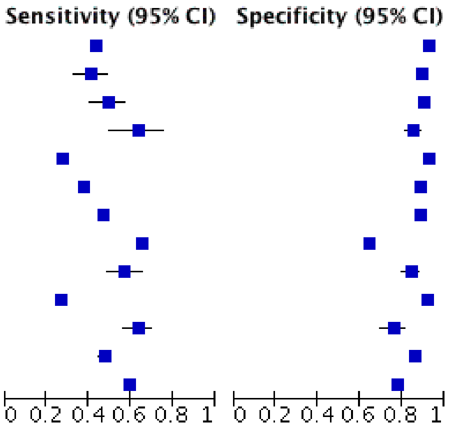

Children

Study	TP	FP	FN	TN	Annual Census	Sensitivity (95% CI)	Specificity (95% CI)
Aeimchanbanjong 2017	62	239	62	679	10000.0	0.50 [0.41, 0.59]	0.74 [0.71, 0.77]
Aeimchanbanjong 2017	64	165	59	753	10000.0	0.52 [0.43, 0.61]	0.82 [0.79, 0.84]
Aeimchanbanjong 2017	70	285	53	633	10000.0	0.57 [0.48, 0.66]	0.69 [0.66, 0.72]
Allon 2017	2631	2330	17726	60922	16722.0	0.13 [0.12, 0.13]	0.96 [0.96, 0.96]
Roukema 2006	147	256	62	600	30000.0	0.70 [0.64, 0.76]	0.70 [0.67, 0.73]
Chang 2013	3104	6821	6276	26327	42839.0	0.33 [0.32, 0.34]	0.79 [0.79, 0.80]
Green 2012	33	42	47	658	45000.0	0.41 [0.30, 0.53]	0.94 [0.92, 0.96]
Baumann 2005	44	66	28	372	53000.0	0.61 [0.49, 0.72]	0.85 [0.81, 0.88]
Gravel 2009	1937	2746	3124	50722	60000.0	0.38 [0.37, 0.40]	0.95 [0.95, 0.95]
Gouin 2005	8	19	38	472	65000.0	0.17 [0.08, 0.31]	0.96 [0.94, 0.98]
Al-Hindi 2014	65	295	45	2609	155000.0	0.59 [0.49, 0.68]	0.90 [0.89, 0.91]

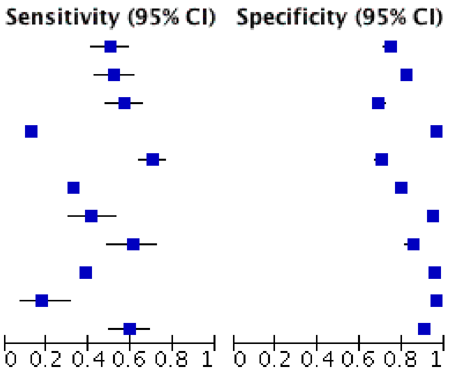

TP = true positive; FP = false positive; FN = false negative; TN = true negative

For peer review only

Figure 7.2. Forest plot evaluating sensitivity and specificity of triage systems for identifying low urgency patients as defined by discharge home after the ED visit, ordered by percentage hospitalization as a marker of case-mix severity of illness

Adults

Study	TP	FP	FN	TN	Hospitalization rate	Sensitivity (95% CI)	Specificity (95% CI)
Santos 2013	485	3080	535	19515	5.0	0.48 [0.44, 0.51]	0.86 [0.86, 0.87]
Martins 2009	18265	63803	12464	222090	10.0	0.59 [0.59, 0.60]	0.78 [0.78, 0.78]
Yuksen 2016	38	68	22	392	12.0	0.63 [0.50, 0.75]	0.85 [0.82, 0.88]
Storm-Versloot 2011	66	76	96	638	18.0	0.41 [0.33, 0.49]	0.89 [0.87, 0.92]
Storm-Versloot 2011	73	72	76	651	18.0	0.49 [0.41, 0.57]	0.90 [0.88, 0.92]
Dong 2007	1519	1947	4189	21869	19.0	0.27 [0.25, 0.28]	0.92 [0.91, 0.92]
Van der Wulp 2009	2259	2146	5911	27658	21.0	0.28 [0.27, 0.29]	0.93 [0.93, 0.93]
Van der Wulp 2009	2641	3183	4415	24019	21.0	0.37 [0.36, 0.39]	0.88 [0.88, 0.89]
Fong 2018	6	5	68	221	25.0	0.08 [0.03, 0.17]	0.98 [0.95, 0.99]
Wuerz 2001	1113	674	1260	5204	28.0	0.47 [0.45, 0.49]	0.89 [0.88, 0.89]
Wuerz 2000	91	53	68	281	32.0	0.57 [0.49, 0.65]	0.84 [0.80, 0.88]
Graff 2014	6497	2132	8463	28377	33.0	0.43 [0.43, 0.44]	0.93 [0.93, 0.93]
Chi 2006	713	747	376	1336	34.0	0.65 [0.63, 0.68]	0.64 [0.62, 0.66]

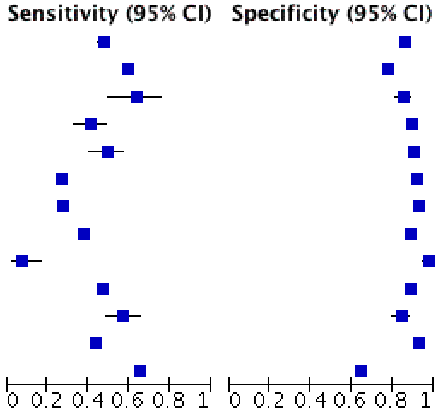

Children

Study	TP	FP	FN	TN	Hospitalization rate	Sensitivity (95% CI)	Specificity (95% CI)
Al-Hindi 2014	65	295	45	2609	4.0	0.59 [0.49, 0.68]	0.90 [0.89, 0.91]
Gouin 2005	8	19	38	472	9.0	0.17 [0.08, 0.31]	0.96 [0.94, 0.98]
Gravel 2009	1937	2746	3124	50722	9.0	0.38 [0.37, 0.40]	0.95 [0.95, 0.95]
Green 2012	33	42	47	658	10.0	0.41 [0.30, 0.53]	0.94 [0.92, 0.96]
Aeimchanbanjong 2017	62	239	62	679	12.0	0.50 [0.41, 0.59]	0.74 [0.71, 0.77]
Aeimchanbanjong 2017	64	165	59	753	12.0	0.52 [0.43, 0.61]	0.82 [0.79, 0.84]
Aeimchanbanjong 2017	70	285	53	633	12.0	0.57 [0.48, 0.66]	0.69 [0.66, 0.72]
Baumann 2005	44	66	28	372	14.0	0.61 [0.49, 0.72]	0.85 [0.81, 0.88]
Chang 2013	3104	6821	6276	26327	23.0	0.33 [0.32, 0.34]	0.79 [0.79, 0.80]
Allon 2017	2631	2330	17726	60922	24.0	0.13 [0.12, 0.13]	0.96 [0.96, 0.96]

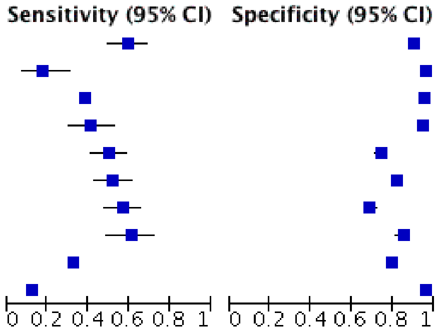

TP = true positive; FP = false positive; FN = false negative; TN = true negative

MOOSE Checklist for Meta-analyses of Observational Studies

Item No	Recommendation	Reported on Page No
Reporting of background should include		
1	Problem definition	p.6
2	Hypothesis statement	-
3	Description of study outcome(s)	p.6, 7, 8
4	Type of exposure or intervention used	p.6,7
5	Type of study designs used	p.7
6	Study population	p.6,7
Reporting of search strategy should include		
7	Qualifications of searchers (eg, librarians and investigators)	Title page
8	Search strategy, including time period included in the synthesis and key words	p.7, appendix 1
9	Effort to include all available studies, including contact with authors	p.7
10	Databases and registries searched	p.7, appendix 1
11	Search software used, name and version, including special features used (eg, explosion)	-
12	Use of hand searching (eg, reference lists of obtained articles)	Fig. 1
13	List of citations located and those excluded, including justification	Fig. 1, appendix 2
14	Method of addressing articles published in languages other than English	p.7
15	Method of handling abstracts and unpublished studies	p.7
16	Description of any contact with authors	-
Reporting of methods should include		
17	Description of relevance or appropriateness of studies assembled for assessing the hypothesis to be tested	p.7
18	Rationale for the selection and coding of data (eg, sound clinical principles or convenience)	p.7
19	Documentation of how data were classified and coded (eg, multiple raters, blinding and interrater reliability)	p.7
20	Assessment of confounding (eg, comparability of cases and controls in studies where appropriate)	-
21	Assessment of study quality, including blinding of quality assessors, stratification or regression on possible predictors of study results	p.8
22	Assessment of heterogeneity	p.9
23	Description of statistical methods (eg, complete description of fixed or random effects models, justification of whether the chosen models account for predictors of study results, dose-response models, or cumulative meta-analysis) in sufficient detail to be replicated	p.9
24	Provision of appropriate tables and graphics	Tables 1-2, Figs 1-9
Reporting of results should include		
25	Graphic summarizing individual study estimates and overall estimate	Figs 3,5,7
26	Table giving descriptive information for each study included	Appendix 2
27	Results of sensitivity testing (eg, subgroup analysis)	Figs 8-9
28	Indication of statistical uncertainty of findings	-

For peer review only - http://bmjopen.bmj.com/site/about/guidelines.xhtml

Item No	Recommendation	Reported on Page No
Reporting of discussion should include		
29	Quantitative assessment of bias (eg, publication bias)	p.29-30
30	Justification for exclusion (eg, exclusion of non-English language citations)	-
31	Assessment of quality of included studies	p.29-30
Reporting of conclusions should include		
32	Consideration of alternative explanations for observed results	p.30-31
33	Generalization of the conclusions (ie, appropriate for the data presented and within the domain of the literature review)	p.30-31
34	Guidelines for future research	p.31
35	Disclosure of funding source	p.3

From: Stroup DF, Berlin JA, Morton SC, et al, for the Meta-analysis Of Observational Studies in Epidemiology (MOOSE) Group. Meta-analysis of Observational Studies in Epidemiology. A Proposal for Reporting. *JAMA*. 2000;283(15):2008-2012. doi: 10.1001/jama.283.15.2008.

For peer review only

THE PERFORMANCE OF TRIAGE SYSTEMS IN EMERGENCY CARE: A SYSTEMATIC REVIEW AND META-ANALYSIS

Journal:	*BMJ Open*
Manuscript ID	bmjopen-2018-026471.R2
Article Type:	Research
Date Submitted by the Author:	23-Mar-2019

Complete List of Authors:	Zachariasse, Joany; Erasmus MC-Sophia Childrens Hospital, Department of Pediatrics<br>van der Hagen, Vera; Erasmus MC-Sophia Childrens Hospital, Department of Pediatrics<br>Seiger, Nienke; Erasmus MC-Sophia Childrens Hospital, Department of Pediatrics<br>Mackway-Jones, Kevin; Manchester Royal Infirmary, Emergency Department<br>van Veen, Mirjam; Groene Hart Ziekenhuis, Pediatrics; Erasmus MC-Sophia Childrens Hospital, Department of Pediatrics<br>Moll, Henriette; Erasmus MC-Sophia Childrens Hospital, Department of Pediatrics

**Primary Subject Heading**:	Emergency medicine
Secondary Subject Heading:	Epidemiology
Keywords:	ACCIDENT & EMERGENCY MEDICINE, EPIDEMIOLOGY, Paediatric A&E and ambulatory care < PAEDIATRICS, PUBLIC HEALTH

THE PERFORMANCE OF TRIAGE SYSTEMS IN EMERGENCY CARE: A SYSTEMATIC REVIEW AND META-ANALYSIS

Joany M Zachariasse*, Vera van der Hagen*, Nienke Seiger, Kevin C Mackway-Jones, Mirjam van Veen, Henriëtte A Moll

* Both authors contributed equally

Department of Paediatrics, Erasmus MC- Sophia Children's Hospital, University Medical Centre Rotterdam, PO Box 2060, 3000 CB Rotterdam, The Netherlands

Joany M Zachariasse

PhD-student

Department of Paediatrics, Erasmus MC- Sophia Children's Hospital, University Medical Centre Rotterdam, PO Box 2060, 3000 CB Rotterdam, The Netherlands

Vera van der Hagen

MD

Department of Paediatrics, Erasmus MC- Sophia Children's Hospital, University Medical Centre Rotterdam, PO Box 2060, 3000 CB Rotterdam, The Netherlands

Nienke Seiger

Paediatric resident

Emergency Department, Manchester Royal Infirmary, Oxford Road, Manchester M13 9WL, UK

Kevin C Mackway Jones

Professor of Emergency Medicine

Department of Paediatrics, Erasmus MC- Sophia Children's Hospital, University Medical Centre Rotterdam, PO Box 2060, 3000 CB Rotterdam, The Netherlands

Currently: Department of Paediatrics, Groene Hart Ziekenhuis, Postal Code 1098, 2800 BB Gouda, The Netherlands

Mirjam van Veen

Paediatrician

For peer review only

For peer review only - http://bmjopen.bmj.com/site/about/guidelines.xhtml

Department of Paediatrics, Erasmus MC- Sophia Children's Hospital, University Medical Centre Rotterdam, PO Box 2060, 3000 CB Rotterdam, The Netherlands

Henriëtte A Moll

Professor of Paediatrics

Correspondence to:
Henriëtte A Moll
h.a.moll@erasmusmc.nl

Word count: 3999

Word count abstract: 275

Abbreviations:

ATS Australasian Triage Scale

CTAS Canadian Triage and Acuity Scale

ED emergency department

ESI Emergency Severity Index

FN false negative

FP false positive

MTS Manchester Triage System

TN true negative

TP true positive

Funding: This research received no specific grant from any funding agency in the public, commercial or not-for-profit sectors

Competing interests: No support from any organisation for the submitted work; KMJ is chair of the Manchester Triage Group; there are no other relationships or activities that could appear to have influenced the submitted work.

Dating sharing: No additional data available

For peer review only

For peer review only - http://bmjopen.bmj.com/site/about/guidelines.xhtml

Abstract

Objective

To assess and compare the performance of triage systems for identifying high and low urgency patients in the emergency department

Design

Systematic review and meta-analysis

Data sources

EMBASE, Medline OvidSP, Cochrane central, Web-of-science and CINAHL databases from 1980 to 2016 with the final update in December 2018.

Eligibility criteria

Studies that evaluated an emergency medical triage system, assessed validity using any reference standard as proxy for true patient urgency and were written in English. Studies conducted in low(er)-income countries, based on case-scenarios or involving less than 100 patients were excluded.

Review methods

Reviewers identified studies, extracted data, and assessed the quality of the evidence independently and in duplicate. The QUADAS-2 checklist was used to assess risk of bias. Raw data were extracted to create 2x2 tables and calculate sensitivity and specificity. ED patient volume and case-mix severity of illness were investigated as determinants of triage systems' performance.

Results

Sixty-six eligible studies evaluated thirty-three different triage systems. Comparisons were restricted to the three triage systems that had at least multiple evaluations using the same reference standard (Canadian Triage and Acuity Scale, Emergency Severity Index, and Manchester Triage System). Overall validity to identify high and low urgency patients of each triage system was moderate to good, but performance was highly variable. In a subgroup analysis, no clear association was found between ED patient volume or case-mix severity of illness and triage systems' performance.

Conclusions

Established triage systems show a reasonable validity for the triage of patients at the ED, but performance varies considerably. Important research questions that remain are what determinants influence triage systems' performance and how the performance of existing triage systems can be improved.

For peer review only - http://bmjopen.bmj.com/site/about/guidelines.xhtml

Strengths and limitations of the study

- The present study was based on a comprehensive and systematic search and attempted to include as many relevant studies as possible

- This study is the first synthesis of the available evidence on triage systems as previous reviews have merely described the results of the individual studies

- The majority of triage systems were evaluated by less than three studies. Even for the three most commonly used triage systems, few evaluations were available due to the variety of reference standards used

- Due to the limited number of studies and the heterogeneity of study populations it was difficult to compare results across studies and we could not provide summary estimates

For peer review only

For peer review only - http://bmjopen.bmj.com/site/about/guidelines.xhtml

Introduction

Overcrowding of emergency departments (EDs) is a universal and ever increasing problem.[1-3] Therefore, most EDs have a triage system in place to facilitate the prioritisation of patients. In recent years several formal triage scales have been developed to standardize the approach to triage. These include amongst others the Australasian Triage Scale, the Canadian Triage and Acuity Scale (CTAS), the Emergency Severity Index (ESI) and the Manchester Triage System (MTS).[4-7]

It is important to evaluate the performance of triage systems for their ability to accurately distinguish between both high and low urgency patients. The correct classification of high urgency patients is related to patient safety, because misclassification of high urgency patients to a low urgency level causes delay in diagnosis and treatment, potentially leading to morbidity or mortality. The correct classification of low urgency patients increases efficiency of the ED flow and reduces waiting times for the truly high urgency ED visits.

Research regarding the performance of triage systems mainly consists of observational studies in heterogeneous populations using a variety of reference standards. Previous reviews have primarily described the results of these individual studies without combining and interpreting the evidence into overall conclusions.[8-10] A systematic appraisal of the performance of commonly used triage systems can inform clinicians and policy makers about the safety and efficacy of available triage systems and provide insights on which triage system is safe and efficient to use. Moreover it can highlight gaps in current research and propose directions for future studies.

The aim of this systematic review is to provide a comprehensive overview of current evidence on the performance of triage systems. We assessed and compared the performance of the most commonly used triage systems for the prioritisation of high- and low urgency patients at the ED, compared to any reference standard that is a proxy of true patient urgency. Furthermore, we aimed to investigate whether patient volume and case-mix at the ED, are determinants of triage systems' performance.

For peer review only

For peer review only - http://bmjopen.bmj.com/site/about/guidelines.xhtml

Methods

Search strategy

Meta-analysis of Observational Studies in Epidemiology (MOOSE) guidelines were followed for the conduct of this study.[11] We conducted a systematic review using a broad search strategy to identify all studies assessing the performance of triage systems in emergency care when compared to any reference standard that is a proxy of true patient urgency. A search strategy was developed by a health sciences librarian and included medical subject headings (MeSH) and text words related to triage, emergency care and validity (Appendix 1). We searched EMBASE, Medline OvidSP, Cochrane central, Web-of-science and CINAHL databases from 1980 to 2016 with the final update in December 2018.

Study eligibility

Studies were selected that assess the performance of triage systems in emergency care with a defined outcome measure as a proxy of true patient-urgency. We selected studies based on the following PICO:

Population: We included studies evaluating triage in the unselected group of patients attending the emergency department. We excluded studies restricted to specific patient subgroups (such as patients with specific diseases).

Interventions: We included any studies assessing emergency department triage systems, defined as any tool aimed to classify patients at the emergency department based on the urgency or severity of their condition. We did not include studies evaluating trauma triage systems or early warning scores.

Comparators: Since no golden standard for the evaluation of triage systems exists, we included all studies evaluating the performance of triage systems using one or more defined reference standard as a proxy for true patient urgency.

Outcome: We defined outcome as the sensitivity and specificity of the triage system for the identification of high-urgent and low-urgent patients. A-priori, we selected mortality at the ED and ICU admission after the ED visit as reference standard for high-urgency and discharge home after the ED visit as reference standard for low urgency. We additionally considered any other reference standard with sufficient evaluations.

For peer review only - http://bmjopen.bmj.com/site/about/guidelines.xhtml

Letters, abstracts, reviews, conference proceedings, and case reports were excluded as well as studies not written in English. We excluded studies with less than 100 patients and studies based on case scenarios because these studies have of a high risk of bias. Moreover, we excluded studies conducted in low or lower-income economies.[12] The unique characteristics of EDs in these countries, including the number of patients, epidemiology of diseases and available resources, make study results difficult to compare to middle or higher income countries. Two reviewers (MV and NS or JZ and VH) independently assessed eligibility for inclusion. Disagreements in article selection were resolved through discussion.

Data extraction

Two reviewers (JZ and VH) independently extracted data from each of the included studies. A single study could consist of multiple "triage evaluations", defined as the analysis of a single triage system in a single age group. Predefined age groups were 1) children; 2) adults or a combination of age groups; and 3) elderly. For each of the triage evaluations, the reviewers extracted the total number of included patients, the number of patients in each of the urgency categories of the triage system, the type of reference standard used, and the number of patients with a positive reference standard in each urgency category. If studies were based on overlapping data, we used the results from the most recent publication. For descriptive purposes we also collected data on study design and methods, patient demographics, and characteristics of the settings in which the study was performed.

Quality assessment

Two reviewers independently assessed quality of the selected articles using the QUADAS-2 (Quality Assessment of studies of Diagnostic Accuracy included in Systematic Reviews) checklist.[13] The QUADAS-2 evaluates 4 domains: patient selection, index test, reference standard, and flow and timing. Each domain is assessed in terms of risk of bias, and the first 3 domains also in terms of applicability. Because triage systems have some specific features as compared to other diagnostic tests, we adjusted the "reference standard domain" to make it applicable to our research question. We did not appraise whether the reference standard was interpreted without knowledge of the result of the index test, because this is unlikely when triage is applied in routine care. We did evaluate, however, whether data on the outcome was collected blinded to the result of the index test. Moreover, we did not judge the

For peer review only - http://bmjopen.bmj.com/site/about/guidelines.xhtml

applicability of the reference standard because there is no consensus on this topic.[14] Therefore, we included all studies with reference standard that were a proxy for patient urgency. Any disagreements between reviewers were resolved in a consensus meeting.

Data analysis

We used descriptive analyses to provide an overview of the available evidence on triage systems. Further analyses were restricted to triage systems that underwent at least 3 evaluations with the same reference standard. The primary outcome of our review was sensitivity and specificity of each of the triage systems for the identification of high and low urgency patients. Because there is no golden standard to determine "true" patient urgency, we a-priori selected 3 reference standards as proxy for patient urgency. We considered mortality at the ED and ICU admission after the ED visits as reference standard for high urgency, and discharge after the ED visit (i.e. patients not admitted to hospital) as reference standard for low urgency. Although these measures are not perfect, they approximate the desired outcome: most patients that die at the ED or require ICU admission are of high urgency, while most patients that are discharged after the ED visit are not. Moreover, these measures are suitable for the analyses of large datasets and commonly reported in research on triage systems.[14] In addition, we considered any other reference standard with sufficient evaluations in the same triage system.

We calculated two by two tables of triage system against the reference standard for each individual study. Because triage systems are ordinal scales, we dichotomized the urgency categories into a high urgency and low urgency group. High urgency was defined as triage urgency level 1 (3-level systems) or triage urgency level 1 and 2 (4-level and 5-level systems).

We calculated sensitivities and specificities, and presented the results as forest plots. We aimed to summarize the diagnostic accuracy data using a bivariate random effects model, but due to the substantial heterogeneity between studies this was not possible. For clinical practice and for benchmarking purposes, we calculated the proportion of patients with a positive reference standard per urgency category. These results are displayed in a bar chart to enable comparison between studies and between triage systems.

We hypothesized that ED patient volume and case-mix severity of illness were determinants of triage systems' performance. Therefore we decided that if a sufficient number of studies were identified, we

For peer review only - http://bmjopen.bmj.com/site/about/guidelines.xhtml

would investigate the effect of these determinants on triage systems' performance using subgroup analyses. We considered annual ED census as a marker of patient volume and the percentage of hospitalized patients as a marker of case-mix severity of illness.

Computations were carried out with SPSS Statistics version 21.0 and figures were created using Review Manager 5.3 or R version 3.2.0.[15-17]

Patient and Public Involvement

Patients and public were not involved in this study.

For peer review only

For peer review only - http://bmjopen.bmj.com/site/about/guidelines.xhtml

Results

A total of 12,684 papers were identified in the electronic search, of which sixty-six were included in the final selection (figure 1).

The majority of studies were conducted in tertiary or university hospitals (n=46; 70%) and conducted in Europe/Central Asia or North America (n=45; 68%). 49 (74%) were single-centre studies. A complete overview of the selected studies is presented in appendix 2.

44 studies (67%) had a high risk of bias in at least one domain, and 17 studies (26%) had a high risk of bias in two or more domains (figure 2 and appendix 3). The most common causes of concern were application of multiple triage systems for the same patient by the same nurse or by multiple nurses without blinding, retrospective retrieval of reference standard information without blinding for the triage outcome, and substantial amounts of missing data. In 11 studies (17%) there were concerns regarding applicability.

Triage systems

A total of 33 different triage systems were evaluated. The most commonly evaluated triage systems were the Emergency Severity Index (ESI) (n=22), the Manchester Triage System (MTS) (n=15), and the Canadian Triage and Acuity Scale (CTAS) (n=13). Other triage systems included the Taiwan Triage System (n=4), Australasian Triage Scale (n=3), South African Triage Scale (n=3), Netherlands Triage System (n=2), and Soterion Rapid Triage System (n=2). For 25 triage systems only one evaluation was published. These included 9 local or informally structured triage systems. The median sample size was 1,496 in children (range: 510 to 550,940), 1,447 in adults (range: 100 to 316,622), and 929 in elderly (range: 773 to 1,903). In total, 89 individual triage evaluations were reported: 34 (38%) in children, 52 (58%) in adults, a combination of age groups or an unspecified population, and 3 (3%) in elderly.

Reference standards

A variety of reference standards were used and the majority of studies reported multiple reference standards (Appendix 4). 12 studies used mortality at the ED as a reference standard, 13 studies ICU admission and 47 studies hospital admission. Other commonly reported reference standards were length of stay at the ED (27 studies), resource use at the ED according to the ESI criteria (14 studies),

For peer review only - http://bmjopen.bmj.com/site/about/guidelines.xhtml

expert opinion (9 studies) and costs (8 studies). Because definitions of time to mortality, resource use and expert opinion were not consistent across studies, these results could not be compared. Moreover, length of stay at the ED and costs are outcome measures that are strongly dependent on ED characteristics, and could therefore not be used to make a comparison between studies.

The most commonly evaluated triage systems

We will further restrict our analyses to the triage systems with at least three evaluations using the same reference standard. This final selection includes studies evaluating the ESI, CTAS and MTS. Characteristics of these triage systems and a summary of the available evidence are presented in table 1. The ESI, CTAS and MTS were all evaluated in settings with a different patient volume as indicated by annual hospital census, and a different case-mix as indicated by percentage of hospitalisation. For each of these triage systems, the majority of studies had risk of bias in at least one domain.

Accuracy of triage systems to identify high urgent patients

Mortality at the ED was reported in 7 evaluations of our final sample: 5 evaluations in adults, and 2 in children. Because of this low number of studies and the very low reported mortality rates (on average 0.2% in adults and <0.01% in children) it was not possible to perform comparative analyses.

ICU admission was reported in 5 evaluations in adults (2 ESI, 3 MTS) and 4 in children (3 CTAS, 1 MTS). Overall, sensitivity for ICU admission was moderate to good, ranging from 0.58 (95%CI 0.48 to 0.68) to 0.88 (95%CI 0.70 to 0.96) in adults and 0.71 (95%CI 0.66 to 0.77) to 0.93 (95%CI 0.89 to 0.95) in children. A clear difference in performance between the triage systems was not visible (figure.3).

Regardless of the triage system used, most of the ICU admitted patients were allocated to one of the two highest triage categories (Appendix 5). The exact proportion of ICU admitted patients in each triage category was highly variable, even within studies evaluating the same triage system. For example, the proportion of ICU admitted adults in MTS category 1 ranged from 21% to 79%. The number of studies, however, was too small to assess whether this variability was present in all triage systems and whether this could be explained by study or setting related factors.

For peer review only - http://bmjopen.bmj.com/site/about/guidelines.xhtml

Accuracy to identify low urgent patients

Hospital admission or discharge after the ED visit was reported as a reference standard in 14 evaluations in adults and 15 in children. Overall, specificity of the triage systems to accurately classify patients discharged home as low urgent ranged from 0.64 (95%CI 0.62 to 0.66) to 0.98 (95%CI 0.95 to 0.99) in adults and 0.69 (95%CI 0.66 to 0.72) to 0.96 (95%CI 0.94 to 0.98) in children (Figure 4). Again, sensitivities and specificities were highly variable within each of the triage systems. None of the triage systems showed a marked better specificity compared to the others.

The proportion of patients discharged after the ED visit increased from the higher to the lower urgency categories in all triage systems (Appendix 5). Again, there was a large variability within triage systems and substantial overlap between triage systems. In adults, the MTS seemed to have a higher variability compared to the other triage systems, but in children variability was greater for the CTAS.

The only additional reference standard with sufficient evaluations was resource use according to the ESI criteria (Appendix 6).

Direct comparison of triage systems

A total of 13 studies directly compared two or more triage systems. Most of these studies, however, were assessed as having a high risk of bias in the index test domain, because triage was performed by the same nurse or without blinding. Performing triage while using different triage systems sequentially, is likely to reduce the differences between triage systems. Therefore, these results should be interpreted with caution.

In ten studies, an established five-level triage system was compared with a local or informally structured triage system with three or four levels. Seven of these studies reported that the 5-level triage system provided better discrimination or better sensitivities and specificities than the local triage system and should be preferred.[18-24] One study in children found that the local triage system performed better than the established triage system (CTAS).[25]

Two studies comparing the ESI with the MTS in adults found that sensitivities and specificities for hospital admission and the prediction of mortality were largely similar.[26 27] In one of these studies, the MTS undertriaged a smaller proportion of patients compared to the ESI (8.3% versus 13.5%) at the cost of a larger proportion of “overtriage”.[26] One study in adults observed no statistically significant

For peer review only - http://bmjopen.bmj.com/site/about/guidelines.xhtml

difference between the CTAS and ESI regarding the prediction of emergency department resource utilization and immediate patient outcomes.[28] One study in children compared the ATS and ESI and found similar sensitivities and specificities for the identification of patients requiring hospital admission.[29] One single-centre study in children compared five triage systems (ATS, CTAS, ESI, MTS and a local triage system called the Ramathibodi Triage System) and concluded that the ESI showed the best validity for predicting hospital admission (AUC 0.78, 95%CI 0.74–0.81).[30] In this study, the local triage system showed the highest sensitivity (50%) and the ATS the highest specificity (94%).

Determinants of triage systems' performance

The number of studies per triage system was too small to perform subgroup analyses based on annual census or percentage hospitalisation. As an explorative analysis, we ordered all selected studies that used hospital admission as a reference standard based on annual census, and percentage hospitalisation (Appendix 7). There was no clear association between patient volume or case-mix and triage systems' sensitivity and specificity. A lower specificity for hospitals with the largest annual census and highest percentage hospitalisation could not be ruled out, but requires a larger number of studies.

For peer review only

For peer review only - http://bmjopen.bmj.com/site/about/guidelines.xhtml

Discussion

In a systematic review of sixty-six observational studies evaluating triage systems, we found that numerous different triage systems are being used but that many lack a rigorous evaluation. The most commonly used and evaluated triage systems, CTAS, ESI and MTS, show a moderate to good validity to identify high and low urgent patients. Their performance, however, is highly variable and differences in study design, study populations and reference standards make a comparison of the available evidence difficult. Although based on a limited number of studies, no clear association between patient volume and case-mix severity of illness could be found.

Strengths and weaknesses

This is the first study that evaluates the performance of triage systems in a meta-analysis. Previous reviews have merely described the results of the individual studies without synthesizing the evidence. Moreover, none of the published reviews looked at other factors that determine triage systems' performance, such as ED characteristics to compare evidence from different studies.[8-10 31-34]

Our review is based on a comprehensive search developed with a research librarian, includes duplicate assessment of eligibility and risk of bias, and duplicate data abstraction. Furthermore, the research question is based on a relevant and practical clinical issue. Triage systems are used worldwide to prioritize patients in the ED, but robust evidence on their performance is lacking.

The results of this review, however, should be interpreted taking into consideration the limitations of the underlying evidence. We included sixty-six studies in our review, but the majority of the thirty-three triage systems were evaluated by only one study. Therefore, we could only evaluate three most frequent used triage systems: CTAS, ESI and MTS. Even for these commonly used triage systems, few evaluations were available due to the variety of reference standards. Although the triage evaluations included the whole age spectrum, studies targeted at elderly patients were scarce. It is important to evaluate triage systems' performance separately for the most vulnerable populations at the ED, specifically children and elderly. In these patient groups the spectrum of disease, the presence of non-specific signs and symptoms and progression of disease course differs from that in adult patients.

We are not aware of any (randomized) controlled trial that investigates the effect of triage on patient outcome. Therefore we conducted a systematic review of observational studies. Comparing

For peer review only - http://bmjopen.bmj.com/site/about/guidelines.xhtml

observational studies is challenging because the effect of a triage system cannot be assessed independently of its context. Likely, other factors such as ED and hospital characteristics or local practices and training have influenced the results of the included studies. We chose to display the results of different studies in a forest plot. Because of the limited number of studies and heterogeneity of the study populations, it is difficult to compare the results from different triage systems and these plots should be interpreted with caution.

We aimed to explore heterogeneity between studies and more specifically the effect of differences in patient load and case-mix severity of illness. Unfortunately, due to the small number of studies per triage systems, we could not draw strong conclusions about the relation between these factors and triage systems' performance. There are more potential factors that could affect triage systems' validity, such as the local infrastructure, the experience and training of the triage nurse, the presence of a computerized triage application or variations in disease epidemiology. Moreover, since most included studies had risk of bias in at least one domain, we cannot rule out that study design and methodological quality have led to heterogeneity of the results as well.

We predefined mortality at the ED and ICU admission as reference standards for high patient urgency and discharge home as a reference standard for low patient urgency. Due to the relatively low number of studies reporting mortality and the low mortality rate of patients in the ED, we could not use it as a reference standard. ICU and hospital admission are feasible reference standards for large study populations, and theoretically, criteria for ICU and hospital admission should be reasonably comparable between settings. It is possible, however, that ED and hospital characteristics and local practices result in differences in the decision to admit a patient between EDs.

We restricted our review to triage systems in high and higher-middle income countries. We applied this selection because EDs in lower income countries have their own unique characteristics and challenges. Several recently published studies have addressed triage in low-income settings.[35 36]

Implications and future research

Our review identified thirty-tree triage systems for which at least one evaluation was published. Probably, there are more triage systems in use, which are not formally evaluated. There are several advantages, not addressed in this review, of using an established triage system over a local triage scale. Beside that performance of the most commonly used triage systems is known, they have a formal

For peer review only - http://bmjopen.bmj.com/site/about/guidelines.xhtml

governance structure and undergo regular updates. Moreover, there are standardized implementation guidelines and training programs available.[37-39] The CTAS, ESI and MTS all show a reasonable performance for triage at the ED. Our results do not suggest that one of the established triage systems should be preferred over the other.

Our review indicates that large variation of performance exists even in studies assessing the same triage system. This suggests that other factors influence triage systems' performance. Consequently, generalizability of individual studies evaluating a triage system is low and a study on triage validity in one setting may not apply to a setting with different characteristics or in a different health care system. Our review demonstrates that the majority of studies evaluating triage systems were conducted in a single-centre. Furthermore, most multicentre studies provided only pooled results. Yet, multicentre studies using similar study designs and reference standard definitions are needed to evaluate the range of triage systems' performance in different settings. More importantly, these studies can provide valuable insights in determinants of triage systems' performance and areas of improvement.

The extensive use of triage systems in clinical practice contrasts with the limited number of studies evaluating their performance. Triage systems were typically developed based on expert opinion and implemented out of clinical necessity. They are mostly used in their country or region of origin: the MTS is widely used in the United Kingdom and Europe, the ESI in the United States, and the CTAS in Canada and in French-speaking countries.[40] Since most EDs already have experience with a certain triage system and some triage systems are recommended by national guidelines it could be worthwhile shifting away the focus on triage systems' performance towards improvement of the established triage systems. Our review suggests that there is room for improvement of all triage systems regarding both the correct identification of high urgent and low urgent patients.

The most commonly used triage systems, CTAS, ESI and MTS, have a reasonable validity for the triage of patients at the ED. Important research questions that remain are what determinants influence a triage systems' performance and how the performance of existing triage systems can be improved.

Acknowledgements

We thank Wichor Bramer, biomedical information specialist in Erasmus University Medical Centre, Rotterdam, for his help with developing the search strategy

For peer review only - http://bmjopen.bmj.com/site/about/guidelines.xhtml

Authors' contributions

JMZ and VvdH contributed equally to this work. All authors substantially contributed to the conception and design of the study and interpretation of the findings. JMZ, KMJ and HAM conceived the study idea. JMZ, VvdH, NS, MvV conducted the systematic review including screening of abstracts and full text and risk of bias assessment. JMZ and VvdH extracted data, performed the analyses and wrote the first draft of the manuscript. All authors revised it critically for important intellectual content and gave their approval of the final version. All authors had full access to all of the data (including statistical reports and tables) in the study and can take responsibility for the integrity of the data and the accuracy of the data analysis. HAM is guarantor.

For peer review only

For peer review only - http://bmjopen.bmj.com/site/about/guidelines.xhtml

References

1. Blunt I, Edwards N, Merry L. What's behind the A&E 'crisis'? *Briefing* 2015.
2. Australasian College for Emergency Medicine. Access Block and Overcrowding in Emergency departments. , 2004.
3. Hospital-based emergency care: at the breaking point. ; 2013.
4. Mackway-Jones K, Marsden J, Windle J. Emergency triage. London: BMJ 2006.
5. Warren DW, Jarvis A, LeBlanc L, et al. Revisions to the Canadian Triage and Acuity Scale paediatric guidelines (PaedCTAS). *Cjem* 2008;10(3):224-43.
6. Gilboy NT, P.; Travers, D.A.; Rosenau, A.M.; Eitel, D.R. Emergency Severity Index, Version 4: Implementation Handbook. Rockville, MD: Agency for Healthcare Research and Quality 2005.
7. Australian College for Emergency Medicine. Guidelines on the implementation of the australasian triage scale in emergency departments. http://www.acem.org.au/media/policies_and_guidelines/G24_Implementation__ATS.pdf [
8. Christ M, Grossmann F, Winter D, et al. Modern triage in the emergency department. *Dtsch Arztebl* 2010;107(50):892-98.
9. Farrohknia N, Castren M, Ehrenberg A, et al. Emergency department triage scales and their components: a systematic review of the scientific evidence. *Scand J Trauma Resusc Emerg Med* 2011;19:42.
10. Parenti N, Ferrara L, Bacchi Reggiani ML, et al. Reliability and validity of two four-level emergency triage systems. *Eur J Emerg Med* 2009;16(3):115-20.
11. Stroup DF, Berlin JA, Morton SC, et al. Meta-analysis of observational studies in epidemiology: a proposal for reporting. Meta-analysis Of Observational Studies in Epidemiology (MOOSE) group. *Jama* 2000;283(15):2008-12.
12. World Bank Group. World Bank Country and Lending Groups. https://datahelpdesk.worldbank.org/knowledgebase/articles/906519-world-bank-country-and-lending-groups. Accessed October 5th, 2017.
13. Whiting PF, Rutjes AW, Westwood ME, et al. QUADAS-2: a revised tool for the quality assessment of diagnostic accuracy studies. *Ann Intern Med* 2011;155(8):529-36.
14. Kuriyama A, Urushidani S, Nakayama T. Five-level emergency triage systems: variation in assessment of validity. *Emerg Med J* 2017
15. R: A language and environment for statistical computing [program]. Vienna, Austria: R Foundation for Statistical Computing, 2008.
16. IBM SPSS Statistics for Windows [program]. 21 version. Armonk, NY 2011.
17. Review Manager (RevMan) [program]. 5.3 version. Copenhagen: The Nordic Cochrane Centre, The Cochrane Collaboration, 2014.
18. Chang YC, Ng CJ, Wu CT, et al. Effectiveness of a five-level Paediatric Triage System: An analysis of resource utilisation in the emergency department in Taiwan. *Emerg Med J* 2012
19. Chi CH, Huang CM. Comparison of the Emergency Severity Index (ESI) and the Taiwan triage system in predicting resource utilization. *J Formos Med Assoc* 2006;105(8):617-25.

For peer review only - http://bmjopen.bmj.com/site/about/guidelines.xhtml

20. Maleki M, Fallah R, Riahi L, et al. Effectiveness of Five-Level Emergency Severity Index Triage System Compared With Three-Level Spot Check: An Iranian Experience. *Arch Trauma Res* 2015;4(4):e29214.
21. Ng CJ, Hsu KH, Kuan JT, et al. Comparison between Canadian Triage and Acuity Scale and Taiwan Triage System in emergency departments. *J Formos Med Assoc* 2010;109(11):828-37.
22. Ng CJ, Yen ZS, Tsai JCH, et al. Validation of the Taiwan triage and acuity scale: A new computerised five-level triage system. *Emerg Med J* 2011;28(12):1026-31.
23. Yuksen C, Sawatmongkornkul S, Suttabuth S, et al. Emergency severity index compared with 4-level triage at the emergency department of Ramathibodi University Hospital. *Asian Biomed* 2016;10(2):155-61. doi: 10.5372/1905-7415.1002.477
24. Travers DA, Waller AE, Bowling JM, et al. Five-level triage system more effective than three-level in tertiary emergency department. *J Emerg Nurs* 2002;28(5):395-400.
25. Gouin S, Gravel J, Amre DK, et al. Evaluation of the Paediatric Canadian Triage and Acuity Scale in a pediatric ED. *Am J Emerg Med* 2005;23(3):243-47.
26. Storm-Versloot MN, Ubbink DT, Kappelhof J, et al. Comparison of an informally structured triage system, the emergency severity index, and the manchester triage system to distinguish patient priority in the emergency department. *Acad Emerg Med* 2011;18(8):822-29.
27. van der Wulp I, Schrijvers AJP, van Stel HF. Predicting admission and mortality with the Emergency Severity Index and the Manchester Triage System: a retrospective observational study. *Emergency Medicine Journal* 2009;26(7):506-09.
28. Worster A, Fernandes CM, Eva K, et al. Predictive validity comparison of two five-level triage acuity scales. *Eur J Emerg Med* 2007;14(4):188-92.
29. Ghafarypour-Jahrom M, Taghizadeh M, Heidari K, et al. Validity and Reliability of the Emergency Severity Index and Australasian Triage System in Pediatric Emergency Care of Mofid Children's Hospital in Iran. *Bull Emerg Trauma* 2018;6(4):329-33.
30. Aeimchanbanjong K, Pandee U. Validation of different pediatric triage systems in the emergency department. *World J Emerg Med* 2017;8(3):223-27.
31. Azeredo TR, Guedes HM, Rebelo de Almeida RA, et al. Efficacy of the Manchester Triage System: a systematic review. *Int Emerg Nurs* 2015;23(2):47-52.
32. Souza CC, Araujo FA, Chianca TC. [Scientific Literature on the Reliability and Validity of the Manchester Triage System (MTS) Protocol: A Integrative Literature Review]
Producao cientifica sobre a validade e confiabilidade do Protocolo de Manchester: revisao integrativa da literature. *Rev Esc Enferm USP* 2015;49(1):144-51.
33. van Veen M, Moll HA. Reliability and validity of triage systems in paediatric emergency care. *Scand J Trauma Resusc Emerg Med* 2009;17:38.
34. de Magalhaes-Barbosa MC, Robaina JR, Prata-Barbosa A, et al. Validity of triage systems for paediatric emergency care: a systematic review. *Emerg Med J* 2017
35. Hansoti B, Jenson A, Keefe D, et al. Reliability and validity of pediatric triage tools evaluated in Low resource settings: a systematic review. *BMC Pediatr* 2017;17(1):37.
36. Jenson A, Hansoti B, Rothman R, et al. Reliability and validity of emergency department triage tools in low- and middle-income countries: a systematic review. *Eur J Emerg Med* 2018;25(3):154-60.
37. www.esitriage.com. Accessed October 5th, 2017. .

For peer review only - http://bmjopen.bmj.com/site/about/guidelines.xhtml

38. http://caep.ca/resources/ctas. Accessed October 5th, 2017.
39. www.triagenet.net. Accessed October 5th, 2017. [accessed October 5th 2017.
40. Moll HA. Challenges in the validation of triage systems at emergency departments. *J Clin Epidemiol* 2010;63(4):384-88.

For peer review only

For peer review only - http://bmjopen.bmj.com/site/about/guidelines.xhtml

Tables and Figure captions

Table 1. Evidence summary of the most commonly used triage systems

	**Canadian Triage And Acuity Scale (CTAS)**	**Emergency Severity Index (ESI)**	**Manchester Triage System (MTS)**
Triage system characteristics			
Description	List, based on presenting signs and symptoms	Flowchart, based on physical signs and expected resource use	Multiple flowcharts, based on presentational signs and symptoms
Number of levels	5	5	5
Classification and waiting time	Level I, Immediate<br>Level II, 15 min<br>Level III, 30 min<br>Level IV, 60 min<br>Level V, 2 hrs	Immediate<br>Emergent, 14 min<br>Urgent, 60 min<br>Semi-urgent, 2 hrs<br>Non-urgent, 24 hrs	Immediate<br>Very urgent, 10 min<br>Urgent, 60 min<br>Standard, 2 hrs<br>Non-urgent, 4 hrs
Quantity of evidence			
Total number of evaluations	13	21	15
Evaluations in children	9	4	7
Evaluations in elderly	1	2	0
Diversity of evidence (range)			
Number of hospitals per study	1-12	1-7	1-4
Inclusions per study	481-550,940	180-37,974	872-31,622
Hospital census	10,000-75,000	10,000-90,000	7,000-190,000
Hospitalisation rate	8%-47%	10%-62%	5-33%
Risk of bias			
High risk of bias in at least one domain	54%	81%	67%
High risk of bias in >1 domain	15%	23%	13%

For peer review only - http://bmjopen.bmj.com/site/about/guidelines.xhtml

Figure 1. Flow diagram of study selection process

Figure 2. Risk of bias of included studies

Figure 3. Sensitivity and specificity of triage systems for identifying high urgency patients as defined by ICU admission

CTAS = Canadian Triage and Acuity Scale; TP = true positive; FP = false positive; FN = false negative; TN = true negative; ESI = Emergency Severity Index; MTS = Manchester Triage System

Figure 4. Sensitivity and specificity of triage systems for identifying low urgency patients as defined by discharge home after the ED visit

CTAS = Canadian Triage and Acuity Scale; TP = true positive; FP = false positive; FN = false negative; TN = true negative; ESI = Emergency Severity Index; MTS = Manchester Triage System

For peer review only - http://bmjopen.bmj.com/site/about/guidelines.xhtml

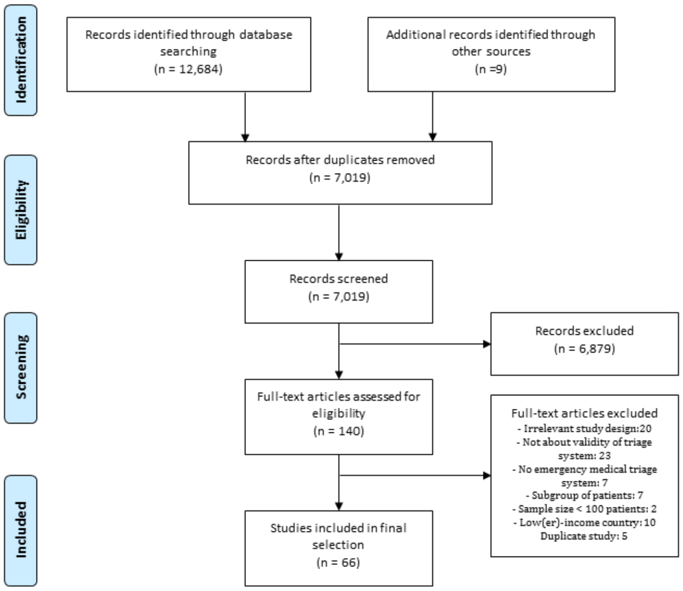

Figure 1. Flow diagram of study selection process

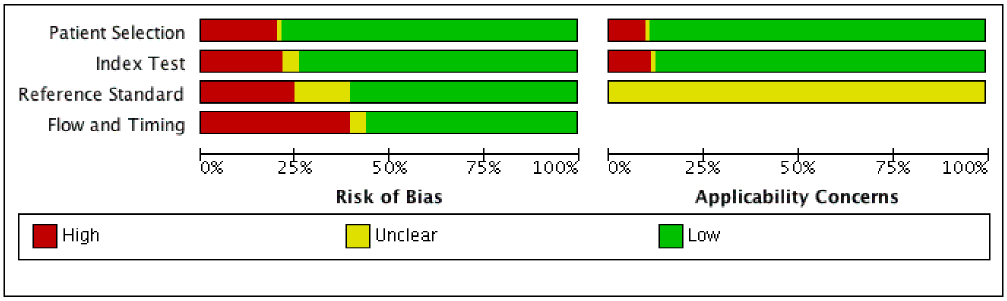

Figure 2. Risk of bias of included studies

241x70mm (72 x 72 DPI)

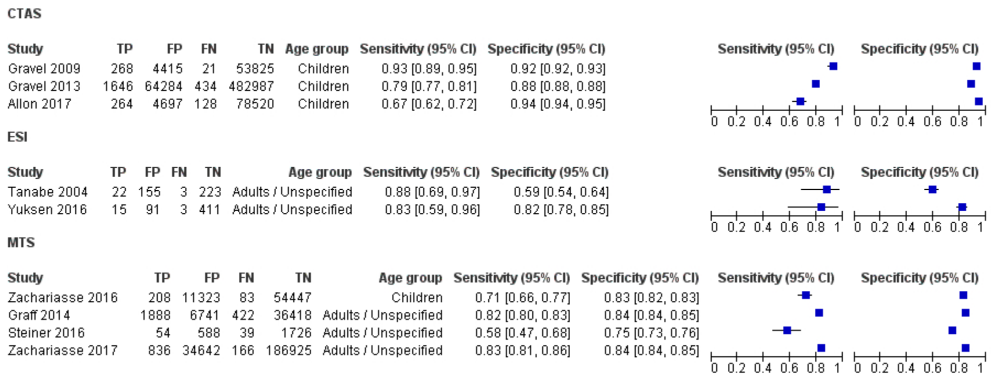

Figure 3. Sensitivity and specificity of triage systems for identifying high urgency patients as defined by ICU admission
CTAS = Canadian Triage and Acuity Scale; TP = true positive; FP = false positive; FN = false negative; TN = true negative; ESI = Emergency Severity Index; MTS = Manchester Triage System

320x136mm (72 x 72 DPI)

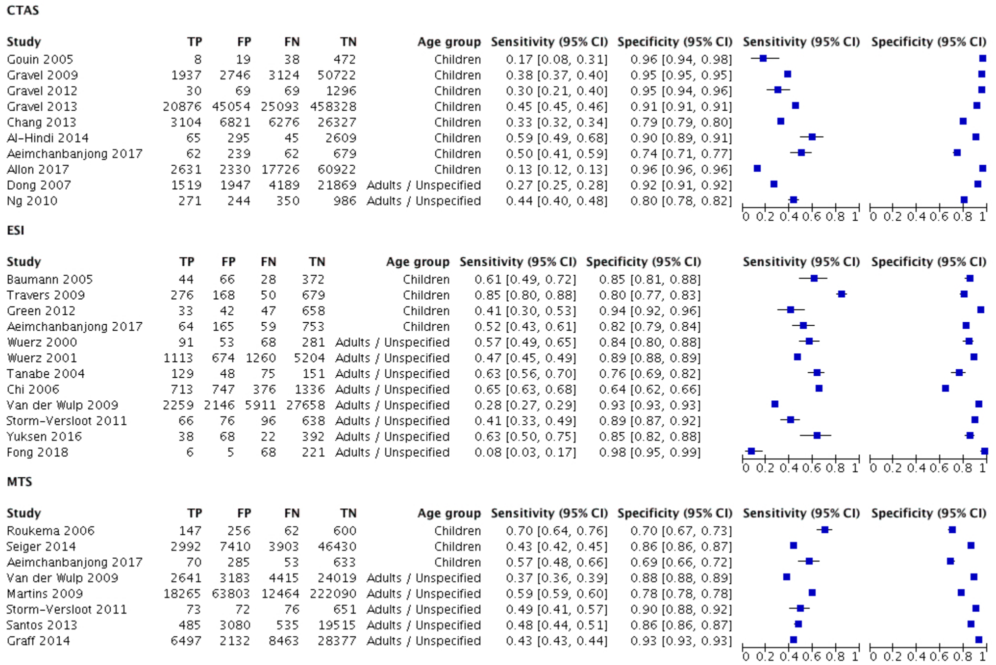

CTAS

Study	TP	FP	FN	TN	Age group	Sensitivity (95% CI)	Specificity (95% CI)
Gouin 2005	8	19	38	472	Children	0.17 [0.08, 0.31]	0.96 [0.94, 0.98]
Gravel 2009	1937	2746	3124	50722	Children	0.38 [0.37, 0.40]	0.95 [0.95, 0.95]
Gravel 2012	30	69	69	1296	Children	0.30 [0.21, 0.40]	0.95 [0.94, 0.96]
Gravel 2013	20876	45054	25093	458328	Children	0.45 [0.45, 0.46]	0.91 [0.91, 0.91]
Chang 2013	3104	6821	6276	26327	Children	0.33 [0.32, 0.34]	0.79 [0.79, 0.80]
Al-Hindi 2014	65	295	45	2609	Children	0.59 [0.49, 0.68]	0.90 [0.89, 0.91]
Aeimchanbanjong 2017	62	239	62	679	Children	0.50 [0.41, 0.59]	0.74 [0.71, 0.77]
Allon 2017	2631	2330	17726	60922	Children	0.13 [0.12, 0.13]	0.96 [0.96, 0.96]
Dong 2007	1519	1947	4189	21869	Adults / Unspecified	0.27 [0.25, 0.28]	0.92 [0.91, 0.92]
Ng 2010	271	244	350	986	Adults / Unspecified	0.44 [0.40, 0.48]	0.80 [0.78, 0.82]

ESI

Study	TP	FP	FN	TN	Age group	Sensitivity (95% CI)	Specificity (95% CI)
Baumann 2005	44	66	28	372	Children	0.61 [0.49, 0.72]	0.85 [0.81, 0.88]
Travers 2009	276	168	50	679	Children	0.85 [0.80, 0.88]	0.80 [0.77, 0.83]
Green 2012	33	42	47	658	Children	0.41 [0.30, 0.53]	0.94 [0.92, 0.96]
Aeimchanbanjong 2017	64	165	59	753	Children	0.52 [0.43, 0.61]	0.82 [0.79, 0.84]
Wuerz 2000	91	53	68	281	Adults / Unspecified	0.57 [0.49, 0.65]	0.84 [0.80, 0.88]
Wuerz 2001	1113	674	1260	5204	Adults / Unspecified	0.47 [0.45, 0.49]	0.89 [0.88, 0.89]
Tanabe 2004	129	48	75	151	Adults / Unspecified	0.63 [0.56, 0.70]	0.76 [0.69, 0.82]
Chi 2006	713	747	376	1336	Adults / Unspecified	0.65 [0.63, 0.68]	0.64 [0.62, 0.66]
Van der Wulp 2009	2259	2146	5911	27658	Adults / Unspecified	0.28 [0.27, 0.29]	0.93 [0.93, 0.93]
Storm-Versloot 2011	66	76	96	638	Adults / Unspecified	0.41 [0.33, 0.49]	0.89 [0.87, 0.92]
Yuksen 2016	38	68	22	392	Adults / Unspecified	0.63 [0.50, 0.75]	0.85 [0.82, 0.88]
Fong 2018	6	5	68	221	Adults / Unspecified	0.08 [0.03, 0.17]	0.98 [0.95, 0.99]

MTS

Study	TP	FP	FN	TN	Age group	Sensitivity (95% CI)	Specificity (95% CI)
Roukema 2006	147	256	62	600	Children	0.70 [0.64, 0.76]	0.70 [0.67, 0.73]
Seiger 2014	2992	7410	3903	46430	Children	0.43 [0.42, 0.45]	0.86 [0.86, 0.87]
Aeimchanbanjong 2017	70	285	53	633	Children	0.57 [0.48, 0.66]	0.69 [0.66, 0.72]
Van der Wulp 2009	2641	3183	4415	24019	Adults / Unspecified	0.37 [0.36, 0.39]	0.88 [0.88, 0.89]
Martins 2009	18265	63803	12464	222090	Adults / Unspecified	0.59 [0.59, 0.60]	0.78 [0.78, 0.78]
Storm-Versloot 2011	73	72	76	651	Adults / Unspecified	0.49 [0.41, 0.57]	0.90 [0.88, 0.92]
Santos 2013	485	3080	535	19515	Adults / Unspecified	0.48 [0.44, 0.51]	0.86 [0.86, 0.87]
Graff 2014	6497	2132	8463	28377	Adults / Unspecified	0.43 [0.43, 0.44]	0.93 [0.93, 0.93]

Figure 4. Sensitivity and specificity of triage systems for identifying low urgency patients as defined by discharge home after the ED visit

CTAS = Canadian Triage and Acuity Scale; TP = true positive; FP = false positive; FN = false negative; TN = true negative; ESI = Emergency Severity Index; MTS = Manchester Triage System

359x255mm (72 x 72 DPI)

Appendix 1. Search strategy

Embase

(triage* OR ((acuity OR severit* OR priorit* OR critical*) NEAR/3 (scale* OR level* OR index* OR score* OR measure* OR assessment*))):ab,ti AND ('emergency ward'/de OR 'emergency medicine'/de OR 'emergency care'/exp OR (((emergen* OR acute) NEAR/3 (department* OR ward* OR unit* OR room* OR care))):ab,ti) AND (reliability/exp OR reproducibility/de OR 'validation process'/de OR 'validation study'/de OR accuracy/de OR 'sensitivity and specificity'/exp OR 'diagnostic accuracy'/de OR evaluation/de OR validity/exp OR (reliab* OR reproducib* OR validation OR validaty OR consisten* OR variabilit* OR accura* OR 'intra observer' OR intraobserver OR sensitivity OR specificity):ab,ti OR evaluat*:ti) AND [english]/lim

Medline Ovid

(Triage/ OR (triage* OR ((acuity OR severit* OR priorit* OR critical*) ADJ3 (scale* OR level* OR index* OR score* OR measure* OR assessment*))).ab,ti.) AND (exp "Emergency Service, Hospital"/ OR "emergency medicine"/ OR "emergency care"/ OR (((emergen* OR acute) ADJ3 (department* OR ward* OR unit* OR room* OR care))).ab,ti.) AND (Reproducibility of Results/ OR Validation Studies.pt. OR exp "sensitivity and specificity"/ OR Evaluation Studies.pt. OR (reliab* OR reproducib* OR validation OR validaty OR consisten* OR variabilit* OR accura* OR "intra observer" OR intraobserver OR sensitivity OR specificity).ab,ti. OR evaluat*.ti.) AND english.la.

Cochrane central

(triage* OR ((acuity OR severit* OR priorit* OR critical*) NEAR/3 (scale* OR level* OR index* OR score* OR measure* OR assessment*))):ab,ti AND ((((emergen* OR acute) NEAR/3 (department* OR ward* OR unit* OR room* OR care))):ab,ti) AND ((reliab* OR reproducib* OR validation OR validaty OR consisten* OR variabilit* OR accura* OR 'intra observer' OR intraobserver OR sensitivity OR specificity):ab,ti OR evaluat*:ti)

Web-of-science

TS=(triage* OR ((acuity OR severit* OR priorit* OR critical*) NEAR/3 (scale* OR level* OR index* OR score* OR measure* OR assessment*))) AND TS=((((emergen* OR acute) NEAR/3 (department* OR ward* OR unit* OR room* OR care)))) AND (TS=(reliab* OR reproducib* OR validation OR validaty OR consisten* OR variabilit* OR accura* OR "intra observer" OR intraobserver OR sensitivity OR specificity) OR TI=(evaluat*)) AND LA=(English)

CINAHL

(MH Triage+ OR (triage* OR ((acuity OR severit* OR priorit* OR critical*) N3 (scale* OR level* OR index* OR score* OR measure* OR assessment*)))) AND (MH "Emergency Service"+ OR MH "emergency medicine"+ OR (((emergen* OR acute) N3 (department* OR ward* OR unit* OR room* OR care)))) AND (MH Reproducibility of Results+ OR MH Validity+ OR MH "sensitivity and specificity"+ OR MH Evaluation+ OR (reliab* OR reproducib* OR validation OR validaty OR consisten* OR variabilit* OR accura* OR "intra observer" OR intraobserver OR sensitivity OR specificity) OR TI (evaluat*))

Google Scholar

triage emergency|emergencies
reliability|reproducibility|validation|validity|consistency|accuracy|sensitivity|specificity|interobserver |intraobserver

For peer review only

Appendix 2. Selected studies

Table 2.1 Characteristics of included studies

Study	Setting	Annual census	Hospitalization rate	Study population	Triage System	Reference standard (data available)
Aeimchanbanjong, 2017[1]	Single academic hospital, Thailand	10,000	12%	1041 children	ATS, CTS, ESI, MTS, Ramathibodi Triage System	- Hospital admission
Al-Hindi, 2014[2]	Single tertiary maternity and children's hospital, Saudi Arabia	155,000	4%	3,014 children	Ped-CTAS	- Hospital admission<br>- Resource use, other<br>- Waiting time to physician
Allon, 2017[3]	Single tertiary children's hospital, Israel	-	24%	83,609 children	Ped-CTAS	- Hospital admission<br>- ICU admission<br>- Length of stay at the ED<br>- Left without being seen
Baumann, 2005[4]	Single academic hospital, USA	53,000	14%	510 children	ESI v.3	- Hospital admission<br>- Resource use based on ESI<br>- Length of stay at the ED<br>- Mortality at the ED<br>- Mortality after 1 year<br>- Left without being seen
Baumann, 2006[5]	Single academic hospital, USA	53,000	43%	929 elderly	ESI v.3	- Hospital admission<br>- ICU admission<br>- Resource use based on ESI<br>- Length of stay at the ED<br>- Mortality at the ED<br>- Mortality after 1 year<br>- Left without being seen
Brillman, 1996[6]	Single academic hospital,	65,000	6%	5,106 patients	Automated Military	- Hospital admission<br>- Expert opinion

For peer review only

	USA				Outpatient System	
Bruijns, 2008[7]	Single community hospital, South Africa	54,000	40%	798 adults	Cape Triage Score	- Hospital admission<br>- Mortality at the ED
Chang, 2012[8]	Single academic hospital, Taiwan	-	23%	42,346 children	CTAS and Ped-TTS	- Hospital admission<br>- Costs
Chi, 2006[9]	Single academic hospital, Taiwan	58,000	34%	3,172 adults	ESI v.2 and TTS	- Hospital admission<br>- Length of stay at the ED
Dong, 2007[10]	Single tertiary hospital, Canada	67,000	19%	29,524 adults	CTAS	- Hospital admission<br>- Resource use, other<br>- Length of stay at the ED<br>- Mortality at the ED<br>- Costs
Dugas, 2016[11]	Sample of ED visits from 356 hospitals, USA	-	14%	25,198 adults	Combination of triage systems and Electronic emergency Triage System	- Hospital admission<br>- ICU admission<br>- Resource use, other<br>- Resource use based on ESI<br>- Composite outcome: in-hospital mortality, intensive care unit admission or transfer to operating room or catheterization suite
Eitel, 2003[12]	7 community hospitals, USA	28,000-90,000	34% (mean)	1,042 patients	ESI v.2	- Hospital admission<br>- Resource use based on ESI<br>- Length of stay at the ED<br>- Mortality: 60-day mortality
Engan, 2018[13]	Single academic hospital, Norway	4,750	44%	782 children	modified version of the pediatric SATS	- Hospital admission<br>- Resource use, other
Erimsah, 2015[14]	Single academic hospital,	40,000	16%	618 patients	Ministry of Health of	- Hospital admission<br>- Resource use, other

For peer review only

	Turkey				Turkey's mandatory emergency triage instrument	- Length of stay at the ED<br>- Expert opinion<br>- Immediate lifesaving interventions<br>- Mortality: in-hospital mortality
Fan, 2013[15]	Single tertiary hospital,<br>China (Hong Kong)	128,000	-	100 children and adults	Hong Kong Accident and Emergency Triage Guidelines 4th version	- Expert opinion
Fong, 2018[16]	Single community hospital, Singapore	-	25%	300 adults	ESI and Patient Acuity Category Scale	- Hospital admission<br>- Resource use based on ESI
Funakoshi, 2016[17]	Single community hospital,<br>Japan	31,793	-	17,121 adults	Japanese Triage and Acuity Scale	- Hospital admission<br>- Length of stay at the ED<br>- Mortality at the ED
Ganapathy, 2018[18]	Single community hospital, Singapore	-	17%	172,933 children	Singapore Paediatric Triage Scale	- Hospital admission<br>- ICU admission<br>- Length of stay at the ED<br>- Length of stay in hospital
Van Gerven, 2001[19]	Single academic hospital,<br>Belgium	-	45%	3,650 children and adults	Australian National Triage Scale	- Hospital admission<br>- Sentinel diagnosis
Ghafarypour-Jahrom, 2018[20]	Single academic hospital, Iran	45,000	-	1,300 children	ATS and ESI	- Hospital admission
Gouin, 2005[21]	Single academic children's hospital,<br>Canada	65,000	9%	1,281 children	CTAS and local triage tool	- Hospital admission<br>- Resource use, other<br>- PRISA score
Gräff, 2014[22]	Single academic hospital,<br>Germany	23,000	33%	45,469 children and adults	MTS	- Hospital admission<br>- ICU admission<br>- Resource use, other

For peer review only

						- Length of stay at the ED<br>- Mortality: in-hospital mortality<br>- Length of stay in hospital<br>- Level of prehospital care
Gravel, 2009[23]	Single academic hospital, Canada	60,000	9%	5,829 children	Ped-CTAS	- Hospital admission<br>- ICU admission<br>- Length of stay at the ED<br>- Mortality at the ED
Gravel, 2012[24]	9 academic hospitals, Canada	43,962	8%	1,464 children	CTAS	- Hospital admission<br>- Resource use, other<br>- Length of stay at the ED
Gravel, 2013[25]	12 academic children's hospitals, Canada	25,000-75000	9%	550,940 children	CTAS	- Hospital admission<br>- ICU admission<br>- Length of stay at the ED<br>- Left without being seen
Green, 2012[26]	Single academic children's hospital, USA	45,000	10%	780 children	ESI v.4	- Hospital admission<br>- Resource use based on ESI<br>- Length of stay at the ED
Hansen, 2017[27]	Single hospital, Denmark	-	-	550 children	Danish Regions Pediatric Triage model	- Composite outcome "van Veen"; based on abnormal vital parameters, life-threatening conditions, diagnostics, interventions and follow-up<br>- Composite outcome based on life threatening condition, transfer to higher hospital, ICU, or hospital admission
Hay, 2001[28]	Single hospital, Israel	-	-	2,886 patients	Local triage system	- Expert opinion<br>- Waiting time to physician
Van Ierland, 2011[29]	Single academic hospital, the Netherlands	20,000	30% (adults) and 15% (children)	3,207 adults and 701 children	Netherlands Triage System	- Hospital admission<br>- ICU admission<br>- Resource use, other<br>- Follow-up at outpatient clinic or GP

For peer review only

Jobe, 2013[30]	Single academic hospital, Belgium	42,000	36%	544 children and adults	Echelle Liégeoise d'Index de Sévérité à l'Admission	- Hospital admission<br>- ICU admission<br>- Resource use, other<br>- Resource use based on ESI<br>- Mortality at the ED
Lee, 2003[31]	4 hospitals, China (Hong Kong)	-	-	2,410 patients	5-level triage scale	- Expert opinion
Lee, 2011[32]	Single academic hospital, South Korea	60,000	47%	1,903 elderly	CTAS, revised edition	- Hospital admission<br>- ICU admission<br>- Resource use, other<br>- Mortality at the ED<br>- Costs<br>- Immediate lifesaving interventions<br>- Mortality: in-hospital mortality<br>- Length of stay in hospital
Lin, 2016[33]	Single academic hospital, China	217,000	-	275,378 children	Chinese pediatric emergency triage system	- Expert opinion<br>- Waiting time for care<br>- Patients' parents' satisfaction
Ma, 2008[34]	Single academic children's hospital, Canada	50,000	-	1,618 children	Ped-CTAS	- Costs
Maleki, 2015[35]	Single teaching hospital, Iran	74,000	-	387 patients	ESI v.4 and Spot check triage system	- Waiting time to nurse<br>- Waiting time to physician<br>- Waiting time to first lab results
Maningas, 2006[36]	Single community hospital, USA	57,000	6%	7,077 children	Soterion Rapid Triage System	- Hospital admission<br>- Length of stay at the ED<br>- Costs<br>- CPT codes
Maningas, 2006[37]	Single community hospital, USA	57,000	10%	33,850 adults	Soterion Rapid Triage System	- Hospital admission<br>- Length of stay at the ED<br>- Costs<br>- CPT codes

For peer review only

Martins, 2009[38]	Single community hospital, Portugal	128,000	10%	316,622 adults	MTS	- Hospital admission<br>- Mortality at the ED
Meyer, 2018[39]	Single community hospital, South Africa	-	22%	4,002 patients	SATS	- Hospital admission
Ng, 2010[40]	3 hospitals, Taiwan	-	34%	1,851 adults	CTAS and Taiwan Triage System	- Hospital admission<br>- Length of stay at the ED<br>- Costs
Ng, 2011[41]	11 academic hospitals, 18 regional hospitals and 4 district hospitals, Taiwan	95,912 (average, university hospitals); 47,056 (average regional hospitals); 23,789 (average district hospitals	30%	10,533 adults	Taiwan Triage and Acuity Scale and Taiwan Triage System	- Hospital admission<br>- Resource use based on ESI<br>- Length of stay at the ED
Platts-Mills, 2010[42]	Single academic hospital, USA	64,000	58%	773 elderly	ESI v.4	- Immediate lifesaving interventions
Roukema, 2006[43]	Single teaching hospital, the Netherlands	30,000	-	1,065 children	MTS	- Hospital admission<br>- Resource use based on ESI<br>- Composite outcome “van Veen”; based on abnormal vital parameters, life-threatening conditions, diagnostics, interventions and follow-up
Santos, 2013[44]	Single hospital, Portugal	-	5%	24,721 adults	MTS v.2	- Hospital admission<br>- Resource use, other<br>- Mortality: in-hospital mortality

Seiger, 2014[45]	2 academic and 2 teaching hospitals, the Netherlands, UK and Portugal	9000-60,000	11% (average)	60,735 children	MTS	- Hospital admission
Steiner, 2016[46]	Single tertiary hospital, Switzerland	-	-	2,407 adults	MTS	- ICU admission<br>- Expert opinion<br>- Mortality: 30 day mortality<br>- Length of stay in hospital
Storm-Versloot, 2011[47]	Single academic hospital, the Netherlands	31,000	18%	876 patients	ESI, MTS and informally structured triage system	- Hospital admission<br>- Resource use based on ESI<br>- Length of stay at the ED<br>- Expert opinion
Storm- Versloot, 2014[48]	Single academic hospital, the Netherlands	31,000	21%	906 patients	MTS and informally structured triage system	- Length of stay at the ED<br>- Waiting time from arrival to treatment room<br>- Waiting time from treatment room to discharge from ED<br>- Patient satisfaction
Taboulet, 2009[49]	Single teaching hospital, France	30,000	18%	941 adults	FRench Emergency Nurses Classification in Hospital Scale v.2	- Hospital admission<br>- Resource use, other<br>- Waiting time to physician
Tanabe, 2004[50]	Single academic hospital, USA	70,000	-	403 children and adults	ESI v.3	- Resource use, other<br>- Length of stay at the ED<br>- Length of stay in hospital
Tanabe, 2004[51]	Single academic hospital, USA	70,000	-	403 children and adults	ESI v.3	- Hospital admission<br>- Resource use based on ESI<br>- Length of stay at the ED
Travers, 2002[52]	Single academic hospital, USA	-	-	305 patients	ESI and 3-level system	- Expert opinion
Travers, 2009[53]	1 academic hospital	13,838-	-	1,173	ESI v.4	- Hospital admission

For peer review only

	and 4 teaching/community hospitals, USA	44,987		children		- Resource use based on ESI<br>- Length of stay at the ED
Twomey, 2013[54]	1 private and 5 public community hospitals, South Africa	-	26%	2,014 children	revised paediatric SATS	- Hospital admission
Veen, van, 2008[55]	1 academic children's and 1 teaching hospital, the Netherlands	9,000-30,000	-	13,554 children	MTS v.1	- Composite outcome "van Veen"; based on abnormal vital parameters, life-threatening conditions, diagnostics, interventions and follow-up
Veen, van, 2012[56]	1 academic children's and 1 teaching hospital, the Netherlands	9,000-15,000	-	11,260 children	MTS incl. modifications for children with fever	- Composite outcome "van Veen"; based on abnormal vital parameters, life-threatening conditions, diagnostics, interventions and follow-up
Waldrop, 1997[57]	Single teaching hospital, USA	65,000	5% (adults), 8% (children)	13,294 adults and 1528 children	Local triage system	- Hospital admission<br>- Mortality at the ED<br>- Length of stay at the ED<br>- Left without being seen<br>- Waiting time to examination
Worster, 2007[58]	2 academic hospitals, Canada	36,000-395,000	-	481 patients	CTAS and ESI	- Resource use, other<br>- Mortality: in-hospital mortality
Wuerz, 2000[59]	2 academic hospitals, USA	60,000 (average)	32%	493 adults	ESI	- Hospital admission<br>- Resource use based on ESI<br>- Length of stay at the ED<br>- Costs
Wuerz, 2001[60]	Single academic hospital, USA	50,000	-	180 adults	ESI	- Mortality: 6 month mortality
Wuerz, 2001[61]	2 academic hospitals, USA	50,000-60,000	28%	8,251 adults	ESI	- Hospital admission<br>- Length of stay at the ED

Wulp, van der 2009[62]	4 teaching hospitals, the Netherlands	30,000-47,000	21%	72,232 children and adults	ESI and MTS	- Hospital admission<br>- Mortality at the ED
Wulp, van der, 2011[63]	Single academic hospital, the Netherlands	21,000	-	544 adults	ESI	- Worthing Phsyiological Scoring System<br>- Pain score
Yuksen, 2016[64]	Single academic hospital, Thailand	-	-	520 patients	ESI and 4-level nurse triage	- Hospital admission<br>- Resource use based on ESI<br>- Length of stay at the ED<br>- Mortality at the ED<br>- Immediate lifesaving interventions
Zachariasse, 2016[65]	Single academic children's hospital, the Netherlands	7,000	-	50,062 children	MTS	- ICU admission
Zachariasse, 2017[66]	1 academic children's hospital, 1 teaching hospital and 1 community hospital, the Netherlands and Portugal	7,000 – 190,000	-	222,603 adults and 66,060 children	MTS	- ICU admission<br>- Composite outcome based on physiologic parameters, patient disposition and resource use

ATS, Australasian Triage Scale; *CTAS*, Canadian Triage and Acuity Scale; *ESI*, Emergency Severity Index; *MTS*, Manchester Triage System; *Ped*, Pediatric; *SATS,* South African Triage Scale; *TTS*, Taiwan Triage System;

For peer review only

References

1. Aeimchanbanjong K, Pandee U. Validation of different pediatric triage systems in the emergency department. *World J Emerg Med.* 2017;8:223-227.
2. Al-Hindi AA, Al-Akhfash AA, Fareed AM, Alhusainan KS, Algasomy SF, Althowainy IR. Efficacy of implementation of a 5 scale pediatric triage and acuity scale in pediatric emergency, Saudi Arabia. *Saudi Medical Journal.* 2014;35:999-1004.
3. Allon R, Feldman O, Karminsky A, Steinberg C, Leiba R, Shavit I. Validity of the Pediatric Canadian Triage Acuity Scale in a tertiary children's hospital in Israel. *Eur J Emerg Med.* 2017.
4. Baumann MR, Strout TD. Evaluation of the Emergency Severity Index (version 3) triage algorithm in pediatric patients. *Acad Emerg Med.* 2005;12:219-224.
5. Baumann MR, Strout TD. Triage of Geriatric Patients in the Emergency Department: Validity and Survival With the Emergency Severity Index. *Ann Emerg Med.* 2007;49:234-240.
6. Brillman JC, Doezema D, Tandberg D, et al. Triage: Limitations in predicting need for emergent care and hospital admission. *ANN EMERG MED.* 1996;27:493-500.
7. Bruijns SR, Wallis LA, Burch VC. A prospective evaluation of the Cape triage score in the emergency department of an urban public hospital in South Africa. *Emerg Med J.* 2008;25:398-402.
8. Chang YC, Ng CJ, Wu CT, Chen LC, Chen JC, Hsu KH. Effectiveness of a five-level Paediatric Triage System: An analysis of resource utilisation in the emergency department in Taiwan. *Emerg Med J.* 2012.
9. Chi CH, Huang CM. Comparison of the Emergency Severity Index (ESI) and the Taiwan triage system in predicting resource utilization. *J Formos Med Assoc.* 2006;105:617-625.
10. Dong SL, Bullard MJ, Meurer DP, et al. Predictive Validity of a Computerized Emergency Triage Tool. *Acad Emerg Med.* 2007;14:16-21.
11. Dugas AF, Kirsch TD, Toerper M, et al. An Electronic Emergency Triage System to Improve Patient Distribution by Critical Outcomes. *J Emerg Med.* 2016;50:910-918.
12. Eitel DR, Travers DA, Rosenau AM, Gilboy N, Wuerz RC. The Emergency Severity Index triage algorithm version 2 is reliable and valid. *Acad Emerg Med.* 2003;10:1070-1080.
13. Engan M, Hirth A, Tronnes H. Validation of a Modified Triage Scale in a Norwegian Pediatric Emergency Department. *Int J Pediatr.* 2018;2018:4676758.
14. Erimşah ME, Yaka E, Yilmaz S, Kama A, Pekdemir M. Inter-rater reliability and validity of the Ministry of Health of Turkey's mandatory emergency triage instrument. *EMA Emerg Med Australas.* 2015;27:210-215.
15. Fan MMW, Leung LP. Validation of the Hong Kong accident and Emergency Triage Guidelines. *Hong Kong Med J.* 2013;19:198-202.
16. Fong RY, Glen WSS, Mohamed Jamil AK, Tam WWS, Kowitlawakul Y. Comparison of the Emergency Severity Index versus the Patient Acuity Category Scale in an emergency setting. *Int Emerg Nurs.* 2018;41:13-18.
17. Funakoshi H, Shiga T, Homma Y, et al. Validation of the modified Japanese Triage and Acuity Scale-based triage system emphasizing the physiologic variables or mechanism of injuries. *Int J Emer Med.* 2016;9:1-6.

For peer review only

18. Ganapathy S, Yeo JG, Thia XHM, Hei GMA, Tham LP. The Singapore Paediatric Triage Scale Validation Study. *Singapore Med J.* 2018;59:205-209.

19. Van Gerven R, Delooz H, Sermeus W. Systematic triage in the emergency department using the Australian National Triage Scale: a pilot project. *Eur J Emerg Med.* 2001;8:3-7.

20. Ghafarypour-Jahrom M, Taghizadeh M, Heidari K, Derakhshanfar H. Validity and Reliability of the Emergency Severity Index and Australasian Triage System in Pediatric Emergency Care of Mofid Children's Hospital in Iran. *Bull Emerg Trauma.* 2018;6:329-333.

21. Gouin S, Gravel J, Amre DK, Bergeron S. Evaluation of the Paediatric Canadian Triage and Acuity Scale in a pediatric ED. *Am J Emerg Med.* 2005;23:243-247.

22. Gräff I, Goldschmidt B, Glien P, et al. The German version of the Manchester triage system and its quality criteria - First assessment of validity and reliability. *PLoS ONE.* 2014;9.

23. Gravel J, Manzano S, Arsenault M. Validity of the Canadian Paediatric Triage and Acuity Scale in a tertiary care hospital. *Can J Emerg Med.* 2009;11:23-28.

24. Gravel J, Gouin S, Goldman RD, et al. The Canadian Triage and Acuity Scale for children: A prospective multicenter evaluation. *Ann Emerg Med.* 2012;60:71-77.

25. Gravel J, Fitzpatrick E, Gouin S, et al. Performance of the Canadian Triage and Acuity Scale for children: a multicenter database study. *Ann Emerg Med.* 2013;61:27-32 e23.

26. Green NA, Durani Y, Brecher D, Depiero A, Loiselle J, Attia M. Emergency severity index version 4: A valid and reliable tool in pediatric emergency department triage. *Pediatr Emerg Care.* 2012;28:753-757.

27. Hansen LH, Mogensen CB, Wittenhoff L, Skjot-Arkil H. The danish regions pediatric triage model has a limited ability to detect both critically ill children as well as children to be sent home without treatment - a study of diagnostic accuracy. *Scand J Trauma Resusc Emerg Med.* 2017;25:55.

28. Hay E, Bekerman L, Rosenberg G, Peled R. Quality assurance of nurse triage: Consistency of results over three years. *Am J Emerg Med.* 2001;19:113-117.

29. van Ierland Y, van Veen M, Huibers L, Giesen P, Moll HA. Validity of telephone and physical triage in emergency care: The Netherlands Triage System. *Fam Pract.* 2011;28:334-341.

30. Jobe J, Ghuysen A, Gerard P, Hartstein G, D'Orio V. Reliability and validity of a new French-language triage algorithm: The ELISA scale. *Emerg Med J.* 2013.

31. Lee A, Hazlett CB, Chow S, et al. How to minimize inappropriate utilization of Accident and Emergency Departments: Improve the validity of classifying the general practice cases amongst the A&E attendees. *Health Policy.* 2003;66:159-168.

32. Lee JY, Oh SH, Peck EH, et al. The validity of the Canadian Triage and Acuity Scale in predicting resource utilization and the need for immediate life-saving interventions in elderly emergency department patients. *Scand J Trauma Resusc Emerg Med.* 2011;19:68.

33. Lin GX, Yang YL, Kudirka D, et al. Implementation of a Pediatric Emergency Triage System in Xiamen, China. *Chin Med J (Engl).* 2016;129:2416-2421.

34. Ma W, Gafni A, Goldman RD. Correlation of the Canadian Pediatric Emergency Triage and Acuity Scale to ED resource utilization. *Am J Emerg Med.* 2008;26:893-897.

For peer review only

35. Maleki M, Fallah R, Riahi L, Delavari S, Rezaei S. Effectiveness of Five-Level Emergency Severity Index Triage System Compared With Three-Level Spot Check: An Iranian Experience. *Arch Trauma Res.* 2015;4:e29214.

36. Maningas PA, Hime DA, Parker DE. The use of the soterion rapid triage system in children presenting to the Emergency Department. *J Emerg Med.* 2006;31:353-359.

37. Maningas PA, Hime DA, Parker DE, McMurry TA. The Soterion Rapid Triage System: Evaluation of inter-rater reliability and validity. *J Emerg Med.* 2006;30:461-469.

38. Martins HM, Cuna LM, Freitas P. Is Manchester (MTS) more than a triage system? A study of its association with mortality and admission to a large Portuguese hospital. *Emerg Med J.* 2009;26:183-186.

39. Meyer GD, Meyer TN, Gaunt CB. Validity of the South African Triage Scale in a rural district hospital. *Afr J Emerg Med.* 2018;8:145-149.

40. Ng CJ, Hsu KH, Kuan JT, et al. Comparison between Canadian Triage and Acuity Scale and Taiwan Triage System in emergency departments. *J Formos Med Assoc.* 2010;109:828-837.

41. Ng CJ, Yen ZS, Tsai JCH, et al. Validation of the Taiwan triage and acuity scale: A new computerised five-level triage system. *Emerg Med J.* 2011;28:1026-1031.

42. Platts-Mills TF, Travers D, Biese K, et al. Accuracy of the emergency severity index triage instrument for identifying elder emergency department patients receiving an immediate life-saving intervention. *Acad Emerg Med.* 2010;17:238-243.

43. Roukema J, Steyerberg EW, van Meurs A, Ruige M, van der Lei J, Moll HA. Validity of the Manchester Triage System in paediatric emergency care. *Emerg Med J.* 2006;23:906-910.

44. Santos AP, Freitas P, Martins HMG. Manchester triage system version II and resource utilisation in emergency department. *Emerg Med J.* 2013.

45. Seiger N, van Veen M, Almeida H, et al. Improving the Manchester Triage System for pediatric emergency care: an international multicenter study. *PLoS One.* 2014;9:e83267.

46. Steiner D, Renetseder F, Kutz A, et al. Performance of the Manchester Triage System in Adult Medical Emergency Patients: A Prospective Cohort Study. *J Emerg Med.* 2016;50:678-689.

47. Storm-Versloot MN, Ubbink DT, Kappelhof J, Luitse JSK. Comparison of an informally structured triage system, the emergency severity index, and the manchester triage system to distinguish patient priority in the emergency department. *Acad Emerg Med.* 2011;18:822-829.

48. Storm-Versloot MN, Vermeulen H, van Lammeren N, Luitse JS, Goslings JC. Influence of the Manchester triage system on waiting time, treatment time, length of stay and patient satisfaction; a before and after study. *Emerg Med J.* 2014;31:13-18.

49. Taboulet P, Moreira V, Haas L, et al. Triage with the French Emergency Nurses Classification in Hospital scale: Reliability and validity. *Eur J Emerg Med.* 2009;16:61-67.

50. Tanabe P, Gimbel R, Yarnold PR, Adams JG. The Emergency Severity Index (version 3) 5-level triage system scores predict ED resource consumption. *J Emerg Nurs.* 2004;30:22-29.

51. Tanabe P, Gimbel R, Yarnold PR, Kyriacou DN, Adams JG. Reliability and Validity of Scores on the Emergency Severity Index Version 3. *Acad Emerg Med.* 2004;11:59-65.

For peer review only

52. Travers DA, Waller AE, Bowling JM, Flowers D, Tintinalli J. Five-level triage system more effective than three-level in tertiary emergency department. *J Emerg Nurs.* 2002;28:395-400.

53. Travers DA, Waller AE, Katznelson J, Agans R. Reliability and validity of the emergency severity index for pediatric triage. *Acad Emerg Med.* 2009;16:843-849.

54. Twomey M, Cheema B, Buys H, et al. Vital signs for children at triage: A multicentre validation of the revised South African Triage Scale (SATS) for children. *Samj South African Medical Journal.* 2013;103:304-308.

55. van Veen M, Steyerberg EW, Ruige M, et al. Manchester triage system in paediatric emergency care: prospective observational study. *BMJ.* 2008;337:a1501.

56. Van Veen M, Steyerberg EW, Van't Klooster M, et al. The Manchester triage system: Improvements for paediatric emergency care. *Emerg Med J.* 2012;29:654-659.

57. Waldrop RD, Harper DE, Mandry C. Prospective assessment of triage in an urban emergency department. *SOUTH MED J.* 1997;90:1208-1212.

58. Worster A, Fernandes CM, Eva K, Upadhye S. Predictive validity comparison of two five-level triage acuity scales. *Eur J Emerg Med.* 2007;14:188-192.

59. Wuerz RC, Milne LW, Eitel DR, Travers D, Gilboy N. Reliability and validity of a new five-level triage instrument. *Acad Emerg Med.* 2000;7:236-242.

60. Wuerz R, Eitel D, Gilboy N, et al. Emergency severity index triage category is associated with six-month survival. *Acad Emerg Med.* 2001;8:61-64.

61. Wuerz RC, Travers D, Gilboy N, Eitel DR, Rosenau A, Yazhari R. Implementation and refinement of the Emergency severity index. *Acad Emerg Med.* 2001;8:170-176.

62. van der Wulp I, Schrijvers AJP, van Stel HF. Predicting admission and mortality with the Emergency Severity Index and the Manchester Triage System: a retrospective observational study. *Emergency Medicine Journal.* 2009;26:506-509.

63. Van Der Wulp I, Rullmann HAA, Leenen LPH, Van Stel HF. Associations of the Emergency Severity Index triage categories with patients' vital signs at triage: A prospective observational study. *Emerg Med J.* 2011;28:1032-1035.

64. Yuksen C, Sawatmongkornkul S, Suttabuth S, Sawanyawisuth K, Sittichanbuncha Y. Emergency severity index compared with 4-level triage at the emergency department of Ramathibodi University Hospital. *Asian Biomed.* 2016;10:155-161.

65. Zachariasse JM, Kuiper JW, de Hoog M, Moll HA, van Veen M. Safety of the Manchester Triage System to Detect Critically Ill Children at the Emergency Department. *J Pediatr.* 2016;177:232-237.e231.

66. Zachariasse JM, Seiger N, Rood PPM, et al. Validity of the Manchester triage system in emergency care: A prospective observational study. *PLoS ONE.* 2017;12.

For peer review only

Appendix 3. Risk of bias assessment

Table 3.1 Risk of bias and applicability concerns summary: review authors' judgements about each domain for each included study

For peer review only

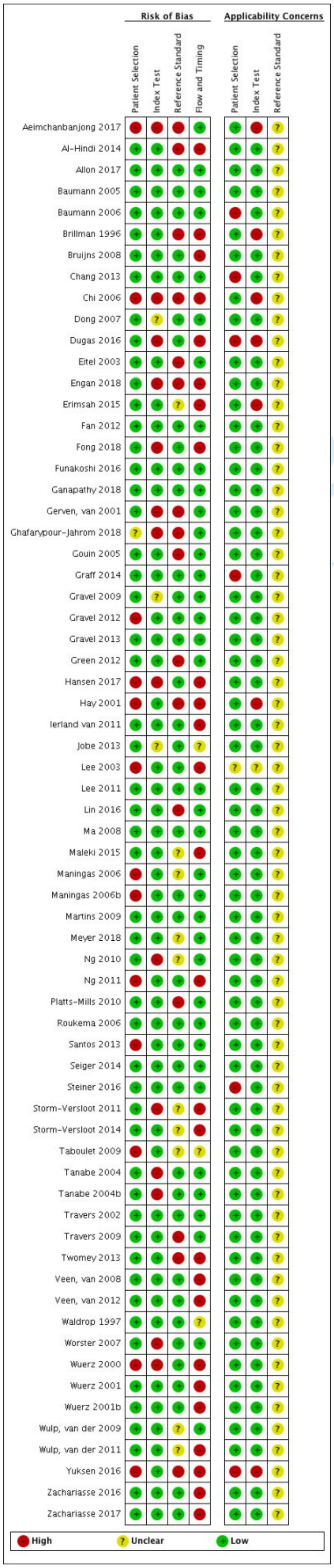

For peer review only

Appendix 4. Reference standards used in studies evaluating triage systems in the emergency department

**Patient disposition and follow-up**	Hospital admission<br>ICU admission<br>Follow-up at outpatient clinic or GP	47 studies<br>13 studies<br>1 study
**Waiting times**	Length of stay at the ED<br>Length of stay in hospital<br>Waiting time to physician<br>Other: Waiting time to nurse, Waiting time to examination, Waiting time to first lab results, Waiting time from arrival to treatment room, Waiting time from treatment room to discharge from ED, Waiting time for care	27 studies<br>5 studies<br>4 studies<br>1 study each
**Resource use**	Resource use based on ESI criteria<br>Costs<br>Current Procedural Terminology (CPT) codes<br>Other	14 studies<br>8 studies<br>2 studies<br>15 studies
**Mortality**	Mortality at the ED<br>In-hospital mortality<br>1 year mortality<br>Other: 30-day mortality, 60-day mortality, 6 month mortality	12 studies<br>5 studies<br>2 studies<br>1 study each
**Composite outcomes**	"Immediate lifesaving interventions"<br>"MTS reference standard": Combination of abnormal physiologic parameters, life-threatening conditions, resource use, follow-up<br>"Critical or time-sensitive outcomes": in-hospital mortality, intensive care unit admission or transfer to operating room or catheterization suite<br>"Actual clinical outcomes": Combination of life threatening condition, transfer to higher hospital, ICU, or hospital admission<br>Composite outcome based on physiologic parameters, patient disposition and resource use	4 studies<br>4 studies<br>1 study<br>1 study<br>1 study
**Physiologic parameters**	Worthing Phsyiological Scoring System<br>Pain score	1 study<br>1 study
**Other**	Expert opinion<br>Left without being seen<br>Patient or parent satisfaction<br>Level of prehospital care<br>Sentinel diagnosis<br>Pediatric risk of admission (PRISA) score	9 studies<br>5 studies<br>2 studies<br>1 study<br>1 study<br>1 study

For peer review only

Appendix 5. Reference standard per urgency category

Figure 5.1 Proportion of ICU admissions per urgency category in the most commonly evaluated triage systems.

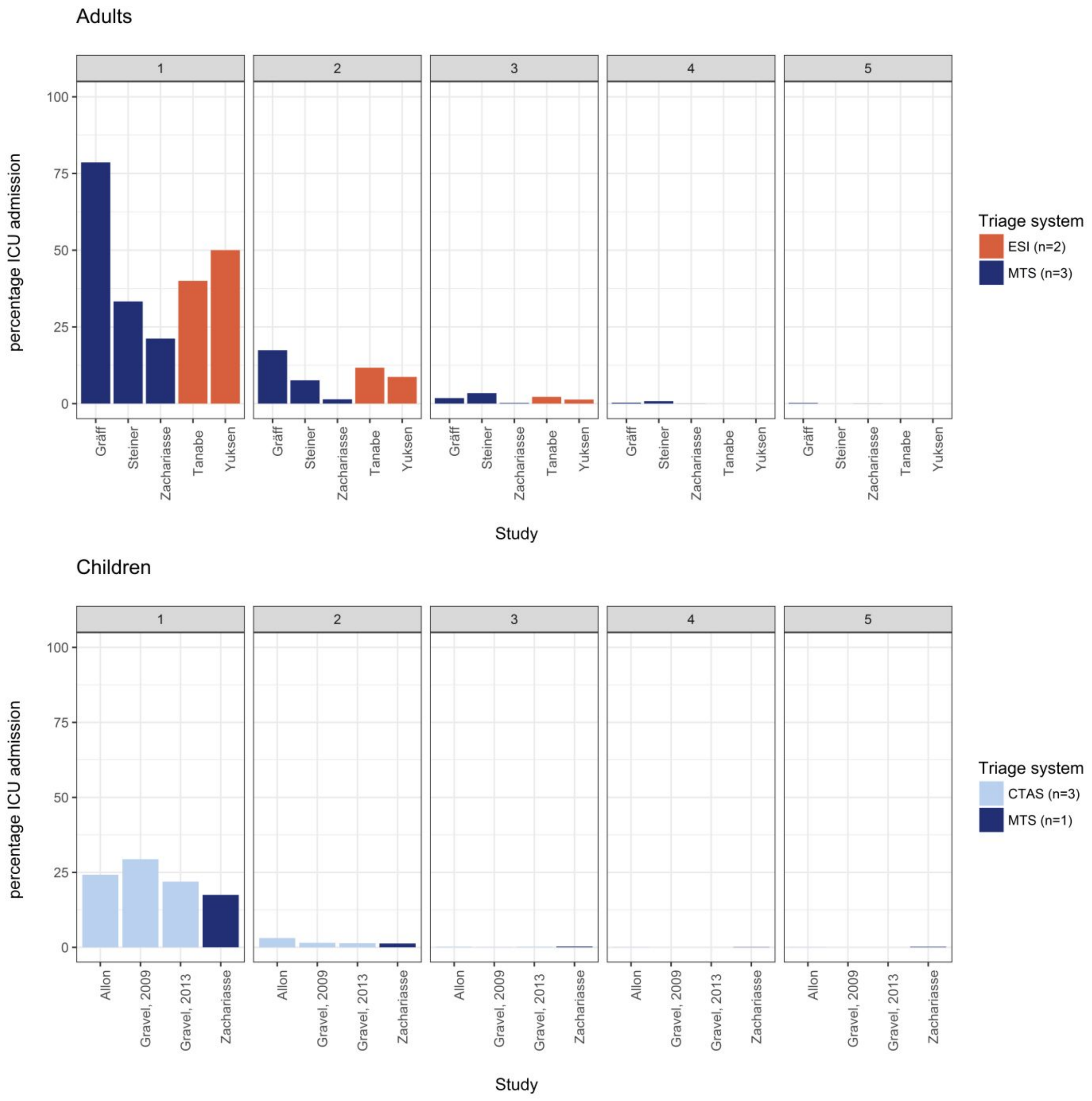

Figure 5.2 Proportion of patients discharged home per urgency category in the most commonly evaluated triage systems

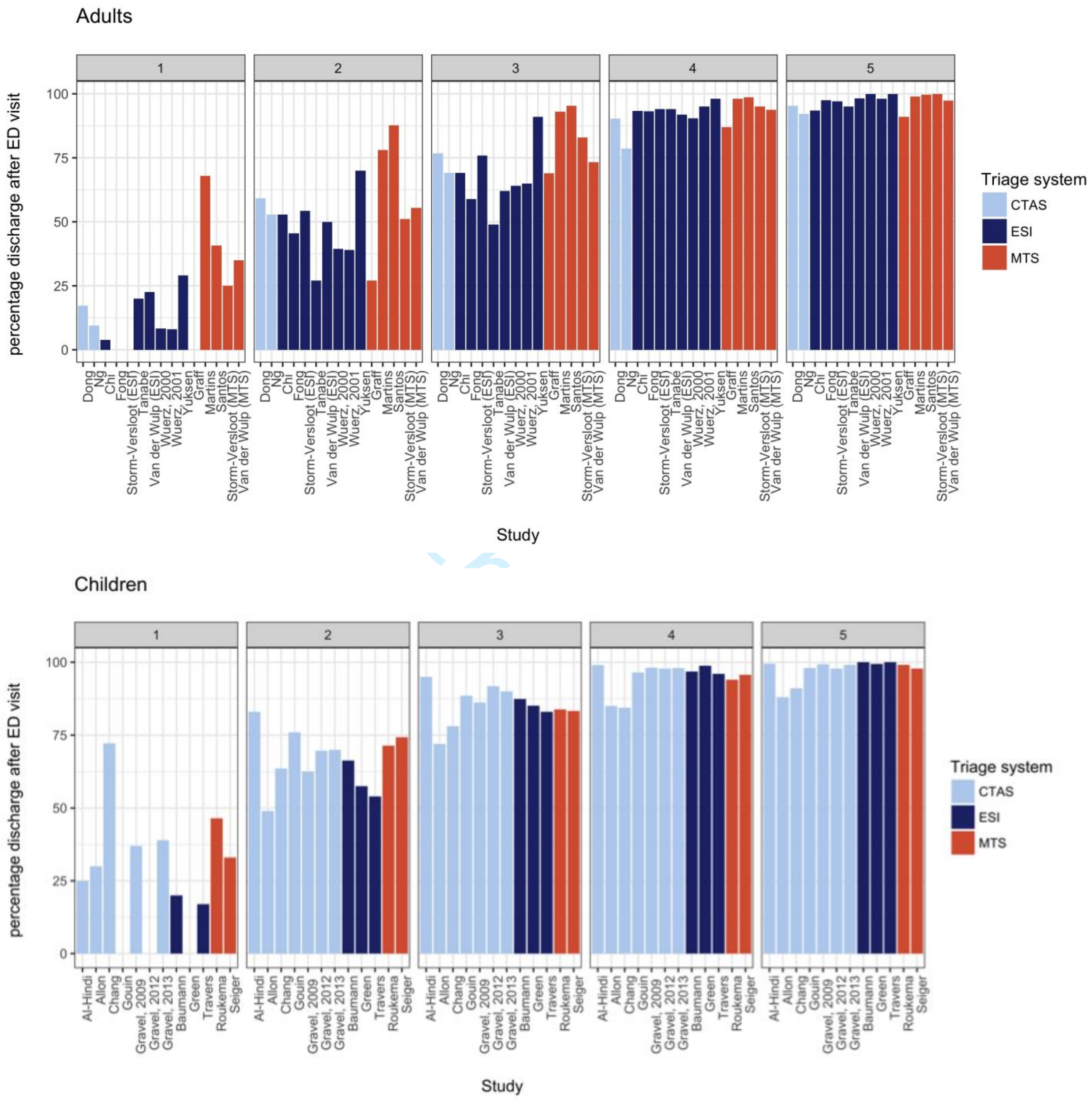

Appendix 6. Other reference standards

Other reference standards

14 studies used resource use according to ESI criteria as a reference standard of which 7 provided data to construct a 2x2 table and were conducted in one of the commonly used triage systems. One study evaluated resource use in the MTS, and all others studied the ESI.

Because the ESI states that resource prediction should only be used for the less acute patients, we used a different cut-off for dichotomization to calculate sensitivity and specificity. We dichotomized the triage categories into a high urgent group consisting of triage urgency levels 1, 2 and 3 with a low urgent group consisting of triage categories 4 and 5.

Overall, sensitivity of the triage systems to accurately classify patients with 2 or more resources in the highest urgency categories ranged from 0.75 (95%CI 0.68 to 0.81) to 0.91 (95%CI 0.87 to 0.94) in adults and from 0.76 (95%CI 0.70 to 0.82) to 0.90 (95%CI 0.87 to 0.92) in children (Fig. 7). Specificity of the triage system to accurately classify patients with 1 or 0 resources in the lowest two urgency categories ranged from 0.73 (95%CI 0.66 to 0.79) to 0.81 (95%CI 0.73 to 0.88) in adults and from 0.42 (95%CI 0.39 to 0.46) to 0.75 (95%CI 0.69 to 0.81) in children.

For peer review only

Fig 6.1. Forest plot evaluating sensitivity and specificity of triage systems using resource use according to ESI criteria as reference standard

ESI

Study	TP	FP	FN	TN	Age group	Sensitivity (95% CI)	Specificity (95% CI)
Baumann 2005	101	183	11	215	Children	0.90 [0.83, 0.95]	0.54 [0.49, 0.59]
Travers 2009	480	200	56	438	Children	0.90 [0.87, 0.92]	0.69 [0.65, 0.72]
Green 2012	158	206	49	367	Children	0.76 [0.70, 0.82]	0.64 [0.60, 0.68]
Wuerz 2000	241	59	36	157	Adults / Unspecified	0.87 [0.82, 0.91]	0.73 [0.66, 0.79]
Yuksen 2016	278	53	29	160	Adults / Unspecified	0.91 [0.87, 0.94]	0.75 [0.69, 0.81]
Fong 2018	134	23	45	98	Adults / Unspecified	0.75 [0.68, 0.81]	0.81 [0.73, 0.88]

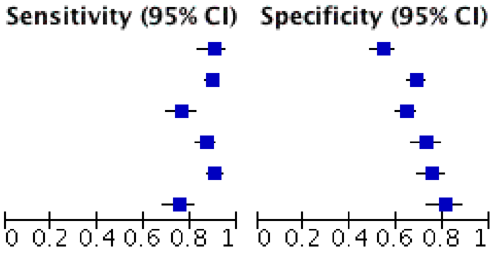

MTS

Study	TP	FP	FN	TN	Age group	Sensitivity (95% CI)	Specificity (95% CI)
Roukema 2006	205	469	51	340	Children	0.80 [0.75, 0.85]	0.42 [0.39, 0.46]

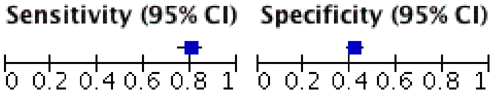

ESI = Emergency Severity Index; TP = true positive; FP = false positive; FN = false negative; TN = true negative; MTS = Manchester Triage System

view only

Appendix 7. Determinants of triage systems' performance

Figure 7.1. Forest plot evaluating sensitivity and specificity of triage systems for identifying low urgency patients as defined by discharge home after the ED visit, ordered by annual census as a marker of patient volume

Adults

Study	TP	FP	FN	TN	Annual Census	Sensitivity (95% CI)	Specificity (95% CI)
Graff 2014	6497	2132	8463	28377	23000.0	0.43 [0.43, 0.44]	0.93 [0.93, 0.93]
Storm-Versloot 2011	66	76	96	638	31000.0	0.41 [0.33, 0.49]	0.89 [0.87, 0.92]
Storm-Versloot 2011	73	72	76	651	31000.0	0.49 [0.41, 0.57]	0.90 [0.88, 0.92]
Yuksen 2016	38	68	22	392	33936.0	0.63 [0.50, 0.75]	0.85 [0.82, 0.88]
Van der Wulp 2009	2259	2146	5911	27658	38500.0	0.28 [0.27, 0.29]	0.93 [0.93, 0.93]
Van der Wulp 2009	2641	3183	4415	24019	38500.0	0.37 [0.36, 0.39]	0.88 [0.88, 0.89]
Wuerz 2001	1113	674	1260	5204	55000.0	0.47 [0.45, 0.49]	0.89 [0.88, 0.89]
Chi 2006	713	747	376	1336	58000.0	0.65 [0.63, 0.68]	0.64 [0.62, 0.66]
Wuerz 2000	91	53	68	281	60000.0	0.57 [0.49, 0.65]	0.84 [0.80, 0.88]
Dong 2007	1519	1947	4189	21869	67000.0	0.27 [0.25, 0.28]	0.92 [0.91, 0.92]
Tanabe 2004	129	48	75	151	70000.0	0.63 [0.56, 0.70]	0.76 [0.69, 0.82]
Santos 2013	485	3080	535	19515	100000.0	0.48 [0.44, 0.51]	0.86 [0.86, 0.87]
Martins 2009	18265	63803	12464	222090	128000.0	0.59 [0.59, 0.60]	0.78 [0.78, 0.78]

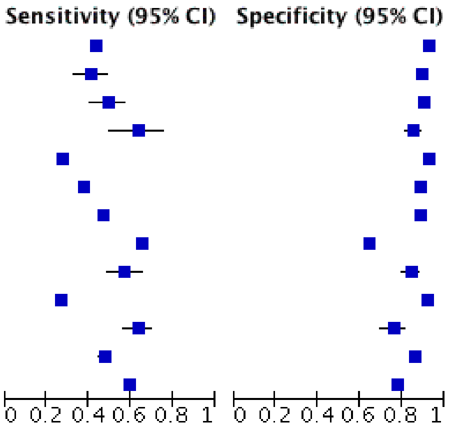

Children

Study	TP	FP	FN	TN	Annual Census	Sensitivity (95% CI)	Specificity (95% CI)
Aeimchanbanjong 2017	62	239	62	679	10000.0	0.50 [0.41, 0.59]	0.74 [0.71, 0.77]
Aeimchanbanjong 2017	64	165	59	753	10000.0	0.52 [0.43, 0.61]	0.82 [0.79, 0.84]
Aeimchanbanjong 2017	70	285	53	633	10000.0	0.57 [0.48, 0.66]	0.69 [0.66, 0.72]
Allon 2017	2631	2330	17726	60922	16722.0	0.13 [0.12, 0.13]	0.96 [0.96, 0.96]
Roukema 2006	147	256	62	600	30000.0	0.70 [0.64, 0.76]	0.70 [0.67, 0.73]
Chang 2013	3104	6821	6276	26327	42839.0	0.33 [0.32, 0.34]	0.79 [0.79, 0.80]
Green 2012	33	42	47	658	45000.0	0.41 [0.30, 0.53]	0.94 [0.92, 0.96]
Baumann 2005	44	66	28	372	53000.0	0.61 [0.49, 0.72]	0.85 [0.81, 0.88]
Gravel 2009	1937	2746	3124	50722	60000.0	0.38 [0.37, 0.40]	0.95 [0.95, 0.95]
Gouin 2005	8	19	38	472	65000.0	0.17 [0.08, 0.31]	0.96 [0.94, 0.98]
Al-Hindi 2014	65	295	45	2609	155000.0	0.59 [0.49, 0.68]	0.90 [0.89, 0.91]

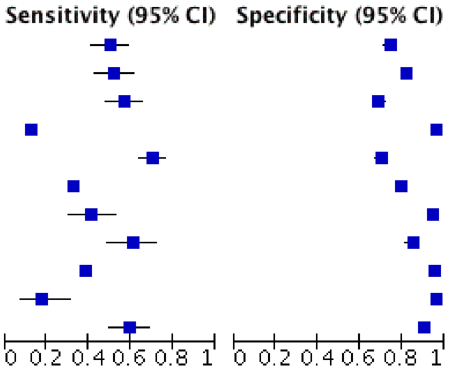

TP = true positive; FP = false positive; FN = false negative; TN = true negative

For peer review only

Figure 7.2. Forest plot evaluating sensitivity and specificity of triage systems for identifying low urgency patients as defined by discharge home after the ED visit, ordered by percentage hospitalization as a marker of case-mix severity of illness

Adults

Study	TP	FP	FN	TN	Hospitalization rate	Sensitivity (95% CI)	Specificity (95% CI)
Santos 2013	485	3080	535	19515	5.0	0.48 [0.44, 0.51]	0.86 [0.86, 0.87]
Martins 2009	18265	63803	12464	222090	10.0	0.59 [0.59, 0.60]	0.78 [0.78, 0.78]
Yuksen 2016	38	68	22	392	12.0	0.63 [0.50, 0.75]	0.85 [0.82, 0.88]
Storm-Versloot 2011	66	76	96	638	18.0	0.41 [0.33, 0.49]	0.89 [0.87, 0.92]
Storm-Versloot 2011	73	72	76	651	18.0	0.49 [0.41, 0.57]	0.90 [0.88, 0.92]
Dong 2007	1519	1947	4189	21869	19.0	0.27 [0.25, 0.28]	0.92 [0.91, 0.92]
Van der Wulp 2009	2259	2146	5911	27658	21.0	0.28 [0.27, 0.29]	0.93 [0.93, 0.93]
Van der Wulp 2009	2641	3183	4415	24019	21.0	0.37 [0.36, 0.39]	0.88 [0.88, 0.89]
Fong 2018	6	5	68	221	25.0	0.08 [0.03, 0.17]	0.98 [0.95, 0.99]
Wuerz 2001	1113	674	1260	5204	28.0	0.47 [0.45, 0.49]	0.89 [0.88, 0.89]
Wuerz 2000	91	53	68	281	32.0	0.57 [0.49, 0.65]	0.84 [0.80, 0.88]
Graff 2014	6497	2132	8463	28377	33.0	0.43 [0.43, 0.44]	0.93 [0.93, 0.93]
Chi 2006	713	747	376	1336	34.0	0.65 [0.63, 0.68]	0.64 [0.62, 0.66]

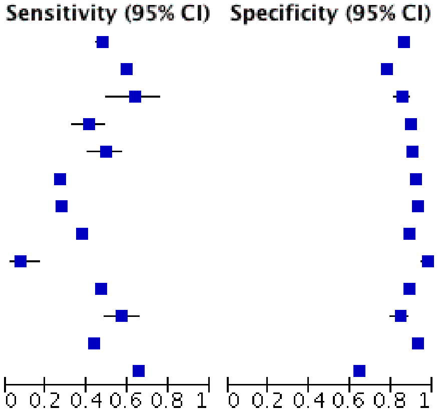

Children

Study	TP	FP	FN	TN	Hospitalization rate	Sensitivity (95% CI)	Specificity (95% CI)
Al-Hindi 2014	65	295	45	2609	4.0	0.59 [0.49, 0.68]	0.90 [0.89, 0.91]
Gouin 2005	8	19	38	472	9.0	0.17 [0.08, 0.31]	0.96 [0.94, 0.98]
Gravel 2009	1937	2746	3124	50722	9.0	0.38 [0.37, 0.40]	0.95 [0.95, 0.95]
Green 2012	33	42	47	658	10.0	0.41 [0.30, 0.53]	0.94 [0.92, 0.96]
Aeimchanbanjong 2017	62	239	62	679	12.0	0.50 [0.41, 0.59]	0.74 [0.71, 0.77]
Aeimchanbanjong 2017	64	165	59	753	12.0	0.52 [0.43, 0.61]	0.82 [0.79, 0.84]
Aeimchanbanjong 2017	70	285	53	633	12.0	0.57 [0.48, 0.66]	0.69 [0.66, 0.72]
Baumann 2005	44	66	28	372	14.0	0.61 [0.49, 0.72]	0.85 [0.81, 0.88]
Chang 2013	3104	6821	6276	26327	23.0	0.33 [0.32, 0.34]	0.79 [0.79, 0.80]
Allon 2017	2631	2330	17726	60922	24.0	0.13 [0.12, 0.13]	0.96 [0.96, 0.96]

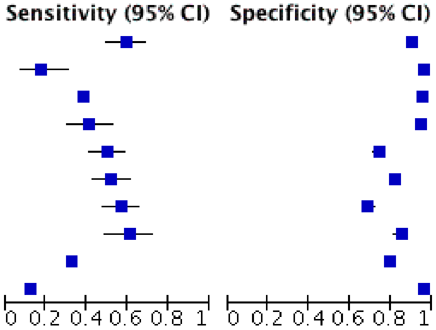

TP = true positive; FP = false positive; FN = false negative; TN = true negative

MOOSE Checklist for Meta-analyses of Observational Studies

Item No	Recommendation	Reported on Page No
Reporting of background should include		
1	Problem definition	p.6
2	Hypothesis statement	-
3	Description of study outcome(s)	p.6, 7, 8
4	Type of exposure or intervention used	p.6,7
5	Type of study designs used	p.7
6	Study population	p.6,7
Reporting of search strategy should include		
7	Qualifications of searchers (eg, librarians and investigators)	Title page
8	Search strategy, including time period included in the synthesis and key words	p.7, appendix 1
9	Effort to include all available studies, including contact with authors	p.7
10	Databases and registries searched	p.7, appendix 1
11	Search software used, name and version, including special features used (eg, explosion)	-
12	Use of hand searching (eg, reference lists of obtained articles)	Fig. 1
13	List of citations located and those excluded, including justification	Fig. 1, appendix 2
14	Method of addressing articles published in languages other than English	p.7
15	Method of handling abstracts and unpublished studies	p.7
16	Description of any contact with authors	-
Reporting of methods should include		
17	Description of relevance or appropriateness of studies assembled for assessing the hypothesis to be tested	p.7
18	Rationale for the selection and coding of data (eg, sound clinical principles or convenience)	p.7
19	Documentation of how data were classified and coded (eg, multiple raters, blinding and interrater reliability)	p.7
20	Assessment of confounding (eg, comparability of cases and controls in studies where appropriate)	-
21	Assessment of study quality, including blinding of quality assessors, stratification or regression on possible predictors of study results	p.8
22	Assessment of heterogeneity	p.9
23	Description of statistical methods (eg, complete description of fixed or random effects models, justification of whether the chosen models account for predictors of study results, dose-response models, or cumulative meta-analysis) in sufficient detail to be replicated	p.9
24	Provision of appropriate tables and graphics	Tables 1-2, Figs 1-9
Reporting of results should include		
25	Graphic summarizing individual study estimates and overall estimate	Figs 3,5,7
26	Table giving descriptive information for each study included	Appendix 2
27	Results of sensitivity testing (eg, subgroup analysis)	Figs 8-9
28	Indication of statistical uncertainty of findings	-

For peer review only

Item No	Recommendation	Reported on Page No
Reporting of discussion should include		
29	Quantitative assessment of bias (eg, publication bias)	p.29-30
30	Justification for exclusion (eg, exclusion of non-English language citations)	-
31	Assessment of quality of included studies	p.29-30
Reporting of conclusions should include		
32	Consideration of alternative explanations for observed results	p.30-31
33	Generalization of the conclusions (ie, appropriate for the data presented and within the domain of the literature review)	p.30-31
34	Guidelines for future research	p.31
35	Disclosure of funding source	p.3

From: Stroup DF, Berlin JA, Morton SC, et al, for the Meta-analysis Of Observational Studies in Epidemiology (MOOSE) Group. Meta-analysis of Observational Studies in Epidemiology. A Proposal for Reporting. *JAMA*. 2000;283(15):2008-2012. doi: 10.1001/jama.283.15.2008.

For peer review only
